# Supplementary material for: Drivers of malnutrition among late adolescent and young women in rural Pakistan: a cross-sectional assessment of the MaPPS trial
Source: BMJ Open. 2023 May 23;13(5):e063734. doi: 10.1136/bmjopen-2022-063734 (PMC10230942; doi:10.1136/bmjopen-2022-063734)
Supplement: Supplementary data [file bmjopen-2022-063734supp001.pdf]

Supplementary Material

Table S1. Comparison of social determinants of health with BMI categories (underweight [<18.5 kg/m^2], normal weight [18.5–24.9 kg/m^2; base], and overweight and obese [≥25 kg/m^2]) among late adolescent girls (15–18.9 y) enrolled in the MaPPS Trial at enrolment (n=14,771)[1]

		BMI categories							
		Crude				Multivariable adjusted			
**Possible determinants**	**n (%)**	Underweight	*P*	Overweight/ obese	*P*	Underweight	*P*	Overweight/ obese	*P*
Level 1: Structural factors – socioeconomic status									
Highest level of education									
None	6,297 (42.6)	(reference)	-	(reference)	-	-	-	-	-
Primary	3,652 (24.7)	0.96 (0.89 to 1.05)	0.39	1.46 (1.23 to 1.74)	<0.001	-	-	-	-
Secondary or higher	4,822 (32.6)	0.86 (0.80 to 0.93)	<0.001	2.05 (1.76 to 2.38)	<0.001	-	-	-	-
Occupation									
Unskilled manual labour	2,622 (17.8)	(reference)	-	(reference)	-	(reference)	-	(reference)	-
Skilled manual labour	2,762 (18.7)	0.85 (0.76 to 0.95)	0.004	2.44 (1.83 to 3.25)	<0.001	0.92 (0.79 to 1.07)	0.26	1.83 (1.42 to 2.36)	<0.001
Within the home	6,351 (43.0)	0.83 (0.75 to 0.91)	<0.001	3.28 (2.54 to 4.25)	<0.001	0.94 (0.83 to 1.07)	0.34	2.03 (1.61 to 2.58)	<0.001
Other[2]	3,036 (20.6)	0.90 (0.81 to 1.00)	0.06	3.26 (2.48 to 4.30)	<0.001	1.14 (0.94 to 1.37)	0.18	1.44 (1.09 to 1.92)	0.01
Religion									
Non-Muslim	1,483 (10.0)	(reference)	-	(reference)	-	-	-	-	-
Muslim	13,288 (90.0)	0.79 (0.71 to 0.88)	<0.001	1.96 (1.48 to 2.61)	<0.001	-	-	-	-
Wealth quintile									
Poorest	2,666 (18.0)	(reference)	-	(reference)	-	(reference)	-	(reference)	-
Poor	2,881 (19.5)	0.84 (0.76 to 0.94)	0.002	1.47 (1.07 to 2.01)	0.001	0.84 (0.74 to 0.95)	0.01	1.34 (1.07 to 1.67)	0.01
Middle	3,025 (20.5)	0.79 (0.71 to 0.88)	<0.001	2.36 (1.77 to 3.15)	<0.001	0.78 (0.68 to 0.89)	<0.001	2.07 (1.63 to 2.63)	<0.001
Rich	3,145 (21.3)	0.71 (0.64 to 0.79)	<0.001	3.42 (2.59 to 4.51)	<0.001	0.69 (0.58 to 0.82)	<0.001	2.94 (2.32 to 3.74)	<0.001
Richest	3,054 (20.7)	0.69 (0.62 to 0.77)	<0.001	5.18 (3.96 to 6.79)	<0.001	0.65 (0.56 to 0.74)	<0.001	4.65 (3.49 to 6.20)	<0.001
Level 2: Intermediary factors - household and personal characteristics									
Marital status									
Married	1,418 (9.6)	(reference)		(reference)		(reference)	-	(reference)	-
Unmarried	13,353 (90.4)	1.38 (1.23 to 1.56)	<0.001	0.80 (0.66 to 0.97)	<0.03	1.42 (1.28 to 1.59)	<0.001	0.71 (0.59 to 0.85)	<0.001
Ever been pregnant									
Yes	572 (3.9)	(reference)	-	(reference)	-	-	-	-	-
No	14,199 (96.1)	1.14 (0.96 to 1.36)	0.14	0.80 (0.59 to 1.08)	0.14	-	-	-	-
Household food security status									
Food insecure	3,861 (26.1)	(reference)		(reference)		(reference)	-	(reference)	-
Food secure	10,910 (73.9)	0.83 (0.77 to 0.90)	<0.001	1.92 (1.61 to 2.28)	<0.001	0.91 (0.83 to 0.99)	0.04	1.24 (1.03 to 1.51)	0.03
Level 3: Intermediary factors - health and well-being characteristics									
Perception of own health									
Poor or fair	1,533 (10.4)	(reference)		(reference)		(reference)	-	(reference)	-

Possible determinants	n (%)	BMI categories							
		Crude				Multivariable adjusted			
		Underweight	*P*	Overweight/ obese	*P*	Underweight	*P*	Overweight/ obese	*P*
Good	9,731 (65.9)	0.82 (0.74 to 0.92)	0.001	1.23 (0.97 to 1.57)	0.09	0.83 (0.70 to 0.97)	0.03	1.23 (0.89 to 1.68)	0.21
Excellent	3,507 (23.7)	0.71 (0.63 to 0.80)	<0.001	1.40 (1.08 to 1.81)	0.01	0.74 (0.62 to 0.88)	0.001	1.22 (0.93 to 1.60)	0.16
Experience of depression-like feelings									
Severe or extremely severe	687 (4.7)	(reference)	-	(reference)	-	-	-	-	-
Moderate	1,545 (10.5)	0.93 (0.78 to 1.12)	0.47	1.16 (0.78 to 1.71)	0.46	-	-	-	-
Mild	1,236 (8.4)	0.94 (0.78 to 1.14)	0.53	1.15 (0.76 to 1.72)	0.51	-	-	-	-
None	11,303 (76.5)	0.78 (0.67 to 0.92)	0.002	1.20 (0.85 to 1.68)	0.30	-	-	-	-
Experience of anxiety-like feelings									
Severe or extremely severe	1,893 (12.8)	(reference)	-	(reference)	-	-	-	-	-
Moderate	2,124 (14.4)	1.02 (0.90 to 1.16)	0.78	0.96 (0.74 to 1.25)	0.77	-	-	-	-
Mild	952 (6.4)	0.85 (0.72 to 1.00)	0.05	1.06 (0.77 to 1.44)	0.73	-	-	-	-
None	9,802 (66.4)	0.79 (0.72 to 0.88)	<0.001	1.04 (0.85 to 1.27)	0.72	-	-	-	-
Experience of stress-like feelings									
Severe or extremely severe	491 (3.3)	(reference)	-	(reference)	-	-	-	-	-
Moderate	778 (5.3)	0.94 (0.75 to 1.19)	0.61	0.88 (0.54 to 1.41)	0.59	-	-	-	-
Mild	885 (6.0)	0.91 (0.72 to 1.14)	0.40	1.07 (0.68 to 1.68)	0.78	-	-	-	-
None	12,617 (85.4)	0.80 (0.67 to 0.97)	0.02	1.03 (0.70 to 1.49)	0.89	-	-	-	-
Level 4: Intermediary factors - actions and practices-related characteristics									
Self-efficacy									
Low	5,695 (38.6)	(reference)	-	(reference)	-	(reference)	-	(reference)	-
Moderate	7,161 (48.5)	0.85 (0.79 to 0.92)	<0.001	1.27 (1.10 to 1.46)	0.001	0.91 (0.85 to 0.97)	<0.001	1.09 (0.93 to 1.27)	0.29
High	1,915 (13.0)	0.75 (0.67 to 0.83)	<0.001	1.12 (0.91 to 1.37)	0.29	0.82 (0.72 to 0.92)	<0.001	0.91 (0.73 to 1.14)	0.41
Decision-making autonomy									
All decisions made by family	8,244 (55.8)	(reference)	-	(reference)	-	-	-	-	-
Most decisions made by family	4,702 (31.8)	0.9 (0.84 to 0.97)	0.008	1.18 (1.03 to 1.36)	0.02	-	-	-	-
Decisions made jointly with family or autonomously	1,825 (12.4)	0.84 (0.76 to 0.94)	0.002	1.32 (1.10 to 1.60)	0.004	-	-	-	-
Skipping breakfast									
Skips breakfast	4,479 (30.3)	(reference)	-	(reference)	-	-	-	-	-
Eats breakfast	10,292 (69.7)	0.99 (0.92 to 1.07)	0.81	1.18 (1.02 to 1.36)	0.03	-	-	-	-
Eating dinner with family									
Never	4,021 (27.2)	(reference)	-	(reference)	-	-	-	-	-
Sometimes	1,869 (12.7)	0.99 (0.89 to 1.12)	0.93	1.12 (0.90 to 1.40)	0.30	-	-	-	-
Everyday	8,881 (60.1)	0.95 (0.88 to 1.03)	0.23	1.13 (0.97 to 1.32)	0.11	-	-	-	-

[1]Values are n (%) or RR (95% CI)
[2]Other category included those who identified themselves as students (2,988[20.2%]) or professionals (48[0.3%])

Table S2. Comparison of social determinants of health with BMI categories (underweight [<18.5 kg/m^2], normal weight [18.5–24.9 kg/m^2; base], and overweight and obese [≥25 kg/m^2]) among young women (19–23 y) enrolled in the MaPPS Trial at enrolment (n=10,676)

**Possible determinants**	**n (%)**	**BMI categories**							
		**Crude**				**Multivariable adjusted**			
		Underweight	*P*	Overweight/ obese	*P*	Underweight	*P*	Overweight/ obese	*P*
Level 1: Structural factors – socioeconomic status									
Highest level of education									
None	5,087 (47.6)	(reference)	-	(reference)	-	-	-	-	-
Primary	2,411 (22.6)	0.80 (0.71 to 0.89)	<0.001	1.71 (1.47 to 1.98)	<0.001	-	-	-	-
Secondary or higher	3,178 (29.8)	0.74 (0.66 to 0.82)	<0.001	2.21 (1.94 to 2.52)	<0.001	-	-	-	-
Occupation									
Unskilled manual labour	1,837 (17.2)	(reference)	-	(reference)	-	(reference)	-	(reference)	-
Skilled manual labour	2,372 (22.2)	0.81 (0.71 to 0.93)	0.002	2.77 (2.17 to 3.55)	<0.001	0.95 (0.83 to 1.09)	0.48	1.91 (1.48 to 2.48)	<0.001
Within the home	5,779 (54.1)	0.75 (0.67 to 0.84)	<0.001	3.52 (2.81 to 4.42)	<0.001	0.93 (0.83 to 1.05)	0.25	2.07 (1.61 to 2.67)	<0.001
Other[2]	688 (6.4)	0.76 (0.62 to 0.93)	0.007	3.80 (2.83 to 5.11)	<0.001	1.11 (0.83 to 1.49)	0.47	1.64 (1.19 to 2.26)	0.002
Religion									
Non-Muslim	919 (8.6)	(reference)	-	(reference)	-	(reference)	-	(reference)	-
Muslim	9,757 (91.4)	0.64 (0.56 to 0.74)	<0.001	1.74 (1.35 to 2.25)	<0.001	0.76 (0.61 to 0.93)	0.01	0.98 (0.71 to 1.36)	0.92
Wealth quintile									
Poorest	1,883 (17.6)	(reference)	-	(reference)	-	(reference)	-	(reference)	-
Poor	1,972 (18.5)	0.81 (0.71 to 0.92)	0.002	1.98 (1.48 to 2.64)	<0.001	0.87 (0.74 to 1.02)	0.09	1.81 (1.36 to 2.43)	<0.001
Middle	2,114 (19.8)	0.73 (0.64 to 0.83)	<0.001	3.31 (2.52 to 4.34)	<0.001	0.79 (0.70 to 0.89)	<0.001	2.84 (2.02 to 4.00)	<0.001
Rich	2,215 (20.7)	0.62 (0.54 to 0.71)	<0.001	4.68 (3.60 to 6.09)	<0.001	0.68 (0.59 to 0.78)	<0.001	3.88 (2.85 to 5.28)	<0.001
Richest	2,492 (23.3)	0.55 (0.48 to 0.63)	<0.001	6.36 (4.92 to 8.22)	<0.001	0.59 (0.48 to 0.72)	<0.001	5.24 (3.93 to 6.98)	<0.001
Level 2: Intermediary factors - household and personal characteristics									
Marital status									
Married	4,267 (40.0)	(reference)	-	(reference)	-	(reference)	-	(reference)	-
Unmarried	6,409 (60.0)	1.14 (1.04 to 1.24)	0.004	0.90 (0.81 to 1.01)	0.08	1.25 (1.11 to 1.42)	<0.001	0.77 (0.69 to 0.85)	<0.001
Ever been pregnant									
Yes	2,906 (27.2)	(reference)	-	(reference)	-	-	-	-	-
No	7,770 (72.8)	1.03 (0.93 to 1.13)	0.58	0.93 (0.82 to 1.06)	0.28	-	-	-	-
Household food security status									
Food insecure	2,494 (23.4)	(reference)	-	(reference)	-	-	-	-	-
Food secure	8,182 (76.6)	0.79 (0.71 to 0.87)	<0.001	1.77 (1.52 to 2.07)	<0.001	-	-	-	-
Level 3: Intermediary factors - health and well-being characteristics									
Perception of own health									
Poor or fair	1,275 (11.9)	(reference)	-	(reference)	-	(reference)	-	(reference)	-
Good	6,828 (64.0)	0.78 (0.68 to 0.89)	<0.001	1.18 (0.97 to 1.44)	0.09	0.78 (0.69 to 0.88)	<0.001	1.16 (0.94 to 1.44)	0.18
Excellent	2,573 (24.1)	0.75 (0.64 to 0.87)	<0.001	1.35 (1.09 to 1.67)	0.006	0.78 (0.69 to 0.89)	<0.001	1.16 (0.89 to 1.54)	0.27
Experience of depression-like feelings									
Severe or extremely severe	537 (5.0)	(reference)	-	(reference)	-	-	-	-	-

Possible determinants	n (%)	BMI categories							
		Crude				Multivariable adjusted			
		Underweight	*P*	Overweight/ obese	*P*	Underweight	*P*	Overweight/ obese	*P*
Moderate	1,108 (10.4)	1.02 (0.82 to 1.28)	0.84	1.37 (0.97 to 1.93)	0.08	-	-	-	-
Mild	922 (8.6)	1.01 (0.8 to 1.28)	0.92	1.57 (1.11 to 2.23)	0.01	-	-	-	-
None	8,109 (76.0)	0.87 (0.72 to 1.06)	0.16	1.49 (1.11 to 2.01)	0.009	-	-	-	-
Experience of anxiety-like feelings									
Severe or extremely severe	1,421 (13.3)	(reference)	-	(reference)	-	-	-	-	-
Moderate	1,514 (14.2)	0.94 (0.80 to 1.10)	0.44	0.91 (0.73 to 1.15)	0.44	-	-	-	-
Mild	732 (6.9)	0.71 (0.58 to 0.87)	0.001	0.72 (0.54 to 0.97)	0.03	-	-	-	-
None	7,009 (65.7)	0.81 (0.72 to 0.92)	0.001	1.17 (0.98 to 1.40)	0.08	-	-	-	-
Experience of stress-like feelings									
Severe or extremely severe	405 (3.8)	(reference)	-	(reference)	-	-	-	-	-
Moderate	578 (5.4)	1.15 (0.87 to 1.52)	0.33	1.07 (0.72 to 1.59)	0.74	-	-	-	-
Mild	621 (5.8)	0.96 (0.72 to 1.26)	0.76	1.24 (0.85 to 1.80)	0.27	-	-	-	-
None	9,072 (85.0)	0.88 (0.71 to 1.10)	0.27	1.08 (0.79 to 1.47)	0.62	-	-	-	-
Level 4: Intermediary factors - actions and practices-related characteristics									
Self-efficacy									
Low	2,937 (27.5)	(reference)	-	(reference)	-	-	-	-	-
Moderate	5,709 (53.5)	0.92 (0.83 to 1.02)	0.10	1.34 (1.16 to 1.54)	<0.001	-	-	-	-
High	2,030 (19.0)	0.83 (0.73 to 0.95)	0.005	1.40 (1.18 to 1.66)	<0.001	-	-	-	-
Decision-making autonomy									
All decisions made by family	4,365 (40.9)	(reference)	-	(reference)	-	(reference)	-	(reference)	-
Most decisions made by family	3,630 (34.0)	0.91 (0.83 to 1.01)	0.08	1.08 (0.95 to 1.24)	0.23	0.92 (0.85 to 0.99)	0.03	1.08 (0.93 to 1.27)	0.31
Decisions made jointly with family or autonomously	2,681 (25.1)	0.85 (0.76 to 0.94)	0.003	1.19 (1.04 to 1.37)	0.01	0.85 (0.77 to 0.95)	0.003	1.24 (1.06 to 1.45)	0.01
Skipping breakfast									
Skips breakfast	3,130 (29.3)	(reference)	-	(reference)	-	-	-	-	-
Eats breakfast	7,546 (70.7)	0.93 (0.85 to 1.02)	0.11	1.15 (1.01 to 1.31)	0.03	-	-	-	-
Eating dinner with family									
Never	2,800 (26.2)	(reference)	-	(reference)	-	-	-	-	-
Sometimes	1,620 (15.2)	1.15 (1 to 1.32)	0.05	0.97 (0.81 to 1.17)	0.75	-	-	-	-
Everyday	6,256 (58.6)	1.13 (1.02 to 1.25)	0.02	1.08 (0.94 to 1.23)	0.27	-	-	-	-

[1]Values are n (%) or RR (95% CI)

[2]Other category included those who identified themselves as a student (526[4.9%]) or professional (688[6.4%])

Table S3. Comparison of social determinants of health with stunting (height <145 cm) among late adolescent girls (n=14,771) and adult women (n=10,676) enrolled in the MaPPS Trial at enrolment

Possible determinants	Stunting among late adolescent girls				Stunting among young women			
	Crude		Multivariable adjusted		Crude		Multivariable adjusted	
	OR (95% CI)	*P*	OR (95% CI)	*P*	OR (95% CI)	*P*	OR (95% CI)	*P*
Level 1: Structural factors – socioeconomic status								
Highest level of education								
None	(reference)	-	(reference)	-	(reference)	-	(reference)	-
Primary	0.65 (0.57 to 0.74)	<0.001	0.76 (0.62 to 0.94)	0.01	0.62 (0.51 to 0.74)	<0.001	0.76 (0.63 to 0.93)	0.006
Secondary or higher	0.33 (0.29 to 0.38)	<0.001	0.48 (0.39 to 0.59)	<0.001	0.51 (0.43 to 0.61)	<0.001	0.79 (0.66 to 0.95)	0.01
Occupation								
Unskilled manual labour	(reference)	-	-	-	(reference)	-	-	-
Skilled manual labour	0.56 (0.47 to 0.66)	<0.001	-	-	0.75 (0.61 to 0.91)	0.005	-	-
Within the home	0.69 (0.60 to 0.79)	<0.001	-	-	0.59 (0.49 to 0.71)	<0.001	-	-
Other[1]	0.32 (0.27 to 0.39)	<0.001	-	-	0.45 (0.32 to 0.65)	<0.001	-	-
Religion								
Non-Muslim	(reference)	-	-	-	(reference)	-	-	-
Muslim	0.57 (0.49 to 0.67)	<0.001	-	-	0.74 (0.59 to 0.92)	0.008	-	-
Wealth quintile								
Poorest	(reference)	-	(reference)	-	(reference)	-	(reference)	-
Poor	0.83 (0.72 to 0.97)	0.02	0.91 (0.77 to 1.08)	0.29	0.88 (0.72 to 1.08)	0.22	0.92 (0.73 to 1.17)	0.52
Middle	0.67 (0.57 to 0.78)	<0.001	0.79 (0.65 to 0.96)	0.02	0.65 (0.52 to 0.80)	<0.001	0.70 (0.59 to 0.84)	<0.001
Rich	0.46 (0.39 to 0.55)	<0.001	0.63 (0.49 to 0.81)	<0.001	0.48 (0.38 to 0.60)	<0.001	0.54 (0.40 to 0.74)	<0.001
Richest	0.26 (0.21 to 0.32)	<0.001	0.42 (0.35 to 0.51)	<0.001	0.34 (0.27 to 0.43)	<0.001	0.41 (0.29 to 0.57)	<0.001
Level 2: Intermediary factors - household and personal characteristics								
Marital status								
Married	(reference)	-	-	-	(reference)	-	-	-
Unmarried	0.89 (0.75 to 1.07)	0.21	-	-	0.99 (0.86 to 1.15)	0.93	-	-
Ever been pregnant								
Yes	(reference)	-	-	-	(reference)	-	(reference)	-
No	0.97 (0.74 to 1.27)	0.81	-	-	1.13 (0.96 to 1.32)	0.15	1.27 (1.10 to 1.47)	0.001
Household food security status								
Food insecure	(reference)	-	(reference)	-	(reference)	-	(reference)	-
Food secure	0.58 (0.51 to 0.64)	<0.001	0.77 (0.66 to 0.90)	0.001	0.57 (0.49 to 0.66)	<0.001	0.73 (0.59 to 0.90)	0.003
Level 3: Intermediary factors - health and well-being characteristics								
Perception of own health								
Poor or fair	(reference)	-	-	-	(reference)	-	-	-
Good	0.93 (0.78 to 1.10)	0.38	-	-	0.83 (0.67 to 1.02)	0.07	-	-
Excellent	0.85 (0.70 to 1.04)	0.11	-	-	0.79 (0.62 to 1.00)	0.06	-	-
Experience of depression-like feelings								
Severe or extremely severe	(reference)	-	-	-	(reference)	-	-	-
Moderate	0.93 (0.70 to 1.23)	0.61	-	-	0.98 (0.68 to 1.41)	0.90	-	-
Mild	0.94 (0.71 to 1.26)	0.70	-	-	0.86 (0.58 to 1.26)	0.43	-	-

BMJ Publishing Group Limited (BMJ) disclaims all liability and responsibility arising from any reliance placed on this supplemental material which has been supplied by the author(s)

**Possible determinants**	**Stunting among late adolescent girls**				**Stunting among young women**			
	Crude		Multivariable adjusted		Crude		Multivariable adjusted	
	OR (95% CI)	*P*	OR (95% CI)	*P*	OR (95% CI)	*P*	OR (95% CI)	*P*
None	0.79 (0.62 to 1.00)	0.05	-	-	0.88 (0.64 to 1.19)	0.40	-	-
Experience of anxiety-like feelings								
Severe or extremely severe	(reference)	-	-	-	(reference)	-	-	-
Moderate	0.84 (0.69 to 1.03)	0.09	-	-	0.82 (0.62 to 1.07)	0.14	-	-
Mild	0.96 (0.75 to 1.23)	0.76	-	-	0.99 (0.72 to 1.37)	0.97	-	-
None	0.83 (0.71 to 0.97)	0.02	-	-	0.94 (0.77 to 1.16)	0.56	-	-
Experience of stress-like feelings								
Severe or extremely severe	(reference)	-	-	-	(reference)	-	-	-
Moderate	0.93 (0.64 to 1.34)	0.69	-	-	0.93 (0.58 to 1.49)	0.76	-	-
Mild	1.09 (0.77 to 1.54)	0.63	-	-	0.78 (0.48 to 1.25)	0.30	-	-
None	0.90 (0.68 to 1.20)	0.49	-	-	0.98 (0.68 to 1.40)	0.90	-	-
Level 4: Intermediary factors - actions and practices-related characteristics								
Self-efficacy								
Low	(reference)	-	(reference)		(reference)	-	-	-
Moderate	0.62 (0.56 to 0.70)	<0.001	0.74 (0.64 to 0.84)	<0.001	0.83 (0.71 to 0.98)	0.03	-	-
High	0.59 (0.50 to 0.71)	<0.001	0.74 (0.62 to 0.87)	<0.001	0.78 (0.63 to 0.97)	0.03	-	-
Decision-making autonomy								
All decisions made by family	(reference)	-	(reference)	-	(reference)	-	-	-
Most decisions made by family	0.83 (0.74 to 0.94)	0.003	0.88 (0.76 to 1.01)	0.08	0.99 (0.84 to 1.16)	0.90	-	-
Decisions made jointly with family or autonomously	0.74 (0.62 to 0.89)	0.001	0.76 (0.62 to 0.92)	0.01	0.85 (0.71 to 1.02)	0.09	-	-
Skipping breakfast								
Skips breakfast	(reference)	-	-	-	(reference)	-	-	-
Eats breakfast	0.82 (0.73 to 0.91)	<0.001	-	-	0.79 (0.68 to 0.91)	0.002	-	-
Eating dinner with family								
Never	(reference)		-	-	(reference)	-	-	-
Sometimes	1.09 (0.92 to 1.30)	0.33	-	-	1.19 (0.95 to 1.49)	0.12	-	-
Everyday	0.93 (0.82 to 1.05)	0.23	-	-	1.02 (0.87 to 1.21)	0.79	-	-

[1]Other category included those who identified themselves as a student or profession
