# Supplementary material for: Drivers of malnutrition among late adolescent and young women in rural Pakistan: a cross-sectional assessment of the MaPPS trial
Source: BMJ Open. 2023 May 23;13(5):e063734. doi: 10.1136/bmjopen-2022-063734 (PMC10230942; doi:10.1136/bmjopen-2022-063734)
Supplement: Author's manuscript [file bmjopen-2022-063734.draft_revisions.pdf]

BMJ Open

BMJ Open is committed to open peer review. As part of this commitment we make the peer review history of every article we publish publicly available.

When an article is published we post the peer reviewers' comments and the authors' responses online. We also post the versions of the paper that were used during peer review. These are the versions that the peer review comments apply to.

The versions of the paper that follow are the versions that were submitted during the peer review process. They are not the versions of record or the final published versions. They should not be cited or distributed as the published version of this manuscript.

BMJ Open is an open access journal and the full, final, typeset and author-corrected version of record of the manuscript is available on our site with no access controls, subscription charges or pay-per-view fees (http://bmjopen.bmj.com).

If you have any questions on BMJ Open's open peer review process please email info.bmjopen@bmj.com

BMJ Open

Drivers of malnutrition among late adolescent and young women in rural Pakistan: A cross-sectional assessment of the MaPPS Trial

Journal:	*BMJ Open*
Manuscript ID	bmjopen-2022-063734
Article Type:	Original research
Date Submitted by the Author:	12-Apr-2022
Complete List of Authors:	Baxter, Jo-Anna; The Hospital for Sick Children,<br>Wasan, Yaqub; Aga Khan University, Centre of Excellence in Women and Child Health<br>Hussain, Amjad; Aga Khan University, Pediatrics and Child Health<br>Soofi, Sajid; The Aga Khan University, Pediatrics & Child Health<br>Ahmed, Imran; Aga Khan University<br>Bhutta, Zulfiqar; The Aga Khan University, Division of Women and Child Health
Keywords:	NUTRITION & DIETETICS, PUBLIC HEALTH, Community child health < PAEDIATRICS

SCHOLARONE™
Manuscripts

I, the Submitting Author has the right to grant and does grant on behalf of all authors of the Work (as defined in the below author licence), an exclusive licence and/or a non-exclusive licence for contributions from authors who are: i) UK Crown employees; ii) where BMJ has agreed a CC-BY licence shall apply, and/or iii) in accordance with the terms applicable for US Federal Government officers or employees acting as part of their official duties; on a worldwide, perpetual, irrevocable, royalty-free basis to BMJ Publishing Group Ltd ("BMJ") its licensees and where the relevant Journal is co-owned by BMJ to the co-owners of the Journal, to publish the Work in this journal and any other BMJ products and to exploit all rights, as set out in our licence.

The Submitting Author accepts and understands that any supply made under these terms is made by BMJ to the Submitting Author unless you are acting as an employee on behalf of your employer or a postgraduate student of an affiliated institution which is paying any applicable article publishing charge ("APC") for Open Access articles. Where the Submitting Author wishes to make the Work available on an Open Access basis (and intends to pay the relevant APC), the terms of reuse of such Open Access shall be governed by a Creative Commons licence – details of these licences and which Creative Commons licence will apply to this Work are set out in our licence referred to above.

Other than as permitted in any relevant BMJ Author's Self Archiving Policies, I confirm this Work has not been accepted for publication elsewhere, is not being considered for publication elsewhere and does not duplicate material already published. I confirm all authors consent to publication of this Work and authorise the granting of this licence.

For peer review only

TITLE PAGE

Title:

Drivers of malnutrition among late adolescent and young women in rural Pakistan: A cross-sectional assessment of the MaPPS Trial

Authors:

Jo-Anna B. Baxter[1,2], Yaqub Wasan[3], Amjad Hussain[3], Sajid B. Soofi[3], Imran Ahmed[3], Zulfiqar A. Bhutta[1-4]

Affiliations:

[1]Centre for Global Child Health, Hospital for Sick Children, Toronto, Canada

[2]Department of Nutritional Sciences, University of Toronto, Toronto, Canada

[3]Centre of Excellence in Women and Child Health, Aga Khan University, Karachi, Pakistan

[4] Dalla Lana School of Public Health, University of Toronto, Toronto, Canada

Corresponding author:

Dr. Zulfiqar A. Bhutta

Telephone number: +92 21 34930051

Email address: zulfiqar.bhutta@aku.edu

Postal address: Center of Excellence in Women and Child Health, Aga Khan University, Karachi 74800, Pakistan

Word count:

For peer review only

3884

Tables:

4

Figures:

0

Online supplemental material submitted:

Yes

ARTICLE SUMMARY

Abstract:

Objective: This study aimed to characterize the burden of malnutrition and assess how underlying determinants at the structural and intermediary levels contributed to malnutrition among late adolescent and young women in rural Pakistan.

Design: Cross-sectional enrolment data assessment.

Setting and participants: This study was conducted using data from adolescent and young women (n=25,447) enrolled in the Matiari emPowerment and Preconception Supplementation (MaPPS) Trial, collected from June 2017 to July 2018 in Matiari District, Pakistan. The WHO-based cut-offs were applied to anthropometric measures to estimate BMI categories

For peer review only

(underweight, overweight, obese) and stunting. Hierarchical models were generated to evaluate the association between the determinants with BMI categories and stunting among late adolescent girls and young women, respectively.

Primary and secondary outcome measures: The main outcomes of interest were BMI categories and stunting. Explanatory variables included measures of socioeconomic status, education, occupation, health, well-being, food security, empowerment, and food practices.

Results: Regardless of age group, the prevalence of underweight was high (36.9%; 95% CI: 36.3 to 37.5%). More late adolescent girls were underweight, while more young women were overweight/obese ($P<0.001$). Stunting affected 9.2% (95% CI: 8.9 to 9.6%) of participants, of which 35.7% were additionally underweight and 7.3% overweight/obese. Compared to those in the normal weight category, those underweight were more likely to be impoverished and less empowered. Those overweight/obese were more likely to be from a higher wealth quintile and food secure. Increased education level and food security were associated with reductions stunting risk.

Conclusions: This study informs the data gap and need for comprehensive research on adolescent nutritional status. Findings suggest factors related to poverty played an important, underlying role in undernutrition among participants. Commitment to improving the nutritional status of all adolescent and young women in Pakistan will be critical given the observed burden of malnutrition.

Trial registration: Clinicaltrials.gov NCT03287882.

Strengths and limitations of this study:

- Although the catchment area of this study was restricted to the areas covered by the LHW Programme, the study is very large and representative of rural setting in Pakistan.

For peer review only

- Given the cross-sectional design of the study, we cannot suggest causation.
- All anthropometric measurements (weight, height, middle upper arm circumference) were collected in duplicate following standardized procedures by trained study personnel, although we did not collect additional measures of body composition (e.g., skin-fold thickness, abdominal circumference).
- We used validated questionnaires to assess social determinants of health at both the structural and intermediary levels.

KEYWORDS

body mass index, stunting, adolescent girl, young woman, undernutrition, underweight, overweight, obesity

For peer review only

INTRODUCTION

Adolescence is a life stage characterized by substantial physical, cognitive, social, and emotional development, and nutritional status importantly underlies the transition from childhood to adulthood.[1] Defined by the World Health Organization (WHO) as the period from 10–19 y, some suggest adolescence extends to 24 y given continued cortical development.[1 2] Being malnourished during the adolescent period is associated with poor health outcomes throughout the life-course and has potential intergenerational effects.[3 4] In South Asia, undernutrition affects >38% of adolescent girls; there has been limited decline in the past decade and the increasing occurrence of overweight/obesity is of additional concern.[5]

Anthropometric measures can serve as a useful indicator for assessing malnutrition. Measures of undernutrition, including underweight (thinness) and stunting (short stature), are determined from body mass index (BMI) and attained height, respectively. While underweight reflects current nutritional status caused by insufficient or imbalanced intake and/or underlying disease, stunting reflects chronic or recurrent undernourishment.[6] Being underweight and/or stunted has been found to be associated with adverse health consequences to the individual, and, in the event of a pregnancy, increased risk for adverse pregnancy outcomes.[7] Conversely, overweight and obesity have been linked to the early onset of non-communicable diseases and premature mortality.[8 9]

Social determinants of health (SDoH) are described to influence nutritional inequalities, making understanding their role in malnutrition of importance.[10] SDoH are typically divided into two classes, structural and intermediary.[11] Structural SDoH underlie food availability and access, including stratifiers such as ethnicity, education, occupation, and income. Intermediary SDoH are

For peer review only

more proximal to nutrition-related outcomes, affecting material circumstances and biological, psychological, and behavioural factors.[12]

With the focus of the Sustainable Development Goals on equity, and the explicit aim in Goal 5 to empower all women and girls,[13] measuring, monitoring, and researching women's empowerment is of importance. Adolescent girls are an understudied population with respect to empowerment, and understanding the impact of interventions that aim to improve empowerment is a noted research gap.[1] Proxies for empowerment span SDoH at both the structural (e.g., education) and intermediary levels (e.g., self-efficacy and contribution to decision-making). They are suggested to influence nutritional status, although this association has predominantly been made between maternal empowerment and child nutritional status.[14-16] The WHO acknowledges that empowering adolescent girls, families, and communities is an important component to improving overall health and nutritional status.[1]

At this time, there are more young people than ever before in Pakistan, with around 30% of the population between 15-24 y.[17] Nationally representative surveys have traditionally focused on married women 15–49 y given the importance of reproductive outcomes, and estimate that the prevalence of underweight and stunting are high at 8.5 and 5.0%, respectively, and that there is an emerging burden of overweight and obesity.[17] However, there has been limited assessment among late adolescent girls and unmarried young women, thus there is a gap in information within this segment of the population. We aimed to assess the prevalence of malnutrition using anthropometric measures from all adolescent and young women (15–23 y) living in rural Pakistan, and determine whether structural and intermediary SDoH were associated with underweight, overweight/obese, and stunting. Understanding the diverse SDoH that predict

For peer review only

malnutrition will be informative to policy generation and the prioritization of programming to address current nutritional challenges.

METHODS

This study used cross-sectional data from late adolescent girls and young women (15−23 y) collected at enrolment within the Matiari emPowerment and Preconception Supplementation (MaPPS) Trial.

Setting and study design

The MaPPS Trial is a two-arm, cluster-randomized, controlled trial that aims to assess the impact of life skills building education and multiple micronutrient supplementation on several nutrition, health, and empowerment-related outcomes in rural Pakistan. The study methodology and data collection tools employed within this population-based effectiveness trial have been previously described.[18] The trial was registered at Clinicaltrials.gov as NCT03287882. The MaPPS Trial aimed to recruit approximately 25,400 non-pregnant adolescent and young women to observe enough pregnancies to detect a 25% relative reduction in low-birth-weight births, assuming prevalence of low birth weight of 30%, icc=0.011, k=0.16, accounting for 10% attrition, and probability of type I and type II errors of 0.05 and 0.20, respectively.

Participant enrolment

Participants were recruited to the MaPPS Trial from the end of June 2017 to July 2018, using a cluster-wise roll-out. The cluster unit was the catchment population of pre-identified health

For peer review only

facilities with their respective outreach community health workers (Lady Health Workers; LHWs) (n=26). Late adolescent girls and young women 15–23 y were identified from a household listing exercise conducted from December 2016 to May 2017. They were approached at their homes by study personnel to further assess their eligibility and ≥1 participant could be enrolled per household. Eligibility criteria included reporting being non-pregnant; physically able to comply with supplementation; not participating in any other nutrition studies; and intending to remain in the study area for the duration of the trial. The purpose and voluntary nature of the trial was explained following confirmation of eligibility, and prior to obtaining informed written consent.

Patient and public involvement

Prior to the start of the household listing exercise, a wide range of activities were undertaken to obtain local approval of the MaPPS Trial. This included sharing information about what was being done within the MaPPS Trial and why with the district education department and health leaders (including the LHW Programme), and leaders from participating villages. The broader community and prospective participants were not invited to comment on the study design, and they were not consulted on the study outcomes or result interpretation for this research.

Data collection

Upon enrolment, a questionnaire was administered to collect information on participant demographics; socioeconomic status; reproductive health; general health, well-being, and nutrition; and empowerment-related variables. Anthropometric (height, weight, middle-upper arm circumference) measurements were collected in duplicate by two trained data collectors using standardized methods.[19] Pre-set allowable differences were employed (height: <1.0 cm; weight:

For peer review only

<0.5 kg), and a third measurement was collected if two measurements exceed this. In all analyses, the average of two acceptable measures was used. The technical error of measurement (TEM), relative TEM (%TEM), and coefficient of reliability (R) were calculated for height and weight.[20] Field monitoring teams additionally conducted regular data quality spot checks.[18] Data collectors were blinded to study arm allocation.

Outcomes of interest

The primary outcome measures were cut-offs for BMI (underweight: <18.5 kg/m^2, normal: 18.5–24.9 kg/m^2, overweight: 25–29.9 kg/m^2, obese: ≥30 kg/m^2) and stunting (height <145 cm).[21] BMI was considered categorically because of the increased health risks when it is too low or high. There are known issues with the adolescent-specific cut-offs for BMI and stunting (10–19 y),[22 23] thus we applied the WHO cut-offs for women of reproductive age (WRA; 15–49 y). This is common in international reporting given the age range overlap. Furthermore, the adult cut-offs are tied to reproductive outcomes and adolescent pregnancies were common in the study population.[17] For completeness, since the WHO Growth Reference is suggested to classify the growth of adolescents up to 19 y (228 completed months), we also applied the WHO igrowup package for Stata (StataCorp, College Station, TX) to adolescents' anthropometric data to determine height-for-age and BMI-for-age z-scores (HAZ and BAZ, respectively).[24]

Explanatory variables

Multiple explanatory variables were hypothesized to underlie the nutritional status outcomes of interest. The selection of underlying SDoH risk factors followed our previously published conceptual framework,[25] which was guided by the UNICEF framework on the causes of malnutrition[26] and the WHO framework on SDoH related to health and well-being.[27] These were

For peer review only

split into two categories: structural (education, occupation, socioeconomic status, and religion) and intermediary (health, well-being, food insecurity, empowerment, and food practices).

Indicators for demographic information, socioeconomic status, and reproductive health were adapted from the Pakistan Demographic Health Survey (PDHS).[17] A variable for wealth quintile was derived using a principal component analysis from factors related to home characteristics and household asset ownership.[17] Other health-related proxies included self-reported health status and mental health (experience of depression, anxiety, and stress-related emotions over the preceding week).[28] Other nutrition-related proxies included household food insecurity,[29] skipping breakfast, and sharing dinner with family.[30] Empowerment proxies included generalized self-efficacy[31] and decision-making autonomy.[17] Self-efficacy scores were categorized into tertiles, corresponding to low, moderate, and high. A variable for decision-making autonomy was derived from reported participation in 5 decisions, adapted from the PDHS (decisions: own food consumption, own health care, household food purchases, household food distribution, household purchases). The scoring of decision-making participation was consistent with the SWPER index for WRA[32] and participants were categorized based on their involvement (all decisions made by family, most decisions made by family, decisions made jointly with family or autonomously).

Statistical analyses

Analyses were performed using Stata, version 15.0 (Stata Corporation). Since the enrolment data were collected prior to the study intervention, data were analyzed cross-sectionally, independent of study arm assignment. To describe the study population, we used descriptive statistics, including means and standard deviations (SD) and proportions for continuous and categorical variables, respectively. Demographic and anthropometric outcomes were further disaggregated

For peer review only

by age group (late adolescent girls: 15–18.9 y; young women: 19–23 y), and a chi-squared test or t-test was used to determine whether there was a difference between proportions and continuous measures, respectively.

We aimed to generate a hierarchical, multivariable model to examine the associations between the SDoH explanatory variables and BMI categories (base: normal BMI [18.5–24.9 kg/m^2]) and stunting (base: height >145 cm), respectively, using our conceptual framework as a guide. The categories for overweight and obese were combined for the BMI variable, given the low prevalence of obesity observed. Because we found that there was a difference in the prevalence of both BMI categories and stunting between late adolescent girls and young women, a separate model was generated for each age group. We hypothesized that there were four levels of SDoH explanatory variables: (1) socioeconomic status: education, occupation, religion, and wealth quintile; (2) household and personal factors: marital status, parity, and household food security; (3) health and well-being: perception of own health, experience of depression-, anxiety-, and stress-like feelings; and (4) actions and practices: self-efficacy, participation in decision making, skipping breakfast, and eating dinner with family. For all analyses, the reference for each SDoH explanatory variable was set as the category hypothesized to present the greatest nutritional vulnerability.[25]

To investigate the association between the SDoH explanatory variables and each primary outcome measure, crude analyses were first conducted using logistic regression. Variables were considered potentially relevant for inclusion in the hierarchical model if the unadjusted P was <0.20.[33] The model building approach described by Victora et al[34] was followed to generate a hierarchical model with 4-levels. Starting with the distal-most level, relevant variables were

For peer review only

entered into the model. At each level, a backward-stepwise model building approach was used. Variables were serially removed to observe any change in the β-coefficients of those variables maintained in the model and find a model that best explained the variability in the data. When the *P* of an explanatory variable was <0.05, it was retained in final model. If the *P* was ≥0.05 and/or the standard error was high, exclusion of the variable was explored. If the β-coefficients of other variables in the model changed by >10%, the excluded variable was returned, otherwise the variable was dropped. The effect estimates in the final model for retained variables correspond to the point of addition to the model. Effect estimates are presented as relative risk ratios (RR) for the variable for BMI categories (three possible outcomes), and odds ratios (OR) for stunting (two possible outcomes). Given the cluster-randomized design of the MaPPS Trial, all models employed cluster robust-standard errors to account for possible correlation between clusters.

RESULTS

Enrolment and exclusion

Of the 32,141 adolescent and young women 15–23 y identified from the household listing exercise, 28,195 (87.7 %) were located, and 25,447 (79.2%) consented to participate in the trial and completed the enrolment questionnaire. Among those enrolled, 14,771 (58.0%) were 15–18.9 y and 10,676 (42.0 %) were 19–23 y, representing 17,044 households.

Participant demographic and reproductive characteristics

At the time of enrolment, participants' mean age (± SD) was 18.8 ± 2.3 y (**Table 1**). No formal education was reported by nearly half of participants, and those with schooling had completed 7.7

For peer review only

± 3.3 y on average. Currently being a student was reported by 13.8% of participants, of which only 61.1% reported regular attendance. Employment outside the home was reported by 38.5% of participants. Common occupations were unskilled and skilled manual labour (17.5% and 20.2% of participants, respectively), although the majority worked within the home (47.5%). Nearly all participants had experienced menarche (25,305 [99.4%]), with the mean age of onset at 13.0 ± 0.9 y (range: 10–21 y). 22.3% of participants were married, with the mean age at marriage of 16.9 ± 1.8 y (range: 13–23 y), and 13.7% had been pregnant. On average, those married were 4.8 ± 4.2 y younger than their husbands.

Table 1. Demographic and anthropometric characteristics among MaPPS Trial participants at enrolment, disaggregated by age group[1]

**Characteristic**	**All participants** (n=25,447)	**15–18.9 y** (n=14,771)	**19–23 y** (n=10,676)	***P***
Age (y)	18.8 ± 2.3	17.1 ± 1.2	21.1 ± 1.2	<0.001
Highest level of education completed				
None	11,384 (44.7)	4,572 (42.0)	2,758 (44.5)	<0.001
Primary	6,063 (23.8)	2,721 (25.0)	1,412 (22.8)	
Secondary or higher	8,000 (31.5)	4,822 (32.6)	3,178 (29.8)	
Employed outside the home	9,620 (38.1)	5,395 (36.6)	4,225 (40.1)	<0.001
Muslim	23,045 (90.6)	13,288 (90.0)	9,757 (91.4)	<0.001
Wealth quintile				
Poorest	4,549 (17.9)	2,666 (18.0)	1,883 (17.6)	<0.001
Poor	4,853 (19.1)	2,881 (19.5)	1,972 (18.5)	
Middle	5,139 (20.2)	3,025 (20.5)	2,114 (19.8)	
Rich	5,360 (21.1)	3,145 (21.3)	2,215 (20.7)	
Richest	5,546 (21.8)	3,054 (20.7)	2,492 (23.3)	
Currently married[2]	5,685 (22.3)	1,418 (9.6)	4,267 (40.0)	<0.001
Age when married husband (y)[3]	16.9 ± 1.8	15.9 ± 1.1	17.2 ± 1.8	<0.001
Has been pregnant[4]	3,478 (13.7)	572 (3.9)	2,906 (27.2)	<0.001
Age at first pregnancy[5]	17.5 ± 1.6	16.4 ± 1.1	17.7 ± 1.6	<0.001
Weight (kg)	47.0 ± 9.4	45.6 ± 8.5	48.9 ± 10.2	<0.001
Height (cm)	152.5 ± 5.7	152.0 ± 5.6	152.7 ± 5.8	<0.001
BMI (kg/m^2)	20.2 ± 3.7	19.7 ± 3.3	20.9 ± 4.0	<0.001
BMI categorization				
Underweight (<18.5 kg/m^2)	9,396 (36.9)	6,105 (41.3)	3,291 (30.8)	<0.001
Normal (18.5-24.9 kg/m^2)	13,463 (52.9)	7,597 (51.4)	5,866 (54.9)	
Overweight (25-29.9 kg/m^2)	2,011 (7.9)	873 (5.9)	1,138 (10.7)	
Obese (≥30 kg/m^2)	577 (2.3)	196 (1.3)	381 (3.6)	
Middle upper arm circumference (cm)	23.7 ± 3.2	23.3 ± 2.9	24.4 ± 3.4	<0.001

[1]Values are n (%) or mean ± SD
[2]158 participants reported no longer being married
[3]Asked of ever-married women (n_{all} = 5834 [3 women did not know]; $n_{15\text{-}18.9y}$=1,457; $n_{19\text{-}23y}$=4,377)

For peer review only

[4]Due to cultural sensitivities, question only asked of ever-married women (n_{all}=3,451 [28 women did not know]; $n_{15-18.9y}$=1,457; n_{19-23y}=4,377)
[5]n_{all}=3,451 (28 women did not know); $n_{15-18.9y}$=567; n_{19-23y}=2,884

Anthropometric characteristics

Regardless of age group, the majority of participants were of 'normal' body weight (52.9%; **Table 1**), although prevalence of underweight was high (36.9%). More late adolescent girls appeared to be underweight, while more young women were overweight/obese (*P*<0.001). Height was <145 cm among 9.2% of participants (late adolescent girls: 10.2%, young women: 7.9%; *P* <0.001), and 35.7% of those stunted were additionally underweight (late adolescent girls: 39.6%, young women: 28.7%; *P* <0.001) and 7.3% overweight/obese (late adolescent girls: 5.4%, young women: 10.7%; *P*=0.006). The TEM for weight and height were 0.22 and 0.18, respectively; %TEM were 0.47% and 0.12%; and the R for each measure was >99%, representing high reliability between the repeat measurements. Among adolescent participants <19 y, HAZ and BAZ were found to be -1.58 ± 0.83 and -0.67 ± 1.13, respectively.

Hierarchical model generation

BMI categories and SDoH risk factors among late adolescent girls

For the hierarchical model looking at BMI categories among late adolescent girls, of the SDoH risk factors identified from the crude analyses, those maintained in the final model included occupation, wealth quintile, marital status, food insecurity, reported perception of health, and self-efficacy (*P* for model: <0.0001; **Table 2**). While a crude association was observed between BMI categories with education level, religion, decision-making, and skipping breakfast, respectively, this was not maintained in the multivariable model (see online supplemental material [Table S1]). Compared to those with a normal BMI, those underweight were more likely

to be from a poorer wealth quintile, unmarried, food insecure, report poorer overall health, and have lower self-efficacy. Among those overweight/obese, compared to those with a normal BMI, someone was less likely to be employed as an unskilled manual labourer and unmarried, and more likely to be from a higher wealth quintile, food secure, and report good health.

BMI categories and SDoH risk factors among young women

Among young women, SDoH risk factors maintained in the final hierarchical model for BMI included occupation, religion, wealth quintile, marital status, reported perception of health, and decision-making autonomy (*P* for model: <0.0001; **Table 3**). While a crude association was observed between BMI categories with food security and self-efficacy, respectively, this was not maintained in the multivariable model (see online supplemental material [Table S2]). Compared to those with a normal BMI, those who were underweight were more likely to be from a poorer wealth quintile, non-Muslim, unmarried, and report poorer overall health and participate in less decision-making. Among those overweight/obese, compared to those with a normal BMI, someone was less likely to be employed as an unskilled manual labourer, but more likely to be from a higher wealth quintile and married.

Stunting among late adolescent girls and young women

The SDoH risk factors maintained in hierarchical models for stunting differed between late adolescent girls and young women (**Table 4**). Among late adolescent girls, the final model included variables from three levels of the hierarchy (level 1: education, wealth quintile; level 2: food security; level 4: self-efficacy, decision-making; *P* for model: <0.0001), while for young women, variables from only the two distal-most levels were maintained (level 1: education, wealth quintile; level 2: parity, food security; *P* for model: <0.0001). The crude association

For peer review only - http://bmjopen.bmj.com/site/about/guidelines.xhtml

observed between stunting and occupation, religion, and skipping breakfast, respectively, was not maintained in either multivariable model (see online supplemental material [Table S3]). Compared to those non-stunted, the odds were lower that stunted adolescents had any education or came from a wealthier or food secure household; they experienced lower self-efficacy and made fewer autonomous decisions. Similarly, stunted young women were found to have a lower odd of education and being from a food secure household, as well as a higher odd of participating in unskilled manual labour; once the other variables were considered in the model, they were more likely not to have been pregnant compared to those non-stunted.

For peer review only

Table 2. Social determinants of health maintained in the hierarchical model of BMI categories (underweight [<18.5 kg/m^2], normal weight [18.5–24.9 kg/m^2; base], and overweight and obese [≥25 kg/m^2]) among late adolescent girls (15–18.9 y) enrolled in the MaPPS Trial at enrolment (n=14,771)[1]

**Possible determinants**	**n (%)**	**BMI categories**							
		**Crude**				**Multivariable adjusted**			
		Underweight	*P*	Overweight/ obese	*P*	Underweight	*P*	Overweight/ obese	*P*
Level 1: Structural factors – socioeconomic status									
Occupation									
Unskilled manual labour	2,622 (17.8)	(reference)	-	(reference)	-	(reference)	-	(reference)	-
Skilled manual labour	2,762 (18.7)	0.85 (0.76 to 0.95)	0.004	2.44 (1.83 to 3.25)	<0.001	0.92 (0.79 to 1.07)	0.26	1.83 (1.42 to 2.36)	<0.001
Within the home	6,351 (43.0)	0.83 (0.75 to 0.91)	<0.001	3.28 (2.54 to 4.25)	<0.001	0.94 (0.83 to 1.07)	0.34	2.03 (1.61 to 2.58)	<0.001
Other[2]	3,036 (20.6)	0.90 (0.81 to 1.00)	0.06	3.26 (2.48 to 4.30)	<0.001	1.14 (0.94 to 1.37)	0.18	1.44 (1.09 to 1.92)	0.01
Wealth quintile									
Poorest	2,666 (18.0)	(reference)	-	(reference)	-	(reference)	-	(reference)	-
Poor	2,881 (19.5)	0.84 (0.76 to 0.94)	0.002	1.47 (1.07 to 2.01)	0.001	0.84 (0.74 to 0.95)	0.01	1.34 (1.07 to 1.67)	0.01
Middle	3,025 (20.5)	0.79 (0.71 to 0.88)	<0.001	2.36 (1.77 to 3.15)	<0.001	0.78 (0.68 to 0.89)	<0.001	2.07 (1.63 to 2.63)	<0.001
Rich	3,145 (21.3)	0.71 (0.64 to 0.79)	<0.001	3.42 (2.59 to 4.51)	<0.001	0.69 (0.58 to 0.82)	<0.001	2.94 (2.32 to 3.74)	<0.001
Richest	3,054 (20.7)	0.69 (0.62 to 0.77)	<0.001	5.18 (3.96 to 6.79)	<0.001	0.65 (0.56 to 0.74)	<0.001	4.65 (3.49 to 6.20)	<0.001
Level 2: Intermediary factors - household and personal characteristics									
Marital status									
Married	1,418 (9.6)	(reference)		(reference)		(reference)	-	(reference)	-
Unmarried	13,353 (90.4)	1.38 (1.23 to 1.56)	<0.001	0.80 (0.66 to 0.97)	<0.03	1.42 (1.28 to 1.59)	<0.001	0.71 (0.59 to 0.85)	<0.001
Household food security status									
Food insecure	3,861 (26.1)	(reference)		(reference)		(reference)	-	(reference)	-
Food secure	10,910 (73.9)	0.83 (0.77 to 0.90)	<0.001	1.92 (1.61 to 2.28)	<0.001	0.91 (0.83 to 0.99)	0.04	1.24 (1.03 to 1.51)	0.03
Level 3: Intermediary factors - health and well-being characteristics									
Perception of own health									
Poor or fair	1,533 (10.4)	(reference)		(reference)		(reference)	-	(reference)	-
Good	9,731 (65.9)	0.82 (0.74 to 0.92)	0.001	1.23 (0.97 to 1.57)	0.09	0.83 (0.70 to 0.97)	0.03	1.23 (0.89 to 1.68)	0.21
Excellent	3,507 (23.7)	0.71 (0.63 to 0.80)	<0.001	1.40 (1.08 to 1.81)	0.01	0.74 (0.62 to 0.88)	0.001	1.22 (0.93 to 1.60)	0.16
Level 4: Intermediary factors - actions and practices-related characteristics									
Self-efficacy									
Low	5,695 (38.6)	(reference)	-	(reference)	-	(reference)	-	(reference)	-
Moderate	7,161 (48.5)	0.85 (0.79 to 0.92)	<0.001	1.27 (1.10 to 1.46)	0.001	0.91 (0.85 to 0.97)	<0.001	1.09 (0.93 to 1.27)	0.29
High	1,915 (13.0)	0.75 (0.67 to 0.83)	<0.001	1.12 (0.91 to 1.37)	0.29	0.82 (0.72 to 0.92)	<0.001	0.91 (0.73 to 1.14)	0.41

[1]Values are n (%) or RR (95% CI)

[2]Other category included those who identified themselves as students (2,988[20.2%]) or professionals (48[0.3%])

For peer review only

Table 3. Social determinants of health maintained in the hierarchical model of BMI categories (underweight [<18.5 kg/m^2], normal weight [18.5–24.9 kg/m^2; base], and overweight and obese [≥25 kg/m^2]) among young women (19–23 y) enrolled in the MaPPS Trial at enrolment (n=10,676)

Possible determinants	n (%)	BMI categories: Crude: Underweight	*P*	Crude: Overweight/ obese	*P*	Multivariable adjusted: Underweight	*P*	Multivariable adjusted: Overweight/ obese	*P*
Level 1: Structural factors – socioeconomic status									
Occupation									
Unskilled manual labour	1,837 (17.2)	(reference)	-	(reference)	-	(reference)	-	(reference)	-
Skilled manual labour	2,372 (22.2)	0.81 (0.71 to 0.93)	0.002	2.77 (2.17 to 3.55)	<0.001	0.95 (0.83 to 1.09)	0.48	1.91 (1.48 to 2.48)	<0.001
Within the home	5,779 (54.1)	0.75 (0.67 to 0.84)	<0.001	3.52 (2.81 to 4.42)	<0.001	0.93 (0.83 to 1.05)	0.25	2.07 (1.61 to 2.67)	<0.001
Other[2]	688 (6.4)	0.76 (0.62 to 0.93)	0.007	3.80 (2.83 to 5.11)	<0.001	1.11 (0.83 to 1.49)	0.47	1.64 (1.19 to 2.26)	0.002
Religion									
Non-Muslim	919 (8.6)	(reference)	-	(reference)	-	(reference)	-	(reference)	-
Muslim	9,757 (91.4)	0.64 (0.56 to 0.74)	<0.001	1.74 (1.35 to 2.25)	<0.001	0.76 (0.61 to 0.93)	0.01	0.98 (0.71 to 1.36)	0.92
Wealth quintile									
Poorest	1,883 (17.6)	(reference)	-	(reference)	-	(reference)	-	(reference)	-
Poor	1,972 (18.5)	0.81 (0.71 to 0.92)	0.002	1.98 (1.48 to 2.64)	<0.001	0.87 (0.74 to 1.02)	0.09	1.81 (1.36 to 2.43)	<0.001
Middle	2,114 (19.8)	0.73 (0.64 to 0.83)	<0.001	3.31 (2.52 to 4.34)	<0.001	0.79 (0.70 to 0.89)	<0.001	2.84 (2.02 to 4.00)	<0.001
Rich	2,215 (20.7)	0.62 (0.54 to 0.71)	<0.001	4.68 (3.60 to 6.09)	<0.001	0.68 (0.59 to 0.78)	<0.001	3.88 (2.85 to 5.28)	<0.001
Richest	2,492 (23.3)	0.55 (0.48 to 0.63)	<0.001	6.36 (4.92 to 8.22)	<0.001	0.59 (0.48 to 0.72)	<0.001	5.24 (3.93 to 6.98)	<0.001
Level 2: Intermediary factors - household and personal characteristics									
Marital status									
Married	4,267 (40.0)	(reference)	-	(reference)	-	(reference)	-	(reference)	-
Unmarried	6,409 (60.0)	1.14 (1.04 to 1.24)	0.004	0.90 (0.81 to 1.01)	0.08	1.25 (1.11 to 1.42)	<0.001	0.77 (0.69 to 0.85)	<0.001
Perception of own health									
Poor or fair	1,275 (11.9)	(reference)	-	(reference)	-	(reference)	-	(reference)	-
Good	6,828 (64.0)	0.78 (0.68 to 0.89)	<0.001	1.18 (0.97 to 1.44)	0.09	0.78 (0.69 to 0.88)	<0.001	1.16 (0.94 to 1.44)	0.18
Excellent	2,573 (24.1)	0.75 (0.64 to 0.87)	<0.001	1.35 (1.09 to 1.67)	0.006	0.78 (0.69 to 0.89)	<0.001	1.16 (0.89 to 1.54)	0.27
Decision-making autonomy									
All decisions made by family	4,365 (40.9)	(reference)	-	(reference)	-	(reference)	-	(reference)	-
Most decisions made by family	3,630 (34.0)	0.91 (0.83 to 1.01)	0.08	1.08 (0.95 to 1.24)	0.23	0.92 (0.85 to 0.99)	0.03	1.08 (0.93 to 1.27)	0.31
Decisions made jointly with family or autonomously	2,681 (25.1)	0.85 (0.76 to 0.94)	0.003	1.19 (1.04 to 1.37)	0.01	0.85 (0.77 to 0.95)	0.003	1.24 (1.06 to 1.45)	0.01

[1]Values are n (%) or RR (95% CI)

[2]Other category included those who identified themselves as a student (526[4.9%]) or professional (688[6.4%])

Table 4. Comparison of social determinants of health in hierarchical model of stunting (height <145 cm) among late adolescent girls (n=14,771) and adult women (n=10,676) enrolled in the MaPPS Trial at enrolment

Possible determinants	Stunting among late adolescent girls				Stunting among young women			
	Crude		Multivariable adjusted		Crude		Multivariable adjusted	
	OR (95% CI)	*P*	OR (95% CI)	*P*	OR (95% CI)	*P*	OR (95% CI)	*P*
Level 1: Structural factors – socioeconomic status								
Highest level of education								
None	(reference)	-	(reference)	-	(reference)	-	(reference)	-
Primary	0.65 (0.57 to 0.74)	<0.001	0.76 (0.62 to 0.94)	0.01	0.62 (0.51 to 0.74)	<0.001	0.76 (0.63 to 0.93)	0.006
Secondary or higher	0.33 (0.29 to 0.38)	<0.001	0.48 (0.39 to 0.59)	<0.001	0.51 (0.43 to 0.61)	<0.001	0.79 (0.66 to 0.95)	0.01
Wealth quintile								
Poorest	(reference)	-	(reference)	-	(reference)	-	(reference)	-
Poor	0.83 (0.72 to 0.97)	0.02	0.91 (0.77 to 1.08)	0.29	0.88 (0.72 to 1.08)	0.22	0.92 (0.73 to 1.17)	0.52
Middle	0.67 (0.57 to 0.78)	<0.001	0.79 (0.65 to 0.96)	0.02	0.65 (0.52 to 0.80)	<0.001	0.70 (0.59 to 0.84)	<0.001
Rich	0.46 (0.39 to 0.55)	<0.001	0.63 (0.49 to 0.81)	<0.001	0.48 (0.38 to 0.60)	<0.001	0.54 (0.40 to 0.74)	<0.001
Richest	0.26 (0.21 to 0.32)	<0.001	0.42 (0.35 to 0.51)	<0.001	0.34 (0.27 to 0.43)	<0.001	0.41 (0.29 to 0.57)	<0.001
Level 2: Intermediary factors - household and personal characteristics								
Ever been pregnant								
Yes	(reference)	-	-	-	(reference)	-	(reference)	-
No	0.97 (0.74 to 1.27)	0.81	-	-	1.13 (0.96 to 1.32)	0.15	1.27 (1.10 to 1.47)	0.001
Household food security status								
Food insecure	(reference)	-	(reference)	-	(reference)	-	(reference)	-
Food secure	0.58 (0.51 to 0.64)	<0.001	0.77 (0.66 to 0.90)	0.001	0.57 (0.49 to 0.66)	<0.001	0.73 (0.59 to 0.90)	0.003
Level 4: Intermediary factors - actions and practices-related characteristics								
Self-efficacy								
Low	(reference)	-	(reference)	-	(reference)	-	-	-
Moderate	0.62 (0.56 to 0.70)	<0.001	0.74 (0.64 to 0.84)	<0.001	0.83 (0.71 to 0.98)	0.03	-	-
High	0.59 (0.50 to 0.71)	<0.001	0.74 (0.62 to 0.87)	<0.001	0.78 (0.63 to 0.97)	0.03	-	-
Decision-making autonomy								
All decisions made by family	(reference)	-	(reference)	-	(reference)	-	-	-
Most decisions made by family	0.83 (0.74 to 0.94)	0.003	0.88 (0.76 to 1.01)	0.08	0.99 (0.84 to 1.16)	0.90	-	-
Decisions made jointly with family or autonomously	0.74 (0.62 to 0.89)	0.001	0.76 (0.62 to 0.92)	0.01	0.85 (0.71 to 1.02)	0.09	-	-

For peer review only

DISCUSSION

Using enrolment data from a large trial conducted in rural Pakistan, we found high levels of undernutrition among late adolescent and young women 15–23 y using anthropometric indicators. Our study offers the most comprehensive analysis of the determinants of malnutrition among late adolescent girls and young women in rural Pakistan. Across the multivariable models for acute and chronic measures of undernutrition, those who were impoverished were at increased risk regardless of age group. This suggests that structural factors play an important, underlying role in female nutrition in this setting. That an emerging burden of overweight/obesity was also observed in this rural population indicates that interventions are needed to address both ends of the malnutrition spectrum. To the best of our knowledge, this is the largest survey of a cohort that includes both unmarried and married Pakistani late adolescent girls and young women.

Pakistan is known to have a high burden of malnutrition, with a long term impact on human capital and costing an estimated 7.6 billion USD/year.[35] We found the mean BMI among study participants was lower than the PDHS national average (20.2 kg/m^2 versus 25.7 kg/m^2).[17] This likely reflects that the PDHS only surveyed ever-married WRA (15–49 y). In the recently completed 2018 Pakistan National Nutrition Survey, 22.6%, 19.8%, and 10.2% of WRA in Sindh province were found to be underweight, overweight, and obese, respectively.[36] Notably, both the PDHS and National Nutrition Survey include urban settings, where the risk of overweight and obesity is higher.[37]

Of the limited adolescent nutrition studies available globally, wealth has consistently been found to be a determinant of undernutrition in multiple settings.[38-42] Regardless of whether someone

For peer review only

was an adolescent or young women, we found that at least one structural SDoH variable tied to poverty was associated with an increased risk of undernutrition outcomes. Notably, food insecurity was maintained in several models. At least one variable considered a proxy for empowerment was maintained in each adolescent model, with greater empowerment being associated with an improvement in nutritional status. Considered a blend of resources (materials that allow someone to make her goals a reality), agency (the ability to define and act upon someone's goals), and achievement (obtaining a desired goal), empowerment enables someone to make decisions to take charge of her own life and affect her personal circumstances.[43] Empowered women are widely recognized to be better able to promote their own health and well-being; whereas, lacking empowerment is suggested to present a further barriers to achieving optimal nutritional status.[3]

We had hypothesized that married late adolescent and young women might be at greater risk for undernutrition because early marriage has been suggested to increase nutritional vulnerability;[44] however, in the hierarchical models for both adolescent and young women, being married was associated with increased likelihood of being in a higher BMI category compared to those unmarried. This it is likely attributable to an increase in status within the household, as married participants contributed more to food-related decision-making (data not shown). An association between marital status and stunting, a chronic measure of poor nutrition across the lifespan, was not observed.

There is a recognized need in Pakistan to focus on the health and nutrition of adolescent girls,[45 46] and it is well documented that preconception health and well-being are important to the individual, as well as to her future offspring.[47] To address inequalities due to poverty, the use of

For peer review only

social safety nets could function as an important resource. As food insecurity prevalence was notable, provision of cash transfers, for example, could lend to the purchase of more nutritious food, although we are not certain at this time of the exact effect that this might have on an adolescent or young women's actual intake. However, economic status alone does not confer a healthy BMI.[42] We would note that there will need to be a concurrent emphasis on maintaining a healthy BMI, given that the prevalence of overweight/obesity in Pakistan has increased over time in both urban and rural settings, the existing double burden of malnutrition, and ongoing nutrition transition. To address structural and empowerment-related barriers, increased and coordinated efforts between the public health system and the education sector could have potential. The Government of Pakistan has recently released its nutritional strategy and operational plan for targeting adolescent malnutrition, which will be important to implementing legislation on adolescent nutrition.[48]

Limitations of this study include that our catchment area was restricted to the areas of Matiari District covered by the LHW Programme. Furthermore, our findings are specific to the context in rural Pakistan, and findings may not reflect settings with different cultural norms or burdens of undernutrition, as seen in Africa and South America.[49] While BMI is a globally accepted index of nutritional status, the relationship between BMI and body composition can be controversial, as BMI does not account for excess weight due to adiposity versus muscularity, edema, or bone mass.[6] It would have been informative to have had additional measures of body composition given its influence on health. We additionally acknowledge that the adolescent period does commence at 10 y, although given the aims of the MaPPS Trial we did not enrol adolescent girls 10–14 y. The larger study focused on adolescent and young women, not adolescent boys or young men; yet, males are also important given their earning potential and contribution to the

For peer review only

economy.[46] A further similar evaluation of this group would be informative, and aid understanding gendered cultural norms. Although the assessment of macro-level SDoH (e.g., governance, policies) was beyond the scope of this assessment, the importance of structural SDoH to all models suggests that underlying factors that create divisions within society are precarious. In their cross-sectional assessment of the factors affecting thinness among adolescent girls 10–19 y in low and middle income countries, Candler et al[38] found that macroeconomic factors contribute to the genesis of adolescent thinness, and that reducing adolescent thinness should include addressing national food security and economic development.

CONCLUSIONS

Given the findings of this study, and the concerning status of malnutrition in Pakistan, commitment to improving the nutrition of adolescent and young women in Pakistan is critical and should include those unmarried. Reaching these individuals is important not only because of their reproductive potential, but also given their right to optimal nutrition and well-being.[50] To target undernutrition, particularly, there is a need for both direct and indirect nutrition and empowerment interventions that are integrated and multi-sectoral,[51] as highlighted in the recent national adolescent nutrition policy recommendations.[48] An ideal program for adolescent and young women should be inclusive and accommodating, reach those who are most vulnerable, and account for cultural- and gender-relevant factors.[52]

For peer review only

Acknowledgements: The authors wish to acknowledge the participants in the MaPPS Trial and the field research team. We are grateful for the ongoing data-related assistance and support provided by Rasool Bux, Arjumand Rizvi, and the members of the Data Management Unit at Aga Khan University.

Contributions: JBB and ZAB designed research; YW and SBS conducted research; IA and AH performed data management; JBB analyzed data; JBB wrote first manuscript draft; ZAB, YW, SBS, IA, and AH provided manuscript feedback; ZAB has primary responsibility for final content. All authors reviewed this manuscript and approved the final version.

Funding: The MaPPS Trial was supported by the Bill and Melinda Gates Foundation (grant number: OPP1148892) and the World Food Programme (grant number: HQ15NF493 - CTR). JBB received research scholarships from the Vanier Canada Graduate Scholarship; Hospital for Sick Children Foundation Student Scholarship Program; and the Province of Ontario.

Competing interests: None declared.

Ethical approval: Ethical review for this study was obtained from the Aga Khan University Ethics Review Committee (Protocol #4324-Ped-ERC-16) and the Hospital for Sick Children Research Ethics Board (Protocol #1000054682).

Data availability statement: The anonymized, individual-level data from this cross-sectional assessment, situated within a clinical trial, will be made available upon reasonable request from the corresponding author.

For peer review only

REFERENCES

1. World Health Organization. Guideline: implementing effective actions for improving adolescent nutrition. Geneva: World Health Organization, 2018.

2. Sawyer SM, Afifi RA, Bearinger LH, et al. Adolescence: a foundation for future health. *Lancet* 2012;379(9826):1630-40.

3. Christian P, Smith ER. Adolescent Undernutrition: Global Burden, Physiology, and Nutritional Risks. *Ann Nutr Metab* 2018;72(4):316-28.

4. Norris SA, Frongillo EA, Black MM, et al. Nutrition in adolescent growth and development. *Lancet* 2022;399(10320):172-84. doi: 10.1016/s0140-6736(21)01590-7

5. Aguayo VM, Paintal K. Nutrition in adolescent girls in South Asia. *BMJ (Clinical research ed)* 2017;357:j1309. doi: 10.1136/bmj.j1309 [published Online First: 2017/04/13]

6. Gibson R. Principles of Nutritional Assessment. New York: Oxford University Press 2005.

7. Black RE, Victora CG, Walker SP, et al. Maternal and child undernutrition and overweight in low-income and middle-income countries. *Lancet* 2013;382(9890):427-51.

8. Twig G, Yaniv G, Levine H, et al. Body mass index in 2.3 million adolescents and cardiovascular death in adulthood. *N Engl J Med* 2016;374:2430–40.

9. Saydah S, Bullard KM, Imperatore G, et al. Cardiometabolic risk factors among US adolescents and young adults and risk of early mortality. *Pediatrics* 2013;131:e679-86. .

10. Viner RM, Ozer EM, Denny S, et al. Adolescence and the social determinants of health. *Lancet* 2012;379(9826):1641-52.

For peer review only

11. Commission on Social Determinants of Health. Closing the gap in a generation: health equity through action on the social determinants of health. Geneva: World Health Organization, 2008.

12. Fatusi AO, Bello B. Social Determinants of Health in Adolescent and Young Women's Health and Nutrition: Current Evidence. In: Bhutta ZA, Makarides M, Prentice AM, eds. Health and Nutrition in Adolescents and Young Women: Preparing for the Next Generation. Basel: Nestle Nutr Inst Workshop Ser 2015:61-69.

13. United Nations. Transforming Our World: The 2030 Agenda for Sustainable Development. New York: United Nations 2015.

14. Christian P, Smith E. Adolescent Undernutrition: Global Burden, Physiology, and Nutritional Risks. *Annals of nutrition & metabolism* 2018;72(4):316-28.

15. Bhutta Z, Lassi Z, Bergeron G, et al. Delivering an action agenda for nutrition interventions addressing adolescent girls and young women: priorities for implementation and research. *Annals of the New York Academy of Sciences* 2017;1393(1):61-71.

16. Ruel MT, Alderman H. Nutrition-sensitive interventions and programmes: how can they help to accelerate progress in improving maternal and child nutrition? *Lancet* 2013;382(9891):536-51. doi: 10.1016/s0140-6736(13)60843-0 [published Online First: 2013/06/12]

17. National Institute of Population Studies (NIPS) [Pakistan] and ICF. Pakistan Demographic and Health Survey 2017-18. Islamabad, Pakistan, and Rockville, Maryland, USA: NIPS and ICF, 2019.

18. Baxter JB, Wasan Y, Soofi SB, et al. Effect of life skills building education and micronutrient supplements provided from preconception versus the standard of care on

For peer review only

low birth weight births among adolescent and young Pakistani women (15-24 years): a prospective, population-based cluster-randomized trial. *Reprod Health* 2018;15(1):104.

19. Centers for Disease Control and Prevention. National Health and Nutrition Examination Survey: anthropometry procedures manual. Hyattsville, MD: National Center for Health Statistics, Centers for Disease Control and Prevention, 2009.

20. Ulijaszek SJ, Kerr, D.A.,. Anthropometric measurement error and the assessment of nutritional status. *Br J Nutr* 1999;82:165–77.

21. World Health Organization. 2018 Global Reference List of 100 Core Health Indicators (plus health-related SDGs). Geneva: World Health Organization, 2018.

22. Baxter JB, Kortenaar JL, Wasan Y, et al. Age-Based Anthropometric Cutoffs Provide Inconsistent Estimates of Undernutrition: Findings from a Cross-Sectional Assessment of Late-Adolescent and Young Women in Rural Pakistan. *Curr Dev Nutr* 2021;5(11):nzab130. doi: 10.1093/cdn/nzab130 [published Online First: 2021/12/14]

23. Tumilowicz A, Beal T, Neufeld LM, et al. Perspective: Challenges in Use of Adolescent Anthropometry for Understanding the Burden of Malnutrition. *Adv Nutr* 2019;10(4):563-75.

24. de Onis M, Onyango AW, Borghi E, et al. Development of a WHO growth reference for school-aged children and adolescents. *Bull World Health Organ* 2007;85:660–67.

25. Baxter JB, Wasan Y, Hussain A, et al. Characterizing Micronutrient Status and Risk Factors among Late Adolescent and Young Women in Rural Pakistan: A Cross-Sectional Assessment of the MaPPS Trial. *Nutrients* 2021;13:1237.

26. UNICEF. The State of the World's Children [Internet]. New York: Oxford University Press 1998.

For peer review only

27. Solar O, Irwin A. A conceptual framework for action on the social determinants of health. Social Determinants of Health Discussion Paper 2 (Policy and Practice). Geneva: World Health Organization, 2010.

28. Lovibond SH, Lovibond PF. Manual for the Depression Anxiety Stress Scales. Sydney: Psychology Foundation, 1995.

29. Coates J, Swindale A, Bilinsky P. Household food insecurity access scale (HFIAS) for measurement of household food access: Indicator guide (v. 3). Washington: Food and Nutrition Technical Assistance Project, Academy for Educational Development, 2007.

30. Currie C, Inchley J, Molcho M, et al. Health Behaviour in School-aged Children (HBSC) Study Protocol: Background, Methodology, and Mandatory items for the 2013/14 Survey. St. Andrews: CAHRU, 2014.

31. Schwarzer R, Jerusalem M. Generalized self-efficacy scale. In: Weinman J WS, Johnston M, ed. Measures in health psychology: a user's portfolio Causal and control beliefs. Windsor: NFER-NELSON 1995:35-37.

32. Ewerling F, Lynch JW, Victora CG, et al. The SWPER index for women's empowerment in Africa: development and validation of an index based on survey data. *Lancet Glob Health* 2017;5(9):e916-e23.

33. Hosmer DW, Lemeshow S. Applied Logistic Regression. 2nd ed. New York: John Wiley 2000.

34. Victora CG, Huttly SR, Fuchs SC, et al. The role of conceptual frameworks in epidemiological analysis: a hierarchical approach. *Int J Epidemiol* 1997;26:224-27.

35. World Food Programme. The Economic Consequences of Undernutrition in Pakistan: An Assessment of Losses. Islamabad: World Food Programme Pakistan, 2017.

For peer review only

36. UNICEF Pakistan. National Nutrition Survey 2018: Key Findings Report. Islamabad: UNICEF Pakistan 2018.

37. Janjua NZ, Mahmood B, Bhatti JA, et al. Association of household and community socioeconomic position and urbanicity with underweight and overweight among women in Pakistan. *PLoS One* 2015;10(4):e0122314.

38. Candler T, Costa S, Heys M, et al. Prevalence of Thinness in Adolescent Girls in Low- and Middle-Income Countries and Associations With Wealth, Food Security, and Inequality. . *J Adolesc Health* 2017;60(4):447-54.

39. Harding KL, Aguayo VM, Webb P. Trends and Correlates of Overweight among Pre-School Age Children, Adolescent Girls, and Adult Women in South Asia: An Analysis of Data from Twelve National Surveys in Six Countries over Twenty Years. *Nutrients* 2019;11(8):1899.

40. Leroy JL, Ruel M, Sununtnasuk C, et al. Understanding the determinants of adolescent nutrition in Bangladesh. *Ann N Y Acad Sci* 2018

41. Radhika MS, Swetha B, Kumar NB, et al. Dietary and nondietary determinants of nutritional status among adolescent girls and adult women in India. *Ann N Y Acad Sci* 2018;1416(1):5-17.

42. Young MF, Nguyen P, Tran LM, et al. A Double Edged Sword? Improvements in Economic Conditions over a Decade in India Led to Declines in Undernutrition as Well as Increases in Overweight among Adolescents and Women. *The Journal of nutrition* 2020;150(2):364-72. doi: 10.1093/jn/nxz251 [published Online First: 2019/10/17]

43. Kabeer N. Resources, agency, achievements: reflections on the measurement of women's empowerment. *Dev Change* 1999;30:435-64.

For peer review only

44. Marphatia AA, Ambale GS, Reid AM. Women's Marriage Age Matters for Public Health: A Review of the Broader Health and Social Implications in South Asia. *Frontiers in public health* 2017;5:269. doi: 10.3389/fpubh.2017.00269 [published Online First: 2017/11/03]

45. Government of Pakistan. National Vision 2016-2025 for Coordinated Priority Actions to Address Challenges of Reproductive, Maternal, Newborn, Child, Adolescent Health and Nutrition. Islamabad: Government of Pakistan, 2015.

46. Government of Pakistan, World Health Organization. Adolescent Nutrition and Supplementation Guidelines for Pakistan. Islamabad: Government of Pakistan, 2019.

47. Dean SV, Lassi ZS, Imam AM, et al. Preconception care: nutritional risks and interventions. *Reprod Health* 2014;11(Suppl 3):S3.

48. Government of Pakistan. Pakistan Adolescent Nutrition Strategy and Operations Plan. Karachi: Government of Pakistan 2020.

49. Akseer N, Al-Gashm S, Mehta S, et al. Global and regional trends in the nutritional status of young people: a critical and neglected age group. *Ann N Y Acad Sci* 2017;1393(1):3-20.

50. Fox EL, Davis C, Downs SM, et al. Who is the Woman in Women's Nutrition? A Narrative Review of Evidence and Actions to Support Women's Nutrition throughout Life. *Current Developments in Nutrition* 2018;3(1) doi: 10.1093/cdn/nzy076

51. Keats E, Das JK, Salam RA, et al. Effective interventions to address maternal and child malnutrition: an update of the evidence. *The Lancet Child & adolescent health* 2021 doi: 10.1016/s2352-4642(20)30274-1

52. Hargreaves D, Mates E, Menon P, et al. Strategies and interventions for healthy adolescent growth, nutrition, and development. *Lancet* 2022;399(10320):198-210. doi: 10.1016/s0140-6736(21)01593-2

For peer review only

Supplementary Material

Table S1. Comparison of social determinants of health with BMI categories (underweight [<18.5 kg/m^2], normal weight [18.5–24.9 kg/m^2; base], and overweight and obese [≥25 kg/m^2]) among late adolescent girls (15–18.9 y) enrolled in the MaPPS Trial at enrolment (n=14,771)[1]

**Possible determinants**	**n (%)**	**BMI categories**							
		**Crude**				**Multivariable adjusted**			
		Underweight	*P*	Overweight/ obese	*P*	Underweight	*P*	Overweight/ obese	*P*
Level 1: Structural factors – socioeconomic status									
Highest level of education									
None	6,297 (42.6)	(reference)	-	(reference)	-	-	-	-	-
Primary	3,652 (24.7)	0.96 (0.89 to 1.05)	0.39	1.46 (1.23 to 1.74)	<0.001	-	-	-	-
Secondary or higher	4,822 (32.6)	0.86 (0.80 to 0.93)	<0.001	2.05 (1.76 to 2.38)	<0.001	-	-	-	-
Occupation									
Unskilled manual labour	2,622 (17.8)	(reference)	-	(reference)	-	(reference)	-	(reference)	-
Skilled manual labour	2,762 (18.7)	0.85 (0.76 to 0.95)	0.004	2.44 (1.83 to 3.25)	<0.001	0.92 (0.79 to 1.07)	0.26	1.83 (1.42 to 2.36)	<0.001
Within the home	6,351 (43.0)	0.83 (0.75 to 0.91)	<0.001	3.28 (2.54 to 4.25)	<0.001	0.94 (0.83 to 1.07)	0.34	2.03 (1.61 to 2.58)	<0.001
Other[2]	3,036 (20.6)	0.90 (0.81 to 1.00)	0.06	3.26 (2.48 to 4.30)	<0.001	1.14 (0.94 to 1.37)	0.18	1.44 (1.09 to 1.92)	0.01
Religion									
Non-Muslim	1,483 (10.0)	(reference)	-	(reference)	-	-	-	-	-
Muslim	13,288 (90.0)	0.79 (0.71 to 0.88)	<0.001	1.96 (1.48 to 2.61)	<0.001	-	-	-	-
Wealth quintile									
Poorest	2,666 (18.0)	(reference)	-	(reference)	-	(reference)	-	(reference)	-
Poor	2,881 (19.5)	0.84 (0.76 to 0.94)	0.002	1.47 (1.07 to 2.01)	0.001	0.84 (0.74 to 0.95)	0.01	1.34 (1.07 to 1.67)	0.01
Middle	3,025 (20.5)	0.79 (0.71 to 0.88)	<0.001	2.36 (1.77 to 3.15)	<0.001	0.78 (0.68 to 0.89)	<0.001	2.07 (1.63 to 2.63)	<0.001
Rich	3,145 (21.3)	0.71 (0.64 to 0.79)	<0.001	3.42 (2.59 to 4.51)	<0.001	0.69 (0.58 to 0.82)	<0.001	2.94 (2.32 to 3.74)	<0.001
Richest	3,054 (20.7)	0.69 (0.62 to 0.77)	<0.001	5.18 (3.96 to 6.79)	<0.001	0.65 (0.56 to 0.74)	<0.001	4.65 (3.49 to 6.20)	<0.001
Level 2: Intermediary factors - household and personal characteristics									
Marital status									
Married	1,418 (9.6)	(reference)		(reference)		(reference)	-	(reference)	-
Unmarried	13,353 (90.4)	1.38 (1.23 to 1.56)	<0.001	0.80 (0.66 to 0.97)	<0.03	1.42 (1.28 to 1.59)	<0.001	0.71 (0.59 to 0.85)	<0.001
Ever been pregnant									
Yes	572 (3.9)	(reference)	-	(reference)	-	-	-	-	-
No	14,199 (96.1)	1.14 (0.96 to 1.36)	0.14	0.80 (0.59 to 1.08)	0.14	-	-	-	-
Household food security status									
Food insecure	3,861 (26.1)	(reference)		(reference)		(reference)	-	(reference)	-
Food secure	10,910 (73.9)	0.83 (0.77 to 0.90)	<0.001	1.92 (1.61 to 2.28)	<0.001	0.91 (0.83 to 0.99)	0.04	1.24 (1.03 to 1.51)	0.03
Level 3: Intermediary factors - health and well-being characteristics									
Perception of own health									
Poor or fair	1,533 (10.4)	(reference)		(reference)		(reference)	-	(reference)	-

For peer review only

Possible determinants	n (%)	BMI categories							
		Crude				Multivariable adjusted			
		Underweight	*P*	Overweight/ obese	*P*	Underweight	*P*	Overweight/ obese	*P*
Good	9,731 (65.9)	0.82 (0.74 to 0.92)	0.001	1.23 (0.97 to 1.57)	0.09	0.83 (0.70 to 0.97)	0.03	1.23 (0.89 to 1.68)	0.21
Excellent	3,507 (23.7)	0.71 (0.63 to 0.80)	<0.001	1.40 (1.08 to 1.81)	0.01	0.74 (0.62 to 0.88)	0.001	1.22 (0.93 to 1.60)	0.16
Experience of depression-like feelings									
Severe or extremely severe	687 (4.7)	(reference)	-	(reference)	-	-	-	-	-
Moderate	1,545 (10.5)	0.93 (0.78 to 1.12)	0.47	1.16 (0.78 to 1.71)	0.46	-	-	-	-
Mild	1,236 (8.4)	0.94 (0.78 to 1.14)	0.53	1.15 (0.76 to 1.72)	0.51	-	-	-	-
None	11,303 (76.5)	0.78 (0.67 to 0.92)	0.002	1.20 (0.85 to 1.68)	0.30	-	-	-	-
Experience of anxiety-like feelings									
Severe or extremely severe	1,893 (12.8)	(reference)	-	(reference)	-	-	-	-	-
Moderate	2,124 (14.4)	1.02 (0.90 to 1.16)	0.78	0.96 (0.74 to 1.25)	0.77	-	-	-	-
Mild	952 (6.4)	0.85 (0.72 to 1.00)	0.05	1.06 (0.77 to 1.44)	0.73	-	-	-	-
None	9,802 (66.4)	0.79 (0.72 to 0.88)	<0.001	1.04 (0.85 to 1.27)	0.72	-	-	-	-
Experience of stress-like feelings									
Severe or extremely severe	491 (3.3)	(reference)	-	(reference)	-	-	-	-	-
Moderate	778 (5.3)	0.94 (0.75 to 1.19)	0.61	0.88 (0.54 to 1.41)	0.59	-	-	-	-
Mild	885 (6.0)	0.91 (0.72 to 1.14)	0.40	1.07 (0.68 to 1.68)	0.78	-	-	-	-
None	12,617 (85.4)	0.80 (0.67 to 0.97)	0.02	1.03 (0.70 to 1.49)	0.89	-	-	-	-
Level 4: Intermediary factors - actions and practices-related characteristics									
Self-efficacy									
Low	5,695 (38.6)	(reference)	-	(reference)	-	(reference)	-	(reference)	-
Moderate	7,161 (48.5)	0.85 (0.79 to 0.92)	<0.001	1.27 (1.10 to 1.46)	0.001	0.91 (0.85 to 0.97)	<0.001	1.09 (0.93 to 1.27)	0.29
High	1,915 (13.0)	0.75 (0.67 to 0.83)	<0.001	1.12 (0.91 to 1.37)	0.29	0.82 (0.72 to 0.92)	<0.001	0.91 (0.73 to 1.14)	0.41
Decision-making autonomy									
All decisions made by family	8,244 (55.8)	(reference)	-	(reference)	-	-	-	-	-
Most decisions made by family	4,702 (31.8)	0.9 (0.84 to 0.97)	0.008	1.18 (1.03 to 1.36)	0.02	-	-	-	-
Decisions made jointly with family or autonomously	1,825 (12.4)	0.84 (0.76 to 0.94)	0.002	1.32 (1.10 to 1.60)	0.004	-	-	-	-
Skipping breakfast									
Skips breakfast	4,479 (30.3)	(reference)	-	(reference)	-	-	-	-	-
Eats breakfast	10,292 (69.7)	0.99 (0.92 to 1.07)	0.81	1.18 (1.02 to 1.36)	0.03	-	-	-	-
Eating dinner with family									
Never	4,021 (27.2)	(reference)	-	(reference)	-	-	-	-	-
Sometimes	1,869 (12.7)	0.99 (0.89 to 1.12)	0.93	1.12 (0.90 to 1.40)	0.30	-	-	-	-
Everyday	8,881 (60.1)	0.95 (0.88 to 1.03)	0.23	1.13 (0.97 to 1.32)	0.11	-	-	-	-

[1]Values are n (%) or RR (95% CI)
[2]Other category included those who identified themselves as students (2,988[20.2%]) or professionals (48[0.3%])

For peer review only

Table S2. Comparison of social determinants of health with BMI categories (underweight [<18.5 kg/m^2], normal weight [18.5–24.9 kg/m^2; base], and overweight and obese [≥25 kg/m^2]) among young women (19–23 y) enrolled in the MaPPS Trial at enrolment (n=10,676)

**Possible determinants**	**n (%)**	**BMI categories**							
		**Crude**				**Multivariable adjusted**			
		Underweight	*P*	Overweight/ obese	*P*	Underweight	*P*	Overweight/ obese	*P*
Level 1: Structural factors – socioeconomic status									
Highest level of education									
None	5,087 (47.6)	(reference)	-	(reference)	-	-	-	-	-
Primary	2,411 (22.6)	0.80 (0.71 to 0.89)	<0.001	1.71 (1.47 to 1.98)	<0.001	-	-	-	-
Secondary or higher	3,178 (29.8)	0.74 (0.66 to 0.82)	<0.001	2.21 (1.94 to 2.52)	<0.001	-	-	-	-
Occupation									
Unskilled manual labour	1,837 (17.2)	(reference)	-	(reference)	-	(reference)	-	(reference)	-
Skilled manual labour	2,372 (22.2)	0.81 (0.71 to 0.93)	0.002	2.77 (2.17 to 3.55)	<0.001	0.95 (0.83 to 1.09)	0.48	1.91 (1.48 to 2.48)	<0.001
Within the home	5,779 (54.1)	0.75 (0.67 to 0.84)	<0.001	3.52 (2.81 to 4.42)	<0.001	0.93 (0.83 to 1.05)	0.25	2.07 (1.61 to 2.67)	<0.001
Other[2]	688 (6.4)	0.76 (0.62 to 0.93)	0.007	3.80 (2.83 to 5.11)	<0.001	1.11 (0.83 to 1.49)	0.47	1.64 (1.19 to 2.26)	0.002
Religion									
Non-Muslim	919 (8.6)	(reference)	-	(reference)	-	(reference)	-	(reference)	-
Muslim	9,757 (91.4)	0.64 (0.56 to 0.74)	<0.001	1.74 (1.35 to 2.25)	<0.001	0.76 (0.61 to 0.93)	0.01	0.98 (0.71 to 1.36)	0.92
Wealth quintile									
Poorest	1,883 (17.6)	(reference)	-	(reference)	-	(reference)	-	(reference)	-
Poor	1,972 (18.5)	0.81 (0.71 to 0.92)	0.002	1.98 (1.48 to 2.64)	<0.001	0.87 (0.74 to 1.02)	0.09	1.81 (1.36 to 2.43)	<0.001
Middle	2,114 (19.8)	0.73 (0.64 to 0.83)	<0.001	3.31 (2.52 to 4.34)	<0.001	0.79 (0.70 to 0.89)	<0.001	2.84 (2.02 to 4.00)	<0.001
Rich	2,215 (20.7)	0.62 (0.54 to 0.71)	<0.001	4.68 (3.60 to 6.09)	<0.001	0.68 (0.59 to 0.78)	<0.001	3.88 (2.85 to 5.28)	<0.001
Richest	2,492 (23.3)	0.55 (0.48 to 0.63)	<0.001	6.36 (4.92 to 8.22)	<0.001	0.59 (0.48 to 0.72)	<0.001	5.24 (3.93 to 6.98)	<0.001
Level 2: Intermediary factors - household and personal characteristics									
Marital status									
Married	4,267 (40.0)	(reference)	-	(reference)	-	(reference)	-	(reference)	-
Unmarried	6,409 (60.0)	1.14 (1.04 to 1.24)	0.004	0.90 (0.81 to 1.01)	0.08	1.25 (1.11 to 1.42)	<0.001	0.77 (0.69 to 0.85)	<0.001
Ever been pregnant									
Yes	2,906 (27.2)	(reference)	-	(reference)	-	-	-	-	-
No	7,770 (72.8)	1.03 (0.93 to 1.13)	0.58	0.93 (0.82 to 1.06)	0.28	-	-	-	-
Household food security status									
Food insecure	2,494 (23.4)	(reference)	-	(reference)	-	-	-	-	-
Food secure	8,182 (76.6)	0.79 (0.71 to 0.87)	<0.001	1.77 (1.52 to 2.07)	<0.001	-	-	-	-
Level 3: Intermediary factors - health and well-being characteristics									
Perception of own health									
Poor or fair	1,275 (11.9)	(reference)	-	(reference)	-	(reference)	-	(reference)	-
Good	6,828 (64.0)	0.78 (0.68 to 0.89)	<0.001	1.18 (0.97 to 1.44)	0.09	0.78 (0.69 to 0.88)	<0.001	1.16 (0.94 to 1.44)	0.18
Excellent	2,573 (24.1)	0.75 (0.64 to 0.87)	<0.001	1.35 (1.09 to 1.67)	0.006	0.78 (0.69 to 0.89)	<0.001	1.16 (0.89 to 1.54)	0.27
Experience of depression-like feelings									
Severe or extremely severe	537 (5.0)	(reference)	-	(reference)	-	-	-	-	-

For peer review only

Possible determinants	n (%)	BMI categories							
		Crude				Multivariable adjusted			
		Underweight	*P*	Overweight/ obese	*P*	Underweight	*P*	Overweight/ obese	*P*
Moderate	1,108 (10.4)	1.02 (0.82 to 1.28)	0.84	1.37 (0.97 to 1.93)	0.08	-	-	-	-
Mild	922 (8.6)	1.01 (0.8 to 1.28)	0.92	1.57 (1.11 to 2.23)	0.01	-	-	-	-
None	8,109 (76.0)	0.87 (0.72 to 1.06)	0.16	1.49 (1.11 to 2.01)	0.009	-	-	-	-
Experience of anxiety-like feelings									
Severe or extremely severe	1,421 (13.3)	(reference)	-	(reference)	-	-	-	-	-
Moderate	1,514 (14.2)	0.94 (0.80 to 1.10)	0.44	0.91 (0.73 to 1.15)	0.44	-	-	-	-
Mild	732 (6.9)	0.71 (0.58 to 0.87)	0.001	0.72 (0.54 to 0.97)	0.03	-	-	-	-
None	7,009 (65.7)	0.81 (0.72 to 0.92)	0.001	1.17 (0.98 to 1.40)	0.08	-	-	-	-
Experience of stress-like feelings									
Severe or extremely severe	405 (3.8)	(reference)	-	(reference)	-	-	-	-	-
Moderate	578 (5.4)	1.15 (0.87 to 1.52)	0.33	1.07 (0.72 to 1.59)	0.74	-	-	-	-
Mild	621 (5.8)	0.96 (0.72 to 1.26)	0.76	1.24 (0.85 to 1.80)	0.27	-	-	-	-
None	9,072 (85.0)	0.88 (0.71 to 1.10)	0.27	1.08 (0.79 to 1.47)	0.62	-	-	-	-
Level 4: Intermediary factors - actions and practices-related characteristics									
Self-efficacy									
Low	2,937 (27.5)	(reference)	-	(reference)	-	-	-	-	-
Moderate	5,709 (53.5)	0.92 (0.83 to 1.02)	0.10	1.34 (1.16 to 1.54)	<0.001	-	-	-	-
High	2,030 (19.0)	0.83 (0.73 to 0.95)	0.005	1.40 (1.18 to 1.66)	<0.001	-	-	-	-
Decision-making autonomy									
All decisions made by family	4,365 (40.9)	(reference)	-	(reference)	-	(reference)	-	(reference)	-
Most decisions made by family	3,630 (34.0)	0.91 (0.83 to 1.01)	0.08	1.08 (0.95 to 1.24)	0.23	0.92 (0.85 to 0.99)	0.03	1.08 (0.93 to 1.27)	0.31
Decisions made jointly with family or autonomously	2,681 (25.1)	0.85 (0.76 to 0.94)	0.003	1.19 (1.04 to 1.37)	0.01	0.85 (0.77 to 0.95)	0.003	1.24 (1.06 to 1.45)	0.01
Skipping breakfast									
Skips breakfast	3,130 (29.3)	(reference)	-	(reference)	-	-	-	-	-
Eats breakfast	7,546 (70.7)	0.93 (0.85 to 1.02)	0.11	1.15 (1.01 to 1.31)	0.03	-	-	-	-
Eating dinner with family									
Never	2,800 (26.2)	(reference)	-	(reference)	-	-	-	-	-
Sometimes	1,620 (15.2)	1.15 (1 to 1.32)	0.05	0.97 (0.81 to 1.17)	0.75	-	-	-	-
Everyday	6,256 (58.6)	1.13 (1.02 to 1.25)	0.02	1.08 (0.94 to 1.23)	0.27	-	-	-	-

[1]Values are n (%) or RR (95% CI)

[2]Other category included those who identified themselves as a student (526[4.9%]) or professional (688[6.4%])

For peer review only

Table S3. Comparison of social determinants of health with stunting (height <145 cm) among late adolescent girls (n=14,771) and adult women (n=10,676) enrolled in the MaPPS Trial at enrolment

Possible determinants	Stunting among late adolescent girls				Stunting among young women			
	Crude		Multivariable adjusted		Crude		Multivariable adjusted	
	OR (95% CI)	*P*	OR (95% CI)	*P*	OR (95% CI)	*P*	OR (95% CI)	*P*
Level 1: Structural factors – socioeconomic status								
Highest level of education								
None	(reference)	-	(reference)	-	(reference)	-	(reference)	-
Primary	0.65 (0.57 to 0.74)	<0.001	0.76 (0.62 to 0.94)	0.01	0.62 (0.51 to 0.74)	<0.001	0.76 (0.63 to 0.93)	0.006
Secondary or higher	0.33 (0.29 to 0.38)	<0.001	0.48 (0.39 to 0.59)	<0.001	0.51 (0.43 to 0.61)	<0.001	0.79 (0.66 to 0.95)	0.01
Occupation								
Unskilled manual labour	(reference)	-	-	-	(reference)	-	-	-
Skilled manual labour	0.56 (0.47 to 0.66)	<0.001	-	-	0.75 (0.61 to 0.91)	0.005	-	-
Within the home	0.69 (0.60 to 0.79)	<0.001	-	-	0.59 (0.49 to 0.71)	<0.001	-	-
Other[1]	0.32 (0.27 to 0.39)	<0.001	-	-	0.45 (0.32 to 0.65)	<0.001	-	-
Religion								
Non-Muslim	(reference)	-	-	-	(reference)	-	-	-
Muslim	0.57 (0.49 to 0.67)	<0.001	-	-	0.74 (0.59 to 0.92)	0.008	-	-
Wealth quintile								
Poorest	(reference)	-	(reference)	-	(reference)	-	(reference)	-
Poor	0.83 (0.72 to 0.97)	0.02	0.91 (0.77 to 1.08)	0.29	0.88 (0.72 to 1.08)	0.22	0.92 (0.73 to 1.17)	0.52
Middle	0.67 (0.57 to 0.78)	<0.001	0.79 (0.65 to 0.96)	0.02	0.65 (0.52 to 0.80)	<0.001	0.70 (0.59 to 0.84)	<0.001
Rich	0.46 (0.39 to 0.55)	<0.001	0.63 (0.49 to 0.81)	<0.001	0.48 (0.38 to 0.60)	<0.001	0.54 (0.40 to 0.74)	<0.001
Richest	0.26 (0.21 to 0.32)	<0.001	0.42 (0.35 to 0.51)	<0.001	0.34 (0.27 to 0.43)	<0.001	0.41 (0.29 to 0.57)	<0.001
Level 2: Intermediary factors - household and personal characteristics								
Marital status								
Married	(reference)	-	-	-	(reference)	-	-	-
Unmarried	0.89 (0.75 to 1.07)	0.21	-	-	0.99 (0.86 to 1.15)	0.93	-	-
Ever been pregnant								
Yes	(reference)	-	-	-	(reference)	-	(reference)	-
No	0.97 (0.74 to 1.27)	0.81	-	-	1.13 (0.96 to 1.32)	0.15	1.27 (1.10 to 1.47)	0.001
Household food security status								
Food insecure	(reference)	-	(reference)	-	(reference)	-	(reference)	-
Food secure	0.58 (0.51 to 0.64)	<0.001	0.77 (0.66 to 0.90)	0.001	0.57 (0.49 to 0.66)	<0.001	0.73 (0.59 to 0.90)	0.003
Level 3: Intermediary factors - health and well-being characteristics								
Perception of own health								
Poor or fair	(reference)	-	-	-	(reference)	-	-	-
Good	0.93 (0.78 to 1.10)	0.38	-	-	0.83 (0.67 to 1.02)	0.07	-	-
Excellent	0.85 (0.70 to 1.04)	0.11	-	-	0.79 (0.62 to 1.00)	0.06	-	-
Experience of depression-like feelings								
Severe or extremely severe	(reference)	-	-	-	(reference)	-	-	-
Moderate	0.93 (0.70 to 1.23)	0.61	-	-	0.98 (0.68 to 1.41)	0.90	-	-
Mild	0.94 (0.71 to 1.26)	0.70	-	-	0.86 (0.58 to 1.26)	0.43	-	-

For peer review only

Possible determinants	Stunting among late adolescent girls				Stunting among young women			
	Crude		Multivariable adjusted		Crude		Multivariable adjusted	
	OR (95% CI)	*P*	OR (95% CI)	*P*	OR (95% CI)	*P*	OR (95% CI)	*P*
None	0.79 (0.62 to 1.00)	0.05	-	-	0.88 (0.64 to 1.19)	0.40	-	-
Experience of anxiety-like feelings								
Severe or extremely severe	(reference)	-	-	-	(reference)	-	-	-
Moderate	0.84 (0.69 to 1.03)	0.09	-	-	0.82 (0.62 to 1.07)	0.14	-	-
Mild	0.96 (0.75 to 1.23)	0.76	-	-	0.99 (0.72 to 1.37)	0.97	-	-
None	0.83 (0.71 to 0.97)	0.02	-	-	0.94 (0.77 to 1.16)	0.56	-	-
Experience of stress-like feelings								
Severe or extremely severe	(reference)	-	-	-	(reference)	-	-	-
Moderate	0.93 (0.64 to 1.34)	0.69	-	-	0.93 (0.58 to 1.49)	0.76	-	-
Mild	1.09 (0.77 to 1.54)	0.63	-	-	0.78 (0.48 to 1.25)	0.30	-	-
None	0.90 (0.68 to 1.20)	0.49	-	-	0.98 (0.68 to 1.40)	0.90	-	-
Level 4: Intermediary factors - actions and practices-related characteristics								
Self-efficacy								
Low	(reference)	-	(reference)		(reference)	-	-	-
Moderate	0.62 (0.56 to 0.70)	<0.001	0.74 (0.64 to 0.84)	<0.001	0.83 (0.71 to 0.98)	0.03	-	-
High	0.59 (0.50 to 0.71)	<0.001	0.74 (0.62 to 0.87)	<0.001	0.78 (0.63 to 0.97)	0.03	-	-
Decision-making autonomy								
All decisions made by family	(reference)	-	(reference)	-	(reference)	-	-	-
Most decisions made by family	0.83 (0.74 to 0.94)	0.003	0.88 (0.76 to 1.01)	0.08	0.99 (0.84 to 1.16)	0.90	-	-
Decisions made jointly with family or autonomously	0.74 (0.62 to 0.89)	0.001	0.76 (0.62 to 0.92)	0.01	0.85 (0.71 to 1.02)	0.09	-	-
Skipping breakfast								
Skips breakfast	(reference)	-	-	-	(reference)	-	-	-
Eats breakfast	0.82 (0.73 to 0.91)	<0.001	-	-	0.79 (0.68 to 0.91)	0.002	-	-
Eating dinner with family								
Never	(reference)		-	-	(reference)	-	-	-
Sometimes	1.09 (0.92 to 1.30)	0.33	-	-	1.19 (0.95 to 1.49)	0.12	-	-
Everyday	0.93 (0.82 to 1.05)	0.23	-	-	1.02 (0.87 to 1.21)	0.79	-	-

[1]Other category included those who identified themselves as a student or profession

For peer review only

Table 1. STROBE-nut: An extension of the STROBE statement for nutritional epidemiology

Lachat C et al. (2016) STrengthening the Reporting of OBservational studies in Epidemiology – Nutritional Epidemiology (STROBE-nut): an extension of the STROBE statement. Plos Medicine 13(6) http://dx.doi.org/10.1371/journal.pmed.1002036 pdf or online version.

Item	Item nr	STROBE recommendations	Extension for Nutritional Epidemiology studies (STROBE-nut)	Reported on page #
**Title and abstract**	1	(a) Indicate the study's design with a commonly used term in the title or the abstract.<br>(b) Provide in the abstract an informative and balanced summary of what was done and what was found.	**nut-1** State the dietary/nutritional assessment method(s) used in the title, abstract, or keywords.	1-3
**Introduction**				
Background rationale	2	Explain the scientific background and rationale for the investigation being reported.		5-7
Objectives	3	State specific objectives, including any pre-specified hypotheses.		6-7
**Methods**				
Study design	4	Present key elements of study design early in the paper.		7
Settings	5	Describe the setting, locations, and relevant dates, including periods of recruitment, exposure, follow-up, and data collection.	**nut-5** Describe any characteristics of the study settings that might affect the dietary intake or	7

Item	Item nr	STROBE recommendations	Extension for Nutritional Epidemiology studies (STROBE-nut)	Reported on page #
			nutritional status of the participants, if applicable.	
Participants	6	a) Cohort study—Give the eligibility criteria, and the sources and methods of selection of participants. Describe methods of follow-up.<br><br>Case-control study—Give the eligibility criteria, and the sources and methods of case ascertainment and control selection. Give the rationale for the choice of cases and controls.<br><br>Cross-sectional study—Give the eligibility criteria, and the sources and methods of selection of participants.<br><br>(b) Cohort study—For matched studies, give matching criteria and number of exposed and unexposed.<br><br>Case-control study—For matched studies, give matching criteria and the number of controls per case.	**nut-6** Report particular dietary, physiological or nutritional characteristics that were considered when selecting the target population.	7-8
Variables	7	Clearly define all outcomes, exposures, predictors, potential confounders, and effect modifiers. Give diagnostic criteria, if applicable.	**nut-7.1** Clearly define foods, food groups, nutrients, or other food components.	8-10

For peer review only - http://bmjopen.bmj.com/site/about/guidelines.xhtml

Item	Item nr	STROBE recommendations	Extension for Nutritional Epidemiology studies (STROBE-nut)	Reported on page #
			**nut-7.2** When using dietary patterns or indices, describe the methods to obtain them and their nutritional properties.	
Data sources - measurements	8	For each variable of interest, give sources of data and details of methods of assessment (measurement).Describe comparability of assessment methods if there is more than one group.	**nut-8.1** Describe the dietary assessment method(s), e.g., portion size estimation, number of days and items recorded, how it was developed and administered, and how quality was assured. Report if and how supplement intake was assessed.<br><br>**nut-8.2** Describe and justify food composition data used. Explain the procedure to match food composition with consumption data. Describe the use of conversion factors, if applicable.<br><br>**nut-8.3** Describe the nutrient requirements, recommendations, or dietary guidelines and the evaluation approach used to compare intake with the dietary reference values, if applicable.<br><br>**nut-8.4** When using nutritional biomarkers, additionally use the STROBE Extension for Molecular Epidemiology (STROBE-ME). Report the type of biomarkers used and their usefulness as dietary exposure markers.	8-10

For peer review only

Item	Item nr	STROBE recommendations	Extension for Nutritional Epidemiology studies (STROBE-nut)	Reported on page #
			**nut-8.5** Describe the assessment of nondietary data (e.g., nutritional status and influencing factors) and timing of the assessment of these variables in relation to dietary assessment.<br><br>**nut-8.6** Report on the validity of the dietary or nutritional assessment methods and any internal or external validation used in the study, if applicable.	
Bias	9	Describe any efforts to address potential sources of bias.	**nut-9** Report how bias in dietary or nutritional assessment was addressed, e.g., misreporting, changes in habits as a result of being measured, or data imputation from other sources	8-10
Study Size	10	Explain how the study size was arrived at.		7
Quantitative variables	11	Explain how quantitative variables were handled in the analyses. If applicable, describe which groupings were chosen and why.	**nut-11** Explain categorization of dietary/nutritional data (e.g., use of N-tiles and handling of nonconsumers) and the choice of reference category, if applicable.	8-10
Statistical Methods	12	(a) Describe all statistical methods, including those used to control for confounding<br><br>(b) Describe any methods used to examine subgroups and interactions.	**nut-12.1** Describe any statistical method used to combine dietary or nutritional data, if applicable.	10-12

For peer review only

Item	Item nr	STROBE recommendations	Extension for Nutritional Epidemiology studies (STROBE-nut)	Reported on page #
		(c) Explain how missing data were addressed.<br><br>(d) Cohort study—If applicable, explain how loss to follow-up was addressed.<br><br>Case-control study—If applicable, explain how matching of cases and controls was addressed.<br><br>Cross-sectional study—If applicable, describe analytical methods taking account of sampling strategy.<br><br>(e) Describe any sensitivity analyses.	**nut-12.2** Describe and justify the method for energy adjustments, intake modeling, and use of weighting factors, if applicable.<br><br>**nut-12.3** Report any adjustments for measurement error, i.e,. from a validity or calibration study.	
**Results**				
Participants	13	(a) Report the numbers of individuals at each stage of the study—e.g., numbers potentially eligible, examined for eligibility, confirmed eligible, included in the study, completing follow-up, and analyzed.<br><br>(b) Give reasons for non-participation at each stage.<br><br>(c) Consider use of a flow diagram.	**nut-13** Report the number of individuals excluded based on missing, incomplete or implausible dietary/nutritional data.	12
Descriptive data	14	(a) Give characteristics of study participants (e.g., demographic, clinical, social) and	**nut-14** Give the distribution of participant characteristics across the exposure variables if applicable. Specify if food consumption of	12-13, Table 1

For peer review only

Item	Item nr	STROBE recommendations	Extension for Nutritional Epidemiology studies (STROBE-nut)	Reported on page #
		information on exposures and potential confounders<br>(b) Indicate the number of participants with missing data for each variable of interest<br>(c) Cohort study—Summarize follow-up time (e.g., average and total amount)	total population or consumers only were used to obtain results.	
Outcome data	15	Cohort study—Report numbers of outcome events or summary measures over time.<br>Case-control study—Report numbers in each exposure category, or summary measures of exposure.<br>Cross-sectional study—Report numbers of outcome events or summary measures.		14-16, Table 1
Main results	16	(a) Give unadjusted estimates and, if applicable, confounder-adjusted estimates and their precision (e.g., 95% confidence interval).<br>Make clear which confounders were adjusted for and why they were included.<br>(b) Report category boundaries when continuous variables were categorized.	**nut-16** Specify if nutrient intakes are reported with or without inclusion of dietary supplement intake, if applicable.	14-16, Tables 2-4

For peer review only - http://bmjopen.bmj.com/site/about/guidelines.xhtml

Item	Item nr	STROBE recommendations	Extension for Nutritional Epidemiology studies (STROBE-nut)	Reported on page #
		(c) If relevant, consider translating estimates of relative risk into absolute risk for a meaningful time period.		
Other analyses	17	Report other analyses done—e.g., analyses of subgroups and interactions and sensitivity analyses.	**nut-17** Report any sensitivity analysis (e.g., exclusion of misreporters or outliers) and data imputation, if applicable.	N/A
**Discussion**				
Key results	18	Summarize key results with reference to study objectives.		20
Limitation	19	Discuss limitations of the study, taking into account sources of potential bias or imprecision. Discuss both direction and magnitude of any potential bias.	**nut-19** Describe the main limitations of the data sources and assessment methods used and implications for the interpretation of the findings.	22-23
Interpretation	20	Give a cautious overall interpretation of results considering objectives, limitations, multiplicity of analyses, results from similar studies, and other relevant evidence.	**nut-20** Report the nutritional relevance of the findings, given the complexity of diet or nutrition as an exposure.	20-22
Generalizability	21	Discuss the generalizability (external validity) of the study results.		20-21
**Other information**				
Funding	22	Give the source of funding and the role of the funders for the present study and, if		24

For peer review only

Item	Item nr	STROBE recommendations	Extension for Nutritional Epidemiology studies (STROBE-nut)	Reported on page #
		applicable, for the original study on which the present article is based.		
Ethics			**nut-22.1** Describe the procedure for consent and study approval from ethics committee(s).	24
Supplementary material			**nut-22.2** Provide data collection tools and data as online material or explain how they can be accessed.	Yes

For peer review only

BMJ Open

Drivers of malnutrition among late adolescent and young women in rural Pakistan: A cross-sectional assessment of the MaPPS Trial

Journal:	*BMJ Open*
Manuscript ID	bmjopen-2022-063734.R1
Article Type:	Original research
Date Submitted by the Author:	16-Jan-2023
Complete List of Authors:	Baxter, Jo-Anna; The Hospital for Sick Children,<br>Wasan, Yaqub; Aga Khan University, Centre of Excellence in Women and Child Health<br>Hussain, Amjad; Aga Khan University, Pediatrics and Child Health<br>Soofi, Sajid; The Aga Khan University, Pediatrics & Child Health<br>Ahmed, Imran; Aga Khan University<br>Bhutta, Zulfiqar; The Aga Khan University, Division of Women and Child Health
**Primary Subject Heading**:	Global health
Secondary Subject Heading:	Nutrition and metabolism, Public health, Epidemiology
Keywords:	NUTRITION & DIETETICS, PUBLIC HEALTH, Community child health < PAEDIATRICS

SCHOLARONE™
Manuscripts

I, the Submitting Author has the right to grant and does grant on behalf of all authors of the Work (as defined in the below author licence), an exclusive licence and/or a non-exclusive licence for contributions from authors who are: i) UK Crown employees; ii) where BMJ has agreed a CC-BY licence shall apply, and/or iii) in accordance with the terms applicable for US Federal Government officers or employees acting as part of their official duties; on a worldwide, perpetual, irrevocable, royalty-free basis to BMJ Publishing Group Ltd ("BMJ") its licensees and where the relevant Journal is co-owned by BMJ to the co-owners of the Journal, to publish the Work in this journal and any other BMJ products and to exploit all rights, as set out in our licence.

The Submitting Author accepts and understands that any supply made under these terms is made by BMJ to the Submitting Author unless you are acting as an employee on behalf of your employer or a postgraduate student of an affiliated institution which is paying any applicable article publishing charge ("APC") for Open Access articles. Where the Submitting Author wishes to make the Work available on an Open Access basis (and intends to pay the relevant APC), the terms of reuse of such Open Access shall be governed by a Creative Commons licence – details of these licences and which Creative Commons licence will apply to this Work are set out in our licence referred to above.

Other than as permitted in any relevant BMJ Author's Self Archiving Policies, I confirm this Work has not been accepted for publication elsewhere, is not being considered for publication elsewhere and does not duplicate material already published. I confirm all authors consent to publication of this Work and authorise the granting of this licence.

For peer review only

TITLE PAGE

Title:

Drivers of malnutrition among late adolescent and young women in rural Pakistan: A cross-sectional assessment of the MaPPS Trial

Authors:

Jo-Anna B. Baxter[1,2], Yaqub Wasan[3], Amjad Hussain[3], Sajid B. Soofi[3], Imran Ahmed[3], Zulfiqar A. Bhutta[1-4]

Affiliations:

[1]Centre for Global Child Health, Hospital for Sick Children, Toronto, Canada

[2]Department of Nutritional Sciences, University of Toronto, Toronto, Canada

[3]Centre of Excellence in Women and Child Health, Aga Khan University, Karachi, Pakistan

[4] Dalla Lana School of Public Health, University of Toronto, Toronto, Canada

Corresponding author:

Dr. Zulfiqar A. Bhutta

Telephone number: +92 21 34930051

Email address: zulfiqar.bhutta@aku.edu

Postal address: Center of Excellence in Women and Child Health, Aga Khan University, Karachi 74800, Pakistan

Word count:

For peer review only

4041

Tables:

4

Figures:

0

Online supplemental material submitted:

Yes

ARTICLE SUMMARY

Abstract:

Objective: This study aimed to characterize the burden of malnutrition and assess how underlying determinants at the structural and intermediary levels contributed to malnutrition among late adolescent and young women in rural Pakistan.

Design: Cross-sectional enrolment data assessment.

Setting and participants: This study was conducted using data from adolescent and young women (n=25,447) enrolled in the Matiari emPowerment and Preconception Supplementation (MaPPS) Trial, collected from June 2017 to July 2018 in Matiari District, Pakistan. The WHO-based cut-offs were applied to anthropometric measures to estimate BMI categories

For peer review only

(underweight, overweight, obese) and stunting. Hierarchical models were generated to evaluate the association between the determinants with BMI categories and stunting among late adolescent girls and young women, respectively.

Primary and secondary outcome measures: The main outcomes of interest were BMI categories and stunting. Explanatory variables included measures of socioeconomic status, education, occupation, health, well-being, food security, empowerment, and food practices.

Results: Regardless of age group, the prevalence of underweight was high (36.9%; 95% CI: 36.3 to 37.5%). More late adolescent girls were underweight, while more young women were overweight/obese (P<0.001). Stunting affected 9.2% (95% CI: 8.9 to 9.6%) of participants, of which 35.7% were additionally underweight and 7.3% overweight/obese. Compared to those in the normal weight category, those underweight were more likely to be impoverished and less empowered. Those overweight/obese were more likely to be from a higher wealth quintile and food secure. Increased education level and food security were associated with reductions stunting risk.

Conclusions: This study informs the data gap and need for comprehensive research on adolescent nutritional status. Findings suggest factors related to poverty played an important, underlying role in undernutrition among participants. Commitment to improving the nutritional status of all adolescent and young women in Pakistan will be critical given the observed burden of malnutrition.

Trial registration: Clinicaltrials.gov NCT03287882.

Strengths and limitations of this study:

- Although the catchment area of this study was restricted to the areas covered by the LHW Programme, the study is very large and representative of rural setting in Pakistan.

For peer review only - http://bmjopen.bmj.com/site/about/guidelines.xhtml

- Given the cross-sectional design of the study, we cannot suggest causation.
- All anthropometric measurements (weight, height, middle upper arm circumference) were collected in duplicate following standardized procedures by trained study personnel, although we did not collect additional measures of body composition (e.g., skin-fold thickness, abdominal circumference).
- We used validated questionnaires to assess social determinants of health at both the structural and intermediary levels.

KEYWORDS

body mass index, stunting, adolescent girl, young woman, undernutrition, underweight, overweight, obesity

For peer review only

INTRODUCTION

Adolescence is a life stage characterized by substantial physical, cognitive, social, and emotional development, and nutritional status importantly underlies the transition from childhood to adulthood.[1] Defined by the World Health Organization (WHO) as the period from 10–19 y, some suggest adolescence extends to 24 y given continued cortical development.[1 2] Being malnourished during the adolescent period is associated with poor health outcomes throughout the life-course and has potential intergenerational effects.[3 4] In South Asia, undernutrition affects >38% of adolescent girls; there has been limited decline in the past decade and the increasing occurrence of overweight/obesity is of additional concern.[5]

Anthropometric measures can serve as a useful indicator for assessing malnutrition. Measures of undernutrition, including underweight (thinness) and stunting (short stature), are determined from body mass index (BMI) and attained height, respectively. While underweight reflects current nutritional status caused by insufficient or imbalanced intake and/or underlying disease, stunting reflects chronic or recurrent undernourishment.[6] Being underweight and/or stunted has been found to be associated with adverse health consequences to the individual, and, in the event of a pregnancy, increased risk for adverse pregnancy outcomes.[7] Conversely, overweight and obesity have been linked to the early onset of non-communicable diseases and premature mortality.[8 9]

Social determinants of health (SDoH) are described to influence nutritional inequalities, making understanding their role in malnutrition of importance.[10] SDoH are typically divided into two classes, structural and intermediary.[11] Structural SDoH underlie food availability and access, including stratifiers such as ethnicity, education, occupation, and income. Intermediary SDoH are

For peer review only - http://bmjopen.bmj.com/site/about/guidelines.xhtml

more proximal to nutrition-related outcomes, affecting material circumstances and biological, psychological, and behavioural factors.[12]

With the focus of the Sustainable Development Goals on equity, and the explicit aim in Goal 5 to empower all women and girls,[13] measuring, monitoring, and researching women's empowerment is of importance. Adolescent girls are an understudied population with respect to empowerment, and understanding the impact of interventions that aim to improve empowerment is a noted research gap.[1] Proxies for empowerment span SDoH at both the structural (e.g., education) and intermediary levels (e.g., self-efficacy and contribution to decision-making). They are suggested to influence nutritional status, although this association has predominantly been made between maternal empowerment and child nutritional status.[14-16] The WHO acknowledges that empowering adolescent girls, families, and communities is an important component to improving overall health and nutritional status.[1]

At this time, there are more young people than ever before in Pakistan, with around 30% of the population between 15-24 y.[17] Nationally representative surveys have traditionally focused on married women 15–49 y given the importance of reproductive outcomes, and estimate that the prevalence of underweight and stunting are high at 8.5 and 5.0%, respectively, and that there is an emerging burden of overweight and obesity.[17] However, there has been limited assessment among late adolescent girls and unmarried young women, thus there is a gap in information within this segment of the population. We aimed to assess the prevalence of malnutrition using anthropometric measures from all adolescent and young women (15–23 y) living in rural Pakistan, and determine whether structural and intermediary SDoH were associated with underweight, overweight/obese, and stunting. Understanding the diverse SDoH that predict

For peer review only

malnutrition will be informative to policy generation and the prioritization of programming to address current nutritional challenges.

METHODS

This study used cross-sectional data from late adolescent girls and young women (15−23 y) collected at enrolment within the Matiari emPowerment and Preconception Supplementation (MaPPS) Trial.

Setting and study design

The MaPPS Trial is a two-arm, cluster-randomized, controlled trial that aims to assess the impact of life skills building education and multiple micronutrient supplementation on several nutrition, health, and empowerment-related outcomes in rural Pakistan. The study methodology and data collection tools employed within this population-based effectiveness trial have been previously described.[18] The trial was registered at Clinicaltrials.gov as NCT03287882. The MaPPS Trial aimed to recruit approximately 25,400 non-pregnant adolescent and young women to observe enough pregnancies to detect a 25% relative reduction in low-birth-weight births (primary trial outcome), assuming prevalence of low birth weight of 30%, icc=0.011, k=0.16, accounting for 10% attrition, and probability of type I and type II errors of 0.05 and 0.20, respectively.

Participant enrolment

Participants were recruited to the MaPPS Trial from the end of June 2017 to July 2018, using a cluster-wise roll-out. The cluster unit was the catchment population of pre-identified health

For peer review only

facilities and their respective outreach community health workers (Lady Health Workers; LHWs) (n=26). Late adolescent girls and young women 15–23 y were identified from a household listing exercise conducted from December 2016 to May 2017, wherein all households covered by LHWs in a cluster unit were surveyed by study personnel. To screen for participation in the MaPPS Trial, late adolescent girls and young women 15–23 y were approached at their homes by study personnel to further assess their eligibility and ≥1 participant could be enrolled per household. Eligibility criteria included reporting being non-pregnant; physically able to comply with supplementation; not participating in any other nutrition studies; and intending to remain in the study area for the duration of the trial. The purpose and voluntary nature of the trial was verbally explained following confirmation of eligibility by study personnel and a written consent form was provided. Written documentation of informed consent was obtained as either a signature or a thumb impression, in the case that a participant was illiterate.

Patient and public involvement

Prior to the start of the household listing exercise, a wide range of activities were undertaken to obtain local approval of the MaPPS Trial. This included sharing information about what was being done within the MaPPS Trial and why with the district education department and health leaders (including the LHW Programme), and leaders from participating villages. The broader community and prospective participants were not invited to comment on the study design, and they were not consulted on the study outcomes or result interpretation for this research.

Data collection

Upon enrolment, a questionnaire was administered to collect information on participant demographics; socioeconomic status; reproductive health; general health, well-being, and

For peer review only

nutrition; and empowerment-related variables. Anthropometric (height, weight, middle-upper arm circumference) measurements were collected in duplicate by two trained data collectors using standardized methods.[19] Pre-set allowable differences were employed (height: <1.0 cm; weight: <0.5 kg), and a third measurement was collected if two measurements exceed this. In all analyses, the average of two acceptable measures was used. The technical error of measurement (TEM), relative TEM (%TEM), and coefficient of reliability (R) were calculated for height and weight.[20] Field monitoring teams additionally conducted regular data quality spot checks.[18] Data collectors were blinded to study arm allocation.

Outcomes of interest

The outcomes of interest in the present study were cut-offs for BMI (underweight: <18.5 kg/m^2, normal: 18.5–24.9 kg/m^2, overweight: 25–29.9 kg/m^2, obese: ≥30 kg/m^2) and stunting (height <145 cm).[21] BMI was considered categorically because of the increased health risks when it is too low or high. There are known issues with the adolescent-specific cut-offs for BMI and stunting (10–19 y),[22 23] thus we applied the WHO cut-offs for women of reproductive age (WRA; 15–49 y). This is common in international reporting given the age range overlap. Furthermore, the adult cut-offs are tied to reproductive outcomes and adolescent pregnancies were common in the study population.[17] For completeness, since the WHO Growth Reference is suggested to classify the growth of adolescents up to 19 y (228 completed months), we also applied the WHO igrowup package for Stata (StataCorp, College Station, TX) to adolescents' anthropometric data to determine height-for-age and BMI-for-age z-scores (HAZ and BAZ, respectively).[24]

Explanatory variables

For peer review only

Multiple explanatory variables were hypothesized to underlie the nutritional status outcomes of interest. The selection of underlying SDoH risk factors followed our previously published conceptual framework,[25] which was guided by the UNICEF framework on the causes of malnutrition[26] and the WHO framework on SDoH related to health and well-being.[27] These were split into two categories: structural (education, occupation, socioeconomic status, and religion) and intermediary (health, well-being, food insecurity, empowerment, and food practices).

Indicators for demographic information, socioeconomic status, and reproductive health were adapted from the Pakistan Demographic Health Survey (PDHS).[17] A variable for wealth quintile was derived using a principal component analysis from factors related to home characteristics and household asset ownership.[17] Other health-related proxies included self-reported health status and mental health (experience of depression, anxiety, and stress-related emotions over the preceding week; Depression, Anxiety, and Stress Score – 21 Items).[28] Other nutrition-related proxies included household food insecurity (Household Food Insecurity Access Scale)[29] and skipping breakfast and sharing dinner with family (Health Behaviour in School-aged Children Survey).[30] Empowerment proxies included generalized self-efficacy (Generalized Self-Efficacy Scale)[31] and decision-making autonomy (PDHS).[17] Self-efficacy scores were categorized into tertiles, corresponding to low, moderate, and high. A variable for decision-making autonomy was derived from reported participation in 5 decisions, adapted from the PDHS (decisions: own food consumption, own health care, household food purchases, household food distribution, household purchases). The scoring of decision-making participation was consistent with the SWPER index for WRA[32] and participants were categorized based on their involvement (all decisions made by family, most decisions made by family, decisions made jointly with family or autonomously).

For peer review only

Statistical analyses

Analyses were performed using Stata, version 15.0 (Stata Corporation). Since the enrolment data were collected prior to the study intervention, data were analyzed cross-sectionally, independent of study arm assignment. To describe the study population, we used descriptive statistics, including means and standard deviations (SD) and proportions for continuous and categorical variables, respectively. To be consistent with the standard age-based reporting of adolescent anthropometric measures, demographic and anthropometric outcomes were further disaggregated by age group (late adolescent girls: 15–18.9 y; young women: 19–23 y), and a chi-squared test or t-test was used to determine whether there was a difference between proportions and continuous measures, respectively.

We aimed to generate a hierarchical, multivariable model to examine the associations between the SDoH explanatory variables and BMI categories (base: normal BMI [18.5–24.9 kg/m^2]) and stunting (base: height >145 cm), respectively, using our conceptual framework as a guide. The categories for overweight and obese were combined for the BMI variable, given the low prevalence of obesity observed. Because we found that there was a difference in the prevalence of both BMI categories and stunting between late adolescent girls and young women, and to be consistent with standard age-based reporting of anthropometric measures, a separate model was generated for each age group.

The multi-level model building approach described by Victora et al[33] was followed to generate an adapted hierarchical model with 4-levels. Guided by our conceptual model,[25] we hypothesized that there were four levels of SDoH explanatory variables within the hierarchy and mapped corresponding variables: (1) socioeconomic status (distal-most level): education, occupation,

For peer review only - http://bmjopen.bmj.com/site/about/guidelines.xhtml

religion, and wealth quintile; (2) household and personal factors: marital status, parity, and household food security; (3) health and well-being: perception of own health, experience of depression-, anxiety-, and stress-like feelings; and (4) actions and practices (proximal-most level): self-efficacy, participation in decision making, skipping breakfast, and eating dinner with family. For all analyses, the reference for each SDoH explanatory variable was set as the category hypothesized to present the greatest nutritional vulnerability.[25] To investigate the association between the SDoH explanatory variables and each primary outcome measure, crude analyses were first conducted using logistic regression. Variables were considered potentially relevant for inclusion in the multivariable hierarchical model if the unadjusted *P* was <0.20.[34] Starting with the distal-most level, relevant variables were entered into the model. At each level, a backward-stepwise model building approach was used. Variables were serially removed to observe any change in the β-coefficients of those variables maintained in the model and find a model that best explained the variability in the data. When the *P* of an explanatory variable was <0.05, it was retained in final model. If the *P* was ≥0.05 and/or the standard error was high, exclusion of the variable was explored. If the β-coefficients of other variables in the model changed by >10%, the excluded variable was returned, otherwise the variable was dropped. Multicollinearity between variables was assessed using the variance inflation factor (VIF), although we did not find that multicollinearity affected the selected variables using the criteria VIF≥5. The effect estimates in the final model for retained variables correspond to the point of addition to the model. Effect estimates are presented as relative risk ratios (RR) for the variable for BMI categories (three possible outcomes), and odds ratios (OR) for stunting (two possible outcomes). Given the cluster-randomized design of the MaPPS Trial, all models employed cluster robust-standard errors to account for possible correlation between clusters.

For peer review only

RESULTS

Enrolment and exclusion

Of the 32,141 adolescent and young women 15–23 y identified from the household listing exercise, 28,195 (87.7 %) were located, and 25,447 (79.2%) consented to participate in the trial and completed the enrolment questionnaire. Among those enrolled, 14,771 (58.0%) were 15–18.9 y and 10,676 (42.0 %) were 19–23 y, representing 17,044 households.

Participant demographic and reproductive characteristics

At the time of enrolment, participants' mean age (± SD) was 18.8 ± 2.3 y (**Table 1**). No formal education was reported by nearly half of participants, and those with schooling had completed 7.7 ± 3.3 y on average. Currently being a student was reported by 13.8% of participants, of which only 61.1% reported regular attendance. Employment outside the home was reported by 38.5% of participants. Common occupations were unskilled and skilled manual labour (17.5% and 20.2% of participants, respectively), although the majority worked within the home (47.5%). Nearly all participants had experienced menarche (25,305 [99.4%]), with the mean age of onset at 13.0 ± 0.9 y (range: 10–21 y). 22.3% of participants were married, with the mean age at marriage of 16.9 ± 1.8 y (range: 13–23 y), and 13.7% had been pregnant. On average, those married were 4.8 ± 4.2 y younger than their husbands.

For peer review only

Table 1. Demographic and anthropometric characteristics among MaPPS Trial participants at enrolment, disaggregated by age group[1]

**Characteristic**	**All participants** (n=25,447)	**15–18.9 y** (n=14,771)	**19–23 y** (n=10,676)	***P***
Age (y)	18.8 ± 2.3	17.1 ± 1.2	21.1 ± 1.2	<0.001
Highest level of education completed				
None	11,384 (44.7)	4,572 (42.0)	2,758 (44.5)	<0.001
Primary	6,063 (23.8)	2,721 (25.0)	1,412 (22.8)	
Secondary or higher	8,000 (31.5)	4,822 (32.6)	3,178 (29.8)	
Employed outside the home	9,620 (38.1)	5,395 (36.6)	4,225 (40.1)	<0.001
Muslim	23,045 (90.6)	13,288 (90.0)	9,757 (91.4)	<0.001
Wealth quintile				
Poorest	4,549 (17.9)	2,666 (18.0)	1,883 (17.6)	<0.001
Poor	4,853 (19.1)	2,881 (19.5)	1,972 (18.5)	
Middle	5,139 (20.2)	3,025 (20.5)	2,114 (19.8)	
Rich	5,360 (21.1)	3,145 (21.3)	2,215 (20.7)	
Richest	5,546 (21.8)	3,054 (20.7)	2,492 (23.3)	
Currently married[2]	5,685 (22.3)	1,418 (9.6)	4,267 (40.0)	<0.001
Age when married husband (y)[3]	16.9 ± 1.8	15.9 ± 1.1	17.2 ± 1.8	<0.001
Has been pregnant[4]	3,478 (13.7)	572 (3.9)	2,906 (27.2)	<0.001
Age at first pregnancy[5]	17.5 ± 1.6	16.4 ± 1.1	17.7 ± 1.6	<0.001
Weight (kg)	47.0 ± 9.4	45.6 ± 8.5	48.9 ± 10.2	<0.001
Height (cm)	152.5 ± 5.7	152.0 ± 5.6	152.7 ± 5.8	<0.001
BMI (kg/m^2)	20.2 ± 3.7	19.7 ± 3.3	20.9 ± 4.0	<0.001
BMI categorization				
Underweight (<18.5 kg/m^2)	9,396 (36.9)	6,105 (41.3)	3,291 (30.8)	<0.001
Normal (18.5-24.9 kg/m^2)	13,463 (52.9)	7,597 (51.4)	5,866 (54.9)	
Overweight (25-29.9 kg/m^2)	2,011 (7.9)	873 (5.9)	1,138 (10.7)	
Obese (≥30 kg/m^2)	577 (2.3)	196 (1.3)	381 (3.6)	
Middle upper arm circumference (cm)	23.7 ± 3.2	23.3 ± 2.9	24.4 ± 3.4	<0.001

[1]Values are n (%) or mean ± SD

[2]158 participants reported no longer being married

[3]Asked of ever-married women (n_{all} = 5834 [3 women did not know]; $n_{15\text{-}18.9y}$=1,457; $n_{19\text{-}23y}$=4,377)

[4]Due to cultural sensitivities, question only asked of ever-married women (n_{all}=3,451 [28 women did not know]; $n_{15\text{-}18.9y}$=1,457; $n_{19\text{-}23y}$=4,377)

[5]n_{all}=3,451 (28 women did not know); $n_{15\text{-}18.9y}$=567; $n_{19\text{-}23y}$=2,884

For peer review only

Anthropometric characteristics

Regardless of age group, the majority of participants were of 'normal' body weight (52.9%; **Table 1**), although prevalence of underweight was high (36.9%). More late adolescent girls appeared to be underweight, while more young women were overweight/obese (*P*<0.001). Height was <145 cm among 9.2% of participants (late adolescent girls: 10.2%, young women: 7.9%; *P* <0.001), and 35.7% of those stunted were additionally underweight (late adolescent girls: 39.6%,

young women: 28.7%; P <0.001) and 7.3% overweight/obese (late adolescent girls: 5.4%, young women: 10.7%; P=0.006). The TEM for weight and height were 0.22 and 0.18, respectively; %TEM were 0.47% and 0.12%; and the R for each measure was >99%, representing high reliability between the repeat measurements. Among adolescent participants <19 y, HAZ and BAZ were found to be -1.58 ± 0.83 and -0.67 ± 1.13, respectively.

Hierarchical model generation

BMI categories and SDoH risk factors among late adolescent girls

For the hierarchical model looking at BMI categories among late adolescent girls, of the SDoH risk factors identified from the crude analyses, those maintained in the final model included occupation, wealth quintile, marital status, food insecurity, reported perception of health, and self-efficacy (P for model: <0.0001; **Table 2**). While a crude association was observed between BMI categories with education level, religion, decision-making, and skipping breakfast, respectively, this was not maintained in the multivariable model (see online supplemental material [Table S1]). Compared to those with a normal BMI, those underweight were more likely to be from a poorer wealth quintile, unmarried, food insecure, report poorer overall health, and have lower self-efficacy. Among those overweight/obese, compared to those with a normal BMI, someone was less likely to be employed as an unskilled manual labourer and unmarried, and more likely to be from a higher wealth quintile, food secure, and report good health.

BMI categories and SDoH risk factors among young women

Among young women, SDoH risk factors maintained in the final hierarchical model for BMI included occupation, religion, wealth quintile, marital status, reported perception of health, and decision-making autonomy (P for model: <0.0001; **Table 3**). While a crude association was

For peer review only

observed between BMI categories with food security and self-efficacy, respectively, this was not maintained in the multivariable model (see online supplemental material [Table S2]). Compared to those with a normal BMI, those who were underweight were more likely to be from a poorer wealth quintile, non-Muslim, unmarried, and report poorer overall health and participate in less decision-making. Among those overweight/obese, compared to those with a normal BMI, someone was less likely to be employed as an unskilled manual labourer, but more likely to be from a higher wealth quintile and married.

Stunting among late adolescent girls and young women

The SDoH risk factors maintained in hierarchical models for stunting differed between late adolescent girls and young women (**Table 4**). Among late adolescent girls, the final model included variables from three levels of the hierarchy (level 1: education, wealth quintile; level 2: food security; level 4: self-efficacy, decision-making; P for model: <0.0001), while for young women, variables from only the two distal-most levels were maintained (level 1: education, wealth quintile; level 2: parity, food security; P for model: <0.0001). The crude association observed between stunting and occupation, religion, and skipping breakfast, respectively, was not maintained in either multivariable model (see online supplemental material [Table S3]). Compared to those non-stunted, the odds were lower that stunted adolescents had any education or came from a wealthier or food secure household; they experienced lower self-efficacy and made fewer autonomous decisions. Similarly, stunted young women were found to have a lower odd of education and being from a food secure household, as well as a higher odd of participating in unskilled manual labour; once the other variables were considered in the model, they were more likely not to have been pregnant compared to those non-stunted.

For peer review only - http://bmjopen.bmj.com/site/about/guidelines.xhtml

Table 2. Social determinants of health maintained in the hierarchical model of BMI categories (underweight [<18.5 kg/m^2], normal weight [18.5–24.9 kg/m^2; base], and overweight and obese [≥25 kg/m^2]) among late adolescent girls (15–18.9 y) enrolled in the MaPPS Trial at enrolment (n=14,771)[1]

**Possible determinants**	**n (%)**	**BMI categories**							
		**Crude**				**Multivariable adjusted**			
		Underweight	*P*	Overweight/ obese	*P*	Underweight	*P*	Overweight/ obese	*P*
Level 1: Structural factors – socioeconomic status									
Occupation									
Unskilled manual labour	2,622 (17.8)	(reference)	-	(reference)	-	(reference)	-	(reference)	-
Skilled manual labour	2,762 (18.7)	0.85 (0.76 to 0.95)	0.004	2.44 (1.83 to 3.25)	<0.001	0.92 (0.79 to 1.07)	0.26	1.83 (1.42 to 2.36)	<0.001
Within the home	6,351 (43.0)	0.83 (0.75 to 0.91)	<0.001	3.28 (2.54 to 4.25)	<0.001	0.94 (0.83 to 1.07)	0.34	2.03 (1.61 to 2.58)	<0.001
Other[2]	3,036 (20.6)	0.90 (0.81 to 1.00)	0.06	3.26 (2.48 to 4.30)	<0.001	1.14 (0.94 to 1.37)	0.18	1.44 (1.09 to 1.92)	0.01
Wealth quintile									
Poorest	2,666 (18.0)	(reference)	-	(reference)	-	(reference)	-	(reference)	-
Poor	2,881 (19.5)	0.84 (0.76 to 0.94)	0.002	1.47 (1.07 to 2.01)	0.001	0.84 (0.74 to 0.95)	0.01	1.34 (1.07 to 1.67)	0.01
Middle	3,025 (20.5)	0.79 (0.71 to 0.88)	<0.001	2.36 (1.77 to 3.15)	<0.001	0.78 (0.68 to 0.89)	<0.001	2.07 (1.63 to 2.63)	<0.001
Rich	3,145 (21.3)	0.71 (0.64 to 0.79)	<0.001	3.42 (2.59 to 4.51)	<0.001	0.69 (0.58 to 0.82)	<0.001	2.94 (2.32 to 3.74)	<0.001
Richest	3,054 (20.7)	0.69 (0.62 to 0.77)	<0.001	5.18 (3.96 to 6.79)	<0.001	0.65 (0.56 to 0.74)	<0.001	4.65 (3.49 to 6.20)	<0.001
Level 2: Intermediary factors - household and personal characteristics									
Marital status									
Married	1,418 (9.6)	(reference)		(reference)		(reference)	-	(reference)	-
Unmarried	13,353 (90.4)	1.38 (1.23 to 1.56)	<0.001	0.80 (0.66 to 0.97)	<0.03	1.42 (1.28 to 1.59)	<0.001	0.71 (0.59 to 0.85)	<0.001
Household food security status									
Food insecure	3,861 (26.1)	(reference)		(reference)		(reference)	-	(reference)	-
Food secure	10,910 (73.9)	0.83 (0.77 to 0.90)	<0.001	1.92 (1.61 to 2.28)	<0.001	0.91 (0.83 to 0.99)	0.04	1.24 (1.03 to 1.51)	0.03
Level 3: Intermediary factors - health and well-being characteristics									
Perception of own health									
Poor or fair	1,533 (10.4)	(reference)		(reference)		(reference)	-	(reference)	-
Good	9,731 (65.9)	0.82 (0.74 to 0.92)	0.001	1.23 (0.97 to 1.57)	0.09	0.83 (0.70 to 0.97)	0.03	1.23 (0.89 to 1.68)	0.21
Excellent	3,507 (23.7)	0.71 (0.63 to 0.80)	<0.001	1.40 (1.08 to 1.81)	0.01	0.74 (0.62 to 0.88)	0.001	1.22 (0.93 to 1.60)	0.16
Level 4: Intermediary factors - actions and practices-related characteristics									
Self-efficacy									
Low	5,695 (38.6)	(reference)	-	(reference)	-	(reference)	-	(reference)	-
Moderate	7,161 (48.5)	0.85 (0.79 to 0.92)	<0.001	1.27 (1.10 to 1.46)	0.001	0.91 (0.85 to 0.97)	<0.001	1.09 (0.93 to 1.27)	0.29
High	1,915 (13.0)	0.75 (0.67 to 0.83)	<0.001	1.12 (0.91 to 1.37)	0.29	0.82 (0.72 to 0.92)	<0.001	0.91 (0.73 to 1.14)	0.41

[1]Values are n (%) or RR (95% CI)

[2]Other category included those who identified themselves as students (2,988[20.2%]) or professionals (48[0.3%])

For peer review only - http://bmjopen.bmj.com/site/about/guidelines.xhtml

Table 3. Social determinants of health maintained in the hierarchical model of BMI categories (underweight [<18.5 kg/m^2], normal weight [18.5–24.9 kg/m^2; base], and overweight and obese [≥25 kg/m^2]) among young women (19–23 y) enrolled in the MaPPS Trial at enrolment (n=10,676)

**Possible determinants**	**n (%)**	**BMI categories**							
		**Crude**				**Multivariable adjusted**			
		Underweight	*P*	Overweight/ obese	*P*	Underweight	*P*	Overweight/ obese	*P*
Level 1: Structural factors – socioeconomic status									
Occupation									
Unskilled manual labour	1,837 (17.2)	(reference)	-	(reference)	-	(reference)	-	(reference)	-
Skilled manual labour	2,372 (22.2)	0.81 (0.71 to 0.93)	0.002	2.77 (2.17 to 3.55)	<0.001	0.95 (0.83 to 1.09)	0.48	1.91 (1.48 to 2.48)	<0.001
Within the home	5,779 (54.1)	0.75 (0.67 to 0.84)	<0.001	3.52 (2.81 to 4.42)	<0.001	0.93 (0.83 to 1.05)	0.25	2.07 (1.61 to 2.67)	<0.001
Other[2]	688 (6.4)	0.76 (0.62 to 0.93)	0.007	3.80 (2.83 to 5.11)	<0.001	1.11 (0.83 to 1.49)	0.47	1.64 (1.19 to 2.26)	0.002
Religion									
Non-Muslim	919 (8.6)	(reference)	-	(reference)	-	(reference)	-	(reference)	-
Muslim	9,757 (91.4)	0.64 (0.56 to 0.74)	<0.001	1.74 (1.35 to 2.25)	<0.001	0.76 (0.61 to 0.93)	0.01	0.98 (0.71 to 1.36)	0.92
Wealth quintile									
Poorest	1,883 (17.6)	(reference)	-	(reference)	-	(reference)	-	(reference)	-
Poor	1,972 (18.5)	0.81 (0.71 to 0.92)	0.002	1.98 (1.48 to 2.64)	<0.001	0.87 (0.74 to 1.02)	0.09	1.81 (1.36 to 2.43)	<0.001
Middle	2,114 (19.8)	0.73 (0.64 to 0.83)	<0.001	3.31 (2.52 to 4.34)	<0.001	0.79 (0.70 to 0.89)	<0.001	2.84 (2.02 to 4.00)	<0.001
Rich	2,215 (20.7)	0.62 (0.54 to 0.71)	<0.001	4.68 (3.60 to 6.09)	<0.001	0.68 (0.59 to 0.78)	<0.001	3.88 (2.85 to 5.28)	<0.001
Richest	2,492 (23.3)	0.55 (0.48 to 0.63)	<0.001	6.36 (4.92 to 8.22)	<0.001	0.59 (0.48 to 0.72)	<0.001	5.24 (3.93 to 6.98)	<0.001
Level 2: Intermediary factors - household and personal characteristics									
Marital status									
Married	4,267 (40.0)	(reference)	-	(reference)	-	(reference)	-	(reference)	-
Unmarried	6,409 (60.0)	1.14 (1.04 to 1.24)	0.004	0.90 (0.81 to 1.01)	0.08	1.25 (1.11 to 1.42)	<0.001	0.77 (0.69 to 0.85)	<0.001
Perception of own health									
Poor or fair	1,275 (11.9)	(reference)	-	(reference)	-	(reference)	-	(reference)	-
Good	6,828 (64.0)	0.78 (0.68 to 0.89)	<0.001	1.18 (0.97 to 1.44)	0.09	0.78 (0.69 to 0.88)	<0.001	1.16 (0.94 to 1.44)	0.18
Excellent	2,573 (24.1)	0.75 (0.64 to 0.87)	<0.001	1.35 (1.09 to 1.67)	0.006	0.78 (0.69 to 0.89)	<0.001	1.16 (0.89 to 1.54)	0.27
Decision-making autonomy									
All decisions made by family	4,365 (40.9)	(reference)	-	(reference)	-	(reference)	-	(reference)	-
Most decisions made by family	3,630 (34.0)	0.91 (0.83 to 1.01)	0.08	1.08 (0.95 to 1.24)	0.23	0.92 (0.85 to 0.99)	0.03	1.08 (0.93 to 1.27)	0.31
Decisions made jointly with family or autonomously	2,681 (25.1)	0.85 (0.76 to 0.94)	0.003	1.19 (1.04 to 1.37)	0.01	0.85 (0.77 to 0.95)	0.003	1.24 (1.06 to 1.45)	0.01

[1]Values are n (%) or RR (95% CI)
[2]Other category included those who identified themselves as a student (526[4.9%]) or professional (688[6.4%])

For peer review only

Table 4. Comparison of social determinants of health in hierarchical model of stunting (height <145 cm) among late adolescent girls (n=14,771) and adult women (n=10,676) enrolled in the MaPPS Trial at enrolment

**Possible determinants**	**Stunting among late adolescent girls**				**Stunting among young women**			
	Crude		Multivariable adjusted		Crude		Multivariable adjusted	
	OR (95% CI)	*P*	OR (95% CI)	*P*	OR (95% CI)	*P*	OR (95% CI)	*P*
Level 1: Structural factors – socioeconomic status								
Highest level of education								
None	(reference)	-	(reference)	-	(reference)	-	(reference)	-
Primary	0.65 (0.57 to 0.74)	<0.001	0.76 (0.62 to 0.94)	0.01	0.62 (0.51 to 0.74)	<0.001	0.76 (0.63 to 0.93)	0.006
Secondary or higher	0.33 (0.29 to 0.38)	<0.001	0.48 (0.39 to 0.59)	<0.001	0.51 (0.43 to 0.61)	<0.001	0.79 (0.66 to 0.95)	0.01
Wealth quintile								
Poorest	(reference)	-	(reference)	-	(reference)	-	(reference)	-
Poor	0.83 (0.72 to 0.97)	0.02	0.91 (0.77 to 1.08)	0.29	0.88 (0.72 to 1.08)	0.22	0.92 (0.73 to 1.17)	0.52
Middle	0.67 (0.57 to 0.78)	<0.001	0.79 (0.65 to 0.96)	0.02	0.65 (0.52 to 0.80)	<0.001	0.70 (0.59 to 0.84)	<0.001
Rich	0.46 (0.39 to 0.55)	<0.001	0.63 (0.49 to 0.81)	<0.001	0.48 (0.38 to 0.60)	<0.001	0.54 (0.40 to 0.74)	<0.001
Richest	0.26 (0.21 to 0.32)	<0.001	0.42 (0.35 to 0.51)	<0.001	0.34 (0.27 to 0.43)	<0.001	0.41 (0.29 to 0.57)	<0.001
Level 2: Intermediary factors - household and personal characteristics								
Ever been pregnant								
Yes	(reference)	-	-	-	(reference)	-	(reference)	-
No	0.97 (0.74 to 1.27)	0.81	-	-	1.13 (0.96 to 1.32)	0.15	1.27 (1.10 to 1.47)	0.001
Household food security status								
Food insecure	(reference)	-	(reference)	-	(reference)	-	(reference)	-
Food secure	0.58 (0.51 to 0.64)	<0.001	0.77 (0.66 to 0.90)	0.001	0.57 (0.49 to 0.66)	<0.001	0.73 (0.59 to 0.90)	0.003
Level 4: Intermediary factors - actions and practices-related characteristics								
Self-efficacy								
Low	(reference)	-	(reference)		(reference)	-	-	-
Moderate	0.62 (0.56 to 0.70)	<0.001	0.74 (0.64 to 0.84)	<0.001	0.83 (0.71 to 0.98)	0.03	-	-
High	0.59 (0.50 to 0.71)	<0.001	0.74 (0.62 to 0.87)	<0.001	0.78 (0.63 to 0.97)	0.03	-	-
Decision-making autonomy								
All decisions made by family	(reference)	-	(reference)	-	(reference)	-	-	-
Most decisions made by family	0.83 (0.74 to 0.94)	0.003	0.88 (0.76 to 1.01)	0.08	0.99 (0.84 to 1.16)	0.90	-	-
Decisions made jointly with family or autonomously	0.74 (0.62 to 0.89)	0.001	0.76 (0.62 to 0.92)	0.01	0.85 (0.71 to 1.02)	0.09	-	-

For peer review only

DISCUSSION

Using enrolment data from a large trial conducted in rural Pakistan, we found high levels of undernutrition among late adolescent and young women 15–23 y using anthropometric indicators. Our study offers the most comprehensive analysis of the determinants of malnutrition among late adolescent girls and young women in rural Pakistan. Across the multivariable models for acute and chronic measures of undernutrition, those who were impoverished were at increased risk regardless of age group. This suggests that structural factors play an important, underlying role in female nutrition in this setting. That an emerging burden of overweight/obesity was also observed in this rural population indicates that interventions are needed to address both ends of the malnutrition spectrum. To the best of our knowledge, this is the largest survey of a cohort that includes both unmarried and married Pakistani late adolescent girls and young women.

Pakistan is known to have a high burden of malnutrition, with a long term impact on human capital and costing an estimated 7.6 billion USD/year.[35] We found the mean BMI among study participants was lower than the PDHS national average (20.2 kg/m^2 versus 25.7 kg/m^2).[17] This likely reflects that the PDHS only surveyed ever-married WRA (15–49 y). In the recently completed 2018 Pakistan National Nutrition Survey, 22.6%, 19.8%, and 10.2% of WRA in Sindh province were found to be underweight, overweight, and obese, respectively.[36] Notably, both the PDHS and National Nutrition Survey include urban settings, where the risk of overweight and obesity is higher.[37]

Of the limited adolescent nutrition studies available globally, wealth has consistently been found to be a determinant of undernutrition in multiple settings.[38-42] Regardless of whether someone

For peer review only - http://bmjopen.bmj.com/site/about/guidelines.xhtml

was an adolescent or young women, we found that at least one structural SDoH variable tied to poverty was associated with an increased risk of undernutrition outcomes. Notably, food insecurity was maintained in several models. At least one variable considered a proxy for empowerment was maintained in each adolescent model, with greater empowerment being associated with an improvement in nutritional status. Considered a blend of resources (materials that allow someone to make her goals a reality), agency (the ability to define and act upon someone's goals), and achievement (obtaining a desired goal), empowerment enables someone to make decisions to take charge of her own life and affect her personal circumstances.[43] Empowered women are widely recognized to be better able to promote their own health and well-being; whereas, lacking empowerment is suggested to present a further barriers to achieving optimal nutritional status.[3]

We had hypothesized that married late adolescent and young women might be at greater risk for undernutrition because early marriage has been suggested to increase nutritional vulnerability;[44] however, in the hierarchical models for both adolescent and young women, being married was associated with increased likelihood of being in a higher BMI category compared to those unmarried. This it is likely attributable to an increase in status within the household, as married participants contributed more to food-related decision-making (data not shown). An association between marital status and stunting, a chronic measure of poor nutrition across the lifespan, was not observed.

There is a recognized need in Pakistan to focus on the health and nutrition of adolescent girls,[45 46] and it is well documented that preconception health and well-being are important to the individual, as well as to her future offspring.[47] To address inequalities due to poverty, the use of

For peer review only - http://bmjopen.bmj.com/site/about/guidelines.xhtml

social safety nets could function as an important resource. As food insecurity prevalence was notable, provision of cash transfers, for example, could lend to the purchase of more nutritious food, although we are not certain at this time of the exact effect that this might have on an adolescent or young women's actual intake. However, economic status alone does not confer a healthy BMI.[42] We would note that there will need to be a concurrent emphasis on maintaining a healthy BMI, given that the prevalence of overweight/obesity in Pakistan has increased over time in both urban and rural settings, the existing double burden of malnutrition, and ongoing nutrition transition. To address structural and empowerment-related barriers, increased and coordinated efforts between the public health system and the education sector could have potential. The Government of Pakistan has recently released its nutritional strategy and operational plan for targeting adolescent malnutrition, which will be important to implementing legislation on adolescent nutrition.[48]

Limitations of this study include that our catchment area was restricted to the areas of Matiari District covered by the LHW Programme. Furthermore, our findings are specific to the context in rural Pakistan, and findings may not reflect settings with different cultural norms or burdens of undernutrition, as seen in Africa and South America.[49] While BMI is a globally accepted index of nutritional status, the relationship between BMI and body composition can be controversial, as BMI does not account for excess weight due to adiposity versus muscularity, edema, or bone mass.[6] It would have been informative to have had additional measures of body composition given its influence on health. We additionally acknowledge that the adolescent period does commence at 10 y, although given the aims of the MaPPS Trial we did not enrol adolescent girls 10–14 y. The larger study focused on adolescent and young women, not adolescent boys or young men; yet, males are also important given their earning potential and contribution to the

For peer review only

economy.[46] A further similar evaluation of this group would be informative, and aid understanding gendered cultural norms. Although the assessment of macro-level SDoH (e.g., governance, policies) was beyond the scope of this assessment, the importance of structural SDoH to all models suggests that underlying factors that create divisions within society are precarious. In their cross-sectional assessment of the factors affecting thinness among adolescent girls 10–19 y in low and middle income countries, Candler et al[38] found that macroeconomic factors contribute to the genesis of adolescent thinness, and that reducing adolescent thinness should include addressing national food security and economic development.

CONCLUSIONS

Given the findings of this study, and the concerning status of malnutrition in Pakistan, commitment to improving the nutrition of adolescent and young women in Pakistan is critical and should include those unmarried. Reaching these individuals is important not only because of their reproductive potential, but also given their right to optimal nutrition and well-being.[50] To target undernutrition, particularly, there is a need for both direct and indirect nutrition and empowerment interventions that are integrated and multi-sectoral,[51] as highlighted in the recent national adolescent nutrition policy recommendations.[48] An ideal program for adolescent and young women should be inclusive and accommodating, reach those who are most vulnerable, and account for cultural- and gender-relevant factors.[52]

For peer review only

Acknowledgements: The authors wish to acknowledge the participants in the MaPPS Trial and the field research team. We are grateful for the ongoing data-related assistance and support provided by Rasool Bux, Arjumand Rizvi, and the members of the Data Management Unit at Aga Khan University.

Contributions: JBB and ZAB designed research; YW and SBS conducted research; IA and AH performed data management; JBB analyzed data; JBB wrote first manuscript draft; ZAB, YW, SBS, IA, and AH provided manuscript feedback; ZAB has primary responsibility for final content. All authors reviewed this manuscript and approved the final version.

Funding: The MaPPS Trial was supported by the Bill and Melinda Gates Foundation (grant number: OPP1148892) and the World Food Programme (grant number: HQ15NF493 - CTR). JBB received research scholarships from the Vanier Canada Graduate Scholarship; Hospital for Sick Children Foundation Student Scholarship Program; and the Province of Ontario.

Competing interests: None declared.

Ethical approval: Ethical review for this study was obtained from the Aga Khan University Ethics Review Committee (Protocol #4324-Ped-ERC-16) and the Hospital for Sick Children Research Ethics Board (Protocol #1000054682).

Data availability statement: The anonymized, individual-level data from this cross-sectional assessment, situated within a clinical trial, will be made available upon reasonable request from the corresponding author.

For peer review only

REFERENCES

1. World Health Organization. Guideline: implementing effective actions for improving adolescent nutrition. Geneva: World Health Organization, 2018.

2. Sawyer SM, Afifi RA, Bearinger LH, et al. Adolescence: a foundation for future health. *Lancet* 2012;379(9826):1630-40.

3. Christian P, Smith ER. Adolescent Undernutrition: Global Burden, Physiology, and Nutritional Risks. *Ann Nutr Metab* 2018;72(4):316-28.

4. Norris SA, Frongillo EA, Black MM, et al. Nutrition in adolescent growth and development. *Lancet* 2022;399(10320):172-84. doi: 10.1016/s0140-6736(21)01590-7 [published Online First: 2021/12/03]

5. Aguayo VM, Paintal K. Nutrition in adolescent girls in South Asia. *BMJ* 2017;357:j1309. doi: 10.1136/bmj.j1309 [published Online First: 2017/04/13]

6. Gibson R. Principles of Nutritional Assessment. New York: Oxford University Press 2005.

7. Black RE, Victora CG, Walker SP, et al. Maternal and child undernutrition and overweight in low-income and middle-income countries. *Lancet* 2013;382(9890):427-51.

8. Twig G, Yaniv G, Levine H, et al. Body mass index in 2.3 million adolescents and cardiovascular death in adulthood. *N Engl J Med* 2016;374:2430–40.

9. Saydah S, Bullard KM, Imperatore G, et al. Cardiometabolic risk factors among US adolescents and young adults and risk of early mortality. *Pediatrics* 2013;131:e679-86. .

10. Viner RM, Ozer EM, Denny S, et al. Adolescence and the social determinants of health. *Lancet* 2012;379(9826):1641-52.

11. Commission on Social Determinants of Health. Closing the gap in a generation: health equity through action on the social determinants of health. Geneva: World Health Organization, 2008.

For peer review only

12. Fatusi AO, Bello B. Social Determinants of Health in Adolescent and Young Women's Health and Nutrition: Current Evidence. In: Bhutta ZA, Makarides M, Prentice AM, eds. Health and Nutrition in Adolescents and Young Women: Preparing for the Next Generation. Basel: Nestle Nutr Inst Workshop Ser 2015:61-69.

13. United Nations. Transforming Our World: The 2030 Agenda for Sustainable Development. New York: United Nations 2015.

14. Christian P, Smith E. Adolescent Undernutrition: Global Burden, Physiology, and Nutritional Risks. *Ann Nutr Metab* 2018;72(4):316-28.

15. Bhutta Z, Lassi Z, Bergeron G, et al. Delivering an action agenda for nutrition interventions addressing adolescent girls and young women: priorities for implementation and research. *Ann N Y Acad Sci* 2017;1393(1):61-71.

16. Ruel MT, Alderman H. Nutrition-sensitive interventions and programmes: how can they help to accelerate progress in improving maternal and child nutrition? *Lancet* 2013;382(9891):536-51. doi: 10.1016/s0140-6736(13)60843-0 [published Online First: 2013/06/12]

17. National Institute of Population Studies (NIPS) [Pakistan] and ICF. Pakistan Demographic and Health Survey 2017-18. Islamabad, Pakistan, and Rockville, Maryland, USA: NIPS and ICF, 2019.

18. Baxter JB, Wasan Y, Soofi SB, et al. Effect of life skills building education and micronutrient supplements provided from preconception versus the standard of care on low birth weight births among adolescent and young Pakistani women (15-24 years): a prospective, population-based cluster-randomized trial. *Reprod Health* 2018;15(1):104.

19. Centers for Disease Control and Prevention. National Health and Nutrition Examination Survey: anthropometry procedures manual. Hyattsville, MD: National Center for Health Statistics, Centers for Disease Control and Prevention, 2009.

20. Ulijaszek SJ, Kerr, D.A.,. Anthropometric measurement error and the assessment of nutritional status. *Br J Nutr* 1999;82:165–77.

For peer review only

21. World Health Organization. 2018 Global Reference List of 100 Core Health Indicators (plus health-related SDGs). Geneva: World Health Organization, 2018.

22. Baxter JB, Kortenaar JL, Wasan Y, et al. Age-Based Anthropometric Cutoffs Provide Inconsistent Estimates of Undernutrition: Findings from a Cross-Sectional Assessment of Late-Adolescent and Young Women in Rural Pakistan. *Curr Dev Nutr* 2021;5(11):nzab130. doi: 10.1093/cdn/nzab130 [published Online First: 2021/12/14]

23. Tumilowicz A, Beal T, Neufeld LM, et al. Perspective: Challenges in Use of Adolescent Anthropometry for Understanding the Burden of Malnutrition. *Adv Nutr* 2019;10(4):563-75.

24. de Onis M, Onyango AW, Borghi E, et al. Development of a WHO growth reference for school-aged children and adolescents. *Bull World Health Organ* 2007;85:660–67.

25. Baxter JB, Wasan Y, Hussain A, et al. Characterizing Micronutrient Status and Risk Factors among Late Adolescent and Young Women in Rural Pakistan: A Cross-Sectional Assessment of the MaPPS Trial. *Nutrients* 2021;13:1237.

26. UNICEF. The State of the World's Children [Internet]. New York: Oxford University Press 1998.

27. Solar O, Irwin A. A conceptual framework for action on the social determinants of health. Social Determinants of Health Discussion Paper 2 (Policy and Practice). Geneva: World Health Organization, 2010.

28. Lovibond SH, Lovibond PF. Manual for the Depression Anxiety Stress Scales. Sydney: Psychology Foundation, 1995.

29. Coates J, Swindale A, Bilinsky P. Household food insecurity access scale (HFIAS) for measurement of household food access: Indicator guide (v. 3). Washington: Food and Nutrition Technical Assistance Project, Academy for Educational Development, 2007.

30. Currie C, Inchley J, Molcho M, et al. Health Behaviour in School-aged Children (HBSC) Study Protocol: Background, Methodology, and Mandatory items for the 2013/14 Survey. St. Andrews: CAHRU, 2014.

For peer review only

31. Schwarzer R, Jerusalem M. Generalized self-efficacy scale. In: Weinman J WS, Johnston M, ed. Measures in health psychology: a user's portfolio Causal and control beliefs. Windsor: NFER-NELSON 1995:35-37.

32. Ewerling F, Lynch JW, Victora CG, et al. The SWPER index for women's empowerment in Africa: development and validation of an index based on survey data. *Lancet Glob Health* 2017;5(9):e916-e23.

33. Victora CG, Huttly SR, Fuchs SC, et al. The role of conceptual frameworks in epidemiological analysis: a hierarchical approach. *Int J Epidemiol* 1997;26:224-27.

34. Hosmer DW, Lemeshow S. Applied Logistic Regression. 2nd ed. New York: John Wiley 2000.

35. World Food Programme. The Economic Consequences of Undernutrition in Pakistan: An Assessment of Losses. Islamabad: World Food Programme Pakistan, 2017.

36. UNICEF Pakistan. National Nutrition Survey 2018: Key Findings Report. Islamabad: UNICEF Pakistan 2018.

37. Janjua NZ, Mahmood B, Bhatti JA, et al. Association of household and community socioeconomic position and urbanicity with underweight and overweight among women in Pakistan. *PLoS One* 2015;10(4):e0122314.

38. Candler T, Costa S, Heys M, et al. Prevalence of Thinness in Adolescent Girls in Low- and Middle-Income Countries and Associations With Wealth, Food Security, and Inequality. . *J Adolesc Health* 2017;60(4):447-54.

39. Harding KL, Aguayo VM, Webb P. Trends and Correlates of Overweight among Pre-School Age Children, Adolescent Girls, and Adult Women in South Asia: An Analysis of Data from Twelve National Surveys in Six Countries over Twenty Years. *Nutrients* 2019;11(8):1899.

40. Leroy JL, Ruel M, Sununtnasuk C, et al. Understanding the determinants of adolescent nutrition in Bangladesh. *Ann N Y Acad Sci* 2018

41. Radhika MS, Swetha B, Kumar NB, et al. Dietary and nondietary determinants of nutritional status among adolescent girls and adult women in India. *Ann N Y Acad Sci* 2018;1416(1):5-17.

For peer review only

42. Young MF, Nguyen P, Tran LM, et al. A Double Edged Sword? Improvements in Economic Conditions over a Decade in India Led to Declines in Undernutrition as Well as Increases in Overweight among Adolescents and Women. *J Nutr* 2020;150(2):364-72. doi: 10.1093/jn/nxz251 [published Online First: 2019/10/17]

43. Kabeer N. Resources, agency, achievements: reflections on the measurement of women's empowerment. *Dev Change* 1999;30:435-64.

44. Marphatia AA, Ambale GS, Reid AM. Women's Marriage Age Matters for Public Health: A Review of the Broader Health and Social Implications in South Asia. *Front Public Health* 2017;5:269. doi: 10.3389/fpubh.2017.00269 [published Online First: 2017/11/03]

45. Government of Pakistan. National Vision 2016-2025 for Coordinated Priority Actions to Address Challenges of Reproductive, Maternal, Newborn, Child, Adolescent Health and Nutrition. Islamabad: Government of Pakistan, 2015.

46. Government of Pakistan, World Health Organization. Adolescent Nutrition and Supplementation Guidelines for Pakistan. Islamabad: Government of Pakistan, 2019.

47. Dean SV, Lassi ZS, Imam AM, et al. Preconception care: nutritional risks and interventions. *Reprod Health* 2014;11(Suppl 3):S3.

48. Government of Pakistan. Pakistan Adolescent Nutrition Strategy and Operations Plan. Karachi: Government of Pakistan 2020.

49. Akseer N, Al-Gashm S, Mehta S, et al. Global and regional trends in the nutritional status of young people: a critical and neglected age group. *Ann N Y Acad Sci* 2017;1393(1):3-20.

50. Fox EL, Davis C, Downs SM, et al. Who is the Woman in Women's Nutrition? A Narrative Review of Evidence and Actions to Support Women's Nutrition throughout Life. *Current Developments in Nutrition* 2018;3(1) doi: 10.1093/cdn/nzy076

51. Keats E, Das JK, Salam RA, et al. Effective interventions to address maternal and child malnutrition: an update of the evidence. *Lancet Child Adolesc Health* 2021 doi: 10.1016/s2352-4642(20)30274-1 [published Online First: 2021/03/11]

For peer review only

52. Hargreaves D, Mates E, Menon P, et al. Strategies and interventions for healthy adolescent growth, nutrition, and development. *Lancet* 2022;399(10320):198-210. doi: 10.1016/s0140-6736(21)01593-2 [published Online First: 2021/12/03]

For peer review only

Supplementary Material

Table S1. Comparison of social determinants of health with BMI categories (underweight [<18.5 kg/m^2], normal weight [18.5–24.9 kg/m^2; base], and overweight and obese [≥25 kg/m^2]) among late adolescent girls (15–18.9 y) enrolled in the MaPPS Trial at enrolment (n=14,771)[1]

Possible determinants	n (%)	BMI categories: Crude – Underweight	*P*	Crude – Overweight/ obese	*P*	Multivariable adjusted – Underweight	*P*	Multivariable adjusted – Overweight/ obese	*P*
Level 1: Structural factors – socioeconomic status									
Highest level of education									
None	6,297 (42.6)	(reference)	-	(reference)	-	-	-	-	-
Primary	3,652 (24.7)	0.96 (0.89 to 1.05)	0.39	1.46 (1.23 to 1.74)	<0.001	-	-	-	-
Secondary or higher	4,822 (32.6)	0.86 (0.80 to 0.93)	<0.001	2.05 (1.76 to 2.38)	<0.001	-	-	-	-
Occupation									
Unskilled manual labour	2,622 (17.8)	(reference)	-	(reference)	-	(reference)	-	(reference)	-
Skilled manual labour	2,762 (18.7)	0.85 (0.76 to 0.95)	0.004	2.44 (1.83 to 3.25)	<0.001	0.92 (0.79 to 1.07)	0.26	1.83 (1.42 to 2.36)	<0.001
Within the home	6,351 (43.0)	0.83 (0.75 to 0.91)	<0.001	3.28 (2.54 to 4.25)	<0.001	0.94 (0.83 to 1.07)	0.34	2.03 (1.61 to 2.58)	<0.001
Other[2]	3,036 (20.6)	0.90 (0.81 to 1.00)	0.06	3.26 (2.48 to 4.30)	<0.001	1.14 (0.94 to 1.37)	0.18	1.44 (1.09 to 1.92)	0.01
Religion									
Non-Muslim	1,483 (10.0)	(reference)	-	(reference)	-	-	-	-	-
Muslim	13,288 (90.0)	0.79 (0.71 to 0.88)	<0.001	1.96 (1.48 to 2.61)	<0.001	-	-	-	-
Wealth quintile									
Poorest	2,666 (18.0)	(reference)	-	(reference)	-	(reference)	-	(reference)	-
Poor	2,881 (19.5)	0.84 (0.76 to 0.94)	0.002	1.47 (1.07 to 2.01)	0.001	0.84 (0.74 to 0.95)	0.01	1.34 (1.07 to 1.67)	0.01
Middle	3,025 (20.5)	0.79 (0.71 to 0.88)	<0.001	2.36 (1.77 to 3.15)	<0.001	0.78 (0.68 to 0.89)	<0.001	2.07 (1.63 to 2.63)	<0.001
Rich	3,145 (21.3)	0.71 (0.64 to 0.79)	<0.001	3.42 (2.59 to 4.51)	<0.001	0.69 (0.58 to 0.82)	<0.001	2.94 (2.32 to 3.74)	<0.001
Richest	3,054 (20.7)	0.69 (0.62 to 0.77)	<0.001	5.18 (3.96 to 6.79)	<0.001	0.65 (0.56 to 0.74)	<0.001	4.65 (3.49 to 6.20)	<0.001
Level 2: Intermediary factors - household and personal characteristics									
Marital status									
Married	1,418 (9.6)	(reference)		(reference)		(reference)	-	(reference)	-
Unmarried	13,353 (90.4)	1.38 (1.23 to 1.56)	<0.001	0.80 (0.66 to 0.97)	<0.03	1.42 (1.28 to 1.59)	<0.001	0.71 (0.59 to 0.85)	<0.001
Ever been pregnant									
Yes	572 (3.9)	(reference)	-	(reference)	-	-	-	-	-
No	14,199 (96.1)	1.14 (0.96 to 1.36)	0.14	0.80 (0.59 to 1.08)	0.14	-	-	-	-
Household food security status									
Food insecure	3,861 (26.1)	(reference)		(reference)		(reference)	-	(reference)	-
Food secure	10,910 (73.9)	0.83 (0.77 to 0.90)	<0.001	1.92 (1.61 to 2.28)	<0.001	0.91 (0.83 to 0.99)	0.04	1.24 (1.03 to 1.51)	0.03
Level 3: Intermediary factors - health and well-being characteristics									
Perception of own health									
Poor or fair	1,533 (10.4)	(reference)		(reference)		(reference)	-	(reference)	-

For peer review only

Possible determinants	n (%)	BMI categories: Crude: Underweight	P	Overweight/ obese	P	Multivariable adjusted: Underweight	P	Overweight/ obese	P
Good	9,731 (65.9)	0.82 (0.74 to 0.92)	0.001	1.23 (0.97 to 1.57)	0.09	0.83 (0.70 to 0.97)	0.03	1.23 (0.89 to 1.68)	0.21
Excellent	3,507 (23.7)	0.71 (0.63 to 0.80)	<0.001	1.40 (1.08 to 1.81)	0.01	0.74 (0.62 to 0.88)	0.001	1.22 (0.93 to 1.60)	0.16
Experience of depression-like feelings									
Severe or extremely severe	687 (4.7)	(reference)	-	(reference)	-	-	-	-	-
Moderate	1,545 (10.5)	0.93 (0.78 to 1.12)	0.47	1.16 (0.78 to 1.71)	0.46	-	-	-	-
Mild	1,236 (8.4)	0.94 (0.78 to 1.14)	0.53	1.15 (0.76 to 1.72)	0.51	-	-	-	-
None	11,303 (76.5)	0.78 (0.67 to 0.92)	0.002	1.20 (0.85 to 1.68)	0.30	-	-	-	-
Experience of anxiety-like feelings									
Severe or extremely severe	1,893 (12.8)	(reference)	-	(reference)	-	-	-	-	-
Moderate	2,124 (14.4)	1.02 (0.90 to 1.16)	0.78	0.96 (0.74 to 1.25)	0.77	-	-	-	-
Mild	952 (6.4)	0.85 (0.72 to 1.00)	0.05	1.06 (0.77 to 1.44)	0.73	-	-	-	-
None	9,802 (66.4)	0.79 (0.72 to 0.88)	<0.001	1.04 (0.85 to 1.27)	0.72	-	-	-	-
Experience of stress-like feelings									
Severe or extremely severe	491 (3.3)	(reference)	-	(reference)	-	-	-	-	-
Moderate	778 (5.3)	0.94 (0.75 to 1.19)	0.61	0.88 (0.54 to 1.41)	0.59	-	-	-	-
Mild	885 (6.0)	0.91 (0.72 to 1.14)	0.40	1.07 (0.68 to 1.68)	0.78	-	-	-	-
None	12,617 (85.4)	0.80 (0.67 to 0.97)	0.02	1.03 (0.70 to 1.49)	0.89	-	-	-	-
Level 4: Intermediary factors - actions and practices-related characteristics									
Self-efficacy									
Low	5,695 (38.6)	(reference)	-	(reference)	-	(reference)	-	(reference)	-
Moderate	7,161 (48.5)	0.85 (0.79 to 0.92)	<0.001	1.27 (1.10 to 1.46)	0.001	0.91 (0.85 to 0.97)	<0.001	1.09 (0.93 to 1.27)	0.29
High	1,915 (13.0)	0.75 (0.67 to 0.83)	<0.001	1.12 (0.91 to 1.37)	0.29	0.82 (0.72 to 0.92)	<0.001	0.91 (0.73 to 1.14)	0.41
Decision-making autonomy									
All decisions made by family	8,244 (55.8)	(reference)	-	(reference)	-	-	-	-	-
Most decisions made by family	4,702 (31.8)	0.9 (0.84 to 0.97)	0.008	1.18 (1.03 to 1.36)	0.02	-	-	-	-
Decisions made jointly with family or autonomously	1,825 (12.4)	0.84 (0.76 to 0.94)	0.002	1.32 (1.10 to 1.60)	0.004	-	-	-	-
Skipping breakfast									
Skips breakfast	4,479 (30.3)	(reference)	-	(reference)	-	-	-	-	-
Eats breakfast	10,292 (69.7)	0.99 (0.92 to 1.07)	0.81	1.18 (1.02 to 1.36)	0.03	-	-	-	-
Eating dinner with family									
Never	4,021 (27.2)	(reference)	-	(reference)	-	-	-	-	-
Sometimes	1,869 (12.7)	0.99 (0.89 to 1.12)	0.93	1.12 (0.90 to 1.40)	0.30	-	-	-	-
Everyday	8,881 (60.1)	0.95 (0.88 to 1.03)	0.23	1.13 (0.97 to 1.32)	0.11	-	-	-	-

[1]Values are n (%) or RR (95% CI)
[2]Other category included those who identified themselves as students (2,988[20.2%]) or professionals (48[0.3%])

For peer review only

Table S2. Comparison of social determinants of health with BMI categories (underweight [<18.5 kg/m^2], normal weight [18.5–24.9 kg/m^2; base], and overweight and obese [≥25 kg/m^2]) among young women (19–23 y) enrolled in the MaPPS Trial at enrolment (n=10,676)

		BMI categories							
		Crude				Multivariable adjusted			
**Possible determinants**	**n (%)**	Underweight	*P*	Overweight/ obese	*P*	Underweight	*P*	Overweight/ obese	*P*
Level 1: Structural factors – socioeconomic status									
Highest level of education									
None	5,087 (47.6)	(reference)	-	(reference)	-	-	-	-	-
Primary	2,411 (22.6)	0.80 (0.71 to 0.89)	<0.001	1.71 (1.47 to 1.98)	<0.001	-	-	-	-
Secondary or higher	3,178 (29.8)	0.74 (0.66 to 0.82)	<0.001	2.21 (1.94 to 2.52)	<0.001	-	-	-	-
Occupation									
Unskilled manual labour	1,837 (17.2)	(reference)	-	(reference)	-	(reference)	-	(reference)	-
Skilled manual labour	2,372 (22.2)	0.81 (0.71 to 0.93)	0.002	2.77 (2.17 to 3.55)	<0.001	0.95 (0.83 to 1.09)	0.48	1.91 (1.48 to 2.48)	<0.001
Within the home	5,779 (54.1)	0.75 (0.67 to 0.84)	<0.001	3.52 (2.81 to 4.42)	<0.001	0.93 (0.83 to 1.05)	0.25	2.07 (1.61 to 2.67)	<0.001
Other[2]	688 (6.4)	0.76 (0.62 to 0.93)	0.007	3.80 (2.83 to 5.11)	<0.001	1.11 (0.83 to 1.49)	0.47	1.64 (1.19 to 2.26)	0.002
Religion									
Non-Muslim	919 (8.6)	(reference)	-	(reference)	-	(reference)	-	(reference)	-
Muslim	9,757 (91.4)	0.64 (0.56 to 0.74)	<0.001	1.74 (1.35 to 2.25)	<0.001	0.76 (0.61 to 0.93)	0.01	0.98 (0.71 to 1.36)	0.92
Wealth quintile									
Poorest	1,883 (17.6)	(reference)	-	(reference)	-	(reference)	-	(reference)	-
Poor	1,972 (18.5)	0.81 (0.71 to 0.92)	0.002	1.98 (1.48 to 2.64)	<0.001	0.87 (0.74 to 1.02)	0.09	1.81 (1.36 to 2.43)	<0.001
Middle	2,114 (19.8)	0.73 (0.64 to 0.83)	<0.001	3.31 (2.52 to 4.34)	<0.001	0.79 (0.70 to 0.89)	<0.001	2.84 (2.02 to 4.00)	<0.001
Rich	2,215 (20.7)	0.62 (0.54 to 0.71)	<0.001	4.68 (3.60 to 6.09)	<0.001	0.68 (0.59 to 0.78)	<0.001	3.88 (2.85 to 5.28)	<0.001
Richest	2,492 (23.3)	0.55 (0.48 to 0.63)	<0.001	6.36 (4.92 to 8.22)	<0.001	0.59 (0.48 to 0.72)	<0.001	5.24 (3.93 to 6.98)	<0.001
Level 2: Intermediary factors - household and personal characteristics									
Marital status									
Married	4,267 (40.0)	(reference)	-	(reference)	-	(reference)	-	(reference)	-
Unmarried	6,409 (60.0)	1.14 (1.04 to 1.24)	0.004	0.90 (0.81 to 1.01)	0.08	1.25 (1.11 to 1.42)	<0.001	0.77 (0.69 to 0.85)	<0.001
Ever been pregnant									
Yes	2,906 (27.2)	(reference)	-	(reference)	-	-	-	-	-
No	7,770 (72.8)	1.03 (0.93 to 1.13)	0.58	0.93 (0.82 to 1.06)	0.28	-	-	-	-
Household food security status									
Food insecure	2,494 (23.4)	(reference)	-	(reference)	-	-	-	-	-
Food secure	8,182 (76.6)	0.79 (0.71 to 0.87)	<0.001	1.77 (1.52 to 2.07)	<0.001	-	-	-	-
Level 3: Intermediary factors - health and well-being characteristics									
Perception of own health									
Poor or fair	1,275 (11.9)	(reference)	-	(reference)	-	(reference)	-	(reference)	-
Good	6,828 (64.0)	0.78 (0.68 to 0.89)	<0.001	1.18 (0.97 to 1.44)	0.09	0.78 (0.69 to 0.88)	<0.001	1.16 (0.94 to 1.44)	0.18
Excellent	2,573 (24.1)	0.75 (0.64 to 0.87)	<0.001	1.35 (1.09 to 1.67)	0.006	0.78 (0.69 to 0.89)	<0.001	1.16 (0.89 to 1.54)	0.27
Experience of depression-like feelings									
Severe or extremely severe	537 (5.0)	(reference)	-	(reference)	-	-	-	-	-

For peer review only

Possible determinants	n (%)	BMI categories: Crude Underweight	P	Crude Overweight/ obese	P	Multivariable adjusted Underweight	P	Multivariable adjusted Overweight/ obese	P
Moderate	1,108 (10.4)	1.02 (0.82 to 1.28)	0.84	1.37 (0.97 to 1.93)	0.08	-	-	-	-
Mild	922 (8.6)	1.01 (0.8 to 1.28)	0.92	1.57 (1.11 to 2.23)	0.01	-	-	-	-
None	8,109 (76.0)	0.87 (0.72 to 1.06)	0.16	1.49 (1.11 to 2.01)	0.009	-	-	-	-
Experience of anxiety-like feelings									
Severe or extremely severe	1,421 (13.3)	(reference)	-	(reference)	-	-	-	-	-
Moderate	1,514 (14.2)	0.94 (0.80 to 1.10)	0.44	0.91 (0.73 to 1.15)	0.44	-	-	-	-
Mild	732 (6.9)	0.71 (0.58 to 0.87)	0.001	0.72 (0.54 to 0.97)	0.03	-	-	-	-
None	7,009 (65.7)	0.81 (0.72 to 0.92)	0.001	1.17 (0.98 to 1.40)	0.08	-	-	-	-
Experience of stress-like feelings									
Severe or extremely severe	405 (3.8)	(reference)	-	(reference)	-	-	-	-	-
Moderate	578 (5.4)	1.15 (0.87 to 1.52)	0.33	1.07 (0.72 to 1.59)	0.74	-	-	-	-
Mild	621 (5.8)	0.96 (0.72 to 1.26)	0.76	1.24 (0.85 to 1.80)	0.27	-	-	-	-
None	9,072 (85.0)	0.88 (0.71 to 1.10)	0.27	1.08 (0.79 to 1.47)	0.62	-	-	-	-
Level 4: Intermediary factors - actions and practices-related characteristics									
Self-efficacy									
Low	2,937 (27.5)	(reference)	-	(reference)	-	-	-	-	-
Moderate	5,709 (53.5)	0.92 (0.83 to 1.02)	0.10	1.34 (1.16 to 1.54)	<0.001	-	-	-	-
High	2,030 (19.0)	0.83 (0.73 to 0.95)	0.005	1.40 (1.18 to 1.66)	<0.001	-	-	-	-
Decision-making autonomy									
All decisions made by family	4,365 (40.9)	(reference)	-	(reference)	-	(reference)	-	(reference)	-
Most decisions made by family	3,630 (34.0)	0.91 (0.83 to 1.01)	0.08	1.08 (0.95 to 1.24)	0.23	0.92 (0.85 to 0.99)	0.03	1.08 (0.93 to 1.27)	0.31
Decisions made jointly with family or autonomously	2,681 (25.1)	0.85 (0.76 to 0.94)	0.003	1.19 (1.04 to 1.37)	0.01	0.85 (0.77 to 0.95)	0.003	1.24 (1.06 to 1.45)	0.01
Skipping breakfast									
Skips breakfast	3,130 (29.3)	(reference)	-	(reference)	-	-	-	-	-
Eats breakfast	7,546 (70.7)	0.93 (0.85 to 1.02)	0.11	1.15 (1.01 to 1.31)	0.03	-	-	-	-
Eating dinner with family									
Never	2,800 (26.2)	(reference)	-	(reference)	-	-	-	-	-
Sometimes	1,620 (15.2)	1.15 (1 to 1.32)	0.05	0.97 (0.81 to 1.17)	0.75	-	-	-	-
Everyday	6,256 (58.6)	1.13 (1.02 to 1.25)	0.02	1.08 (0.94 to 1.23)	0.27	-	-	-	-

[1]Values are n (%) or RR (95% CI)

[2]Other category included those who identified themselves as a student (526[4.9%]) or professional (688[6.4%])

For peer review only

Table S3. Comparison of social determinants of health with stunting (height <145 cm) among late adolescent girls (n=14,771) and adult women (n=10,676) enrolled in the MaPPS Trial at enrolment

Possible determinants	Stunting among late adolescent girls				Stunting among young women			
	Crude		Multivariable adjusted		Crude		Multivariable adjusted	
	OR (95% CI)	*P*	OR (95% CI)	*P*	OR (95% CI)	*P*	OR (95% CI)	*P*
Level 1: Structural factors – socioeconomic status								
Highest level of education								
None	(reference)	-	(reference)	-	(reference)	-	(reference)	-
Primary	0.65 (0.57 to 0.74)	<0.001	0.76 (0.62 to 0.94)	0.01	0.62 (0.51 to 0.74)	<0.001	0.76 (0.63 to 0.93)	0.006
Secondary or higher	0.33 (0.29 to 0.38)	<0.001	0.48 (0.39 to 0.59)	<0.001	0.51 (0.43 to 0.61)	<0.001	0.79 (0.66 to 0.95)	0.01
Occupation								
Unskilled manual labour	(reference)	-	-	-	(reference)	-	-	-
Skilled manual labour	0.56 (0.47 to 0.66)	<0.001	-	-	0.75 (0.61 to 0.91)	0.005	-	-
Within the home	0.69 (0.60 to 0.79)	<0.001	-	-	0.59 (0.49 to 0.71)	<0.001	-	-
Other[1]	0.32 (0.27 to 0.39)	<0.001	-	-	0.45 (0.32 to 0.65)	<0.001	-	-
Religion								
Non-Muslim	(reference)	-	-	-	(reference)	-	-	-
Muslim	0.57 (0.49 to 0.67)	<0.001	-	-	0.74 (0.59 to 0.92)	0.008	-	-
Wealth quintile								
Poorest	(reference)	-	(reference)	-	(reference)	-	(reference)	-
Poor	0.83 (0.72 to 0.97)	0.02	0.91 (0.77 to 1.08)	0.29	0.88 (0.72 to 1.08)	0.22	0.92 (0.73 to 1.17)	0.52
Middle	0.67 (0.57 to 0.78)	<0.001	0.79 (0.65 to 0.96)	0.02	0.65 (0.52 to 0.80)	<0.001	0.70 (0.59 to 0.84)	<0.001
Rich	0.46 (0.39 to 0.55)	<0.001	0.63 (0.49 to 0.81)	<0.001	0.48 (0.38 to 0.60)	<0.001	0.54 (0.40 to 0.74)	<0.001
Richest	0.26 (0.21 to 0.32)	<0.001	0.42 (0.35 to 0.51)	<0.001	0.34 (0.27 to 0.43)	<0.001	0.41 (0.29 to 0.57)	<0.001
Level 2: Intermediary factors - household and personal characteristics								
Marital status								
Married	(reference)	-	-	-	(reference)	-	-	-
Unmarried	0.89 (0.75 to 1.07)	0.21	-	-	0.99 (0.86 to 1.15)	0.93	-	-
Ever been pregnant								
Yes	(reference)	-	-	-	(reference)	-	(reference)	-
No	0.97 (0.74 to 1.27)	0.81	-	-	1.13 (0.96 to 1.32)	0.15	1.27 (1.10 to 1.47)	0.001
Household food security status								
Food insecure	(reference)	-	(reference)	-	(reference)	-	(reference)	-
Food secure	0.58 (0.51 to 0.64)	<0.001	0.77 (0.66 to 0.90)	0.001	0.57 (0.49 to 0.66)	<0.001	0.73 (0.59 to 0.90)	0.003
Level 3: Intermediary factors - health and well-being characteristics								
Perception of own health								
Poor or fair	(reference)	-	-	-	(reference)	-	-	-
Good	0.93 (0.78 to 1.10)	0.38	-	-	0.83 (0.67 to 1.02)	0.07	-	-
Excellent	0.85 (0.70 to 1.04)	0.11	-	-	0.79 (0.62 to 1.00)	0.06	-	-
Experience of depression-like feelings								
Severe or extremely severe	(reference)	-	-	-	(reference)	-	-	-
Moderate	0.93 (0.70 to 1.23)	0.61	-	-	0.98 (0.68 to 1.41)	0.90	-	-
Mild	0.94 (0.71 to 1.26)	0.70	-	-	0.86 (0.58 to 1.26)	0.43	-	-

For peer review only

**Possible determinants**	**Stunting among late adolescent girls**				**Stunting among young women**			
	Crude		Multivariable adjusted		Crude		Multivariable adjusted	
	OR (95% CI)	*P*	OR (95% CI)	*P*	OR (95% CI)	*P*	OR (95% CI)	*P*
None	0.79 (0.62 to 1.00)	0.05	-	-	0.88 (0.64 to 1.19)	0.40	-	-
Experience of anxiety-like feelings								
Severe or extremely severe	(reference)	-	-	-	(reference)	-	-	-
Moderate	0.84 (0.69 to 1.03)	0.09	-	-	0.82 (0.62 to 1.07)	0.14	-	-
Mild	0.96 (0.75 to 1.23)	0.76	-	-	0.99 (0.72 to 1.37)	0.97	-	-
None	0.83 (0.71 to 0.97)	0.02	-	-	0.94 (0.77 to 1.16)	0.56	-	-
Experience of stress-like feelings								
Severe or extremely severe	(reference)	-	-	-	(reference)	-	-	-
Moderate	0.93 (0.64 to 1.34)	0.69	-	-	0.93 (0.58 to 1.49)	0.76	-	-
Mild	1.09 (0.77 to 1.54)	0.63	-	-	0.78 (0.48 to 1.25)	0.30	-	-
None	0.90 (0.68 to 1.20)	0.49	-	-	0.98 (0.68 to 1.40)	0.90	-	-
Level 4: Intermediary factors - actions and practices-related characteristics								
Self-efficacy								
Low	(reference)	-	(reference)		(reference)	-	-	-
Moderate	0.62 (0.56 to 0.70)	<0.001	0.74 (0.64 to 0.84)	<0.001	0.83 (0.71 to 0.98)	0.03	-	-
High	0.59 (0.50 to 0.71)	<0.001	0.74 (0.62 to 0.87)	<0.001	0.78 (0.63 to 0.97)	0.03	-	-
Decision-making autonomy								
All decisions made by family	(reference)	-	(reference)	-	(reference)	-	-	-
Most decisions made by family	0.83 (0.74 to 0.94)	0.003	0.88 (0.76 to 1.01)	0.08	0.99 (0.84 to 1.16)	0.90	-	-
Decisions made jointly with family or autonomously	0.74 (0.62 to 0.89)	0.001	0.76 (0.62 to 0.92)	0.01	0.85 (0.71 to 1.02)	0.09	-	-
Skipping breakfast								
Skips breakfast	(reference)	-	-	-	(reference)	-	-	-
Eats breakfast	0.82 (0.73 to 0.91)	<0.001	-	-	0.79 (0.68 to 0.91)	0.002	-	-
Eating dinner with family								
Never	(reference)		-	-	(reference)	-	-	-
Sometimes	1.09 (0.92 to 1.30)	0.33	-	-	1.19 (0.95 to 1.49)	0.12	-	-
Everyday	0.93 (0.82 to 1.05)	0.23	-	-	1.02 (0.87 to 1.21)	0.79	-	-

[1]Other category included those who identified themselves as a student or profession

For peer review only

Table 1. STROBE-nut: An extension of the STROBE statement for nutritional epidemiology

Lachat C et al. (2016) STrengthening the Reporting of OBservational studies in Epidemiology – Nutritional Epidemiology (STROBE-nut): an extension of the STROBE statement. Plos Medicine 13(6) http://dx.doi.org/10.1371/journal.pmed.1002036 pdf or online version.

Item	Item nr	STROBE recommendations	Extension for Nutritional Epidemiology studies (STROBE-nut)	Reported on page #
**Title and abstract**	1	(a) Indicate the study's design with a commonly used term in the title or the abstract.<br>(b) Provide in the abstract an informative and balanced summary of what was done and what was found.	**nut-1** State the dietary/nutritional assessment method(s) used in the title, abstract, or keywords.	1-3
**Introduction**				
Background rationale	2	Explain the scientific background and rationale for the investigation being reported.		5-7
Objectives	3	State specific objectives, including any pre-specified hypotheses.		6-7
**Methods**				
Study design	4	Present key elements of study design early in the paper.		7
Settings	5	Describe the setting, locations, and relevant dates, including periods of recruitment, exposure, follow-up, and data collection.	**nut-5** Describe any characteristics of the study settings that might affect the dietary intake or	7

For peer review only

Item	Item nr	STROBE recommendations	Extension for Nutritional Epidemiology studies (STROBE-nut)	Reported on page #
			nutritional status of the participants, if applicable.	
Participants	6	a) Cohort study—Give the eligibility criteria, and the sources and methods of selection of participants. Describe methods of follow-up.<br><br>Case-control study—Give the eligibility criteria, and the sources and methods of case ascertainment and control selection. Give the rationale for the choice of cases and controls.<br><br>Cross-sectional study—Give the eligibility criteria, and the sources and methods of selection of participants.<br><br>(b) Cohort study—For matched studies, give matching criteria and number of exposed and unexposed.<br><br>Case-control study—For matched studies, give matching criteria and the number of controls per case.	**nut-6** Report particular dietary, physiological or nutritional characteristics that were considered when selecting the target population.	7-8
Variables	7	Clearly define all outcomes, exposures, predictors, potential confounders, and effect modifiers. Give diagnostic criteria, if applicable.	**nut-7.1** Clearly define foods, food groups, nutrients, or other food components.	8-10

For peer review only

For peer review only - http://bmjopen.bmj.com/site/about/guidelines.xhtml

Item	Item nr	STROBE recommendations	Extension for Nutritional Epidemiology studies (STROBE-nut)	Reported on page #
			**nut-7.2** When using dietary patterns or indices, describe the methods to obtain them and their nutritional properties.	
Data sources - measurements	8	For each variable of interest, give sources of data and details of methods of assessment (measurement).Describe comparability of assessment methods if there is more than one group.	**nut-8.1** Describe the dietary assessment method(s), e.g., portion size estimation, number of days and items recorded, how it was developed and administered, and how quality was assured. Report if and how supplement intake was assessed.<br><br>**nut-8.2** Describe and justify food composition data used. Explain the procedure to match food composition with consumption data. Describe the use of conversion factors, if applicable.<br><br>**nut-8.3** Describe the nutrient requirements, recommendations, or dietary guidelines and the evaluation approach used to compare intake with the dietary reference values, if applicable.<br><br>**nut-8.4** When using nutritional biomarkers, additionally use the STROBE Extension for Molecular Epidemiology (STROBE-ME). Report the type of biomarkers used and their usefulness as dietary exposure markers.	8-10

For peer review only

Item	Item nr	STROBE recommendations	Extension for Nutritional Epidemiology studies (STROBE-nut)	Reported on page #
			**nut-8.5** Describe the assessment of nondietary data (e.g., nutritional status and influencing factors) and timing of the assessment of these variables in relation to dietary assessment.<br><br>**nut-8.6** Report on the validity of the dietary or nutritional assessment methods and any internal or external validation used in the study, if applicable.	
Bias	9	Describe any efforts to address potential sources of bias.	**nut-9** Report how bias in dietary or nutritional assessment was addressed, e.g., misreporting, changes in habits as a result of being measured, or data imputation from other sources	8-10
Study Size	10	Explain how the study size was arrived at.		7
Quantitative variables	11	Explain how quantitative variables were handled in the analyses. If applicable, describe which groupings were chosen and why.	**nut-11** Explain categorization of dietary/nutritional data (e.g., use of N-tiles and handling of nonconsumers) and the choice of reference category, if applicable.	8-10
Statistical Methods	12	(a) Describe all statistical methods, including those used to control for confounding<br><br>(b) Describe any methods used to examine subgroups and interactions.	**nut-12.1** Describe any statistical method used to combine dietary or nutritional data, if applicable.	10-12

For peer review only

Item	Item nr	STROBE recommendations	Extension for Nutritional Epidemiology studies (STROBE-nut)	Reported on page #
		(c) Explain how missing data were addressed.<br><br>(d) Cohort study—If applicable, explain how loss to follow-up was addressed.<br><br>Case-control study—If applicable, explain how matching of cases and controls was addressed.<br><br>Cross-sectional study—If applicable, describe analytical methods taking account of sampling strategy.<br><br>(e) Describe any sensitivity analyses.	**nut-12.2** Describe and justify the method for energy adjustments, intake modeling, and use of weighting factors, if applicable.<br><br>**nut-12.3** Report any adjustments for measurement error, i.e,. from a validity or calibration study.	
**Results**				
Participants	13	(a) Report the numbers of individuals at each stage of the study—e.g., numbers potentially eligible, examined for eligibility, confirmed eligible, included in the study, completing follow-up, and analyzed.<br><br>(b) Give reasons for non-participation at each stage.<br><br>(c) Consider use of a flow diagram.	**nut-13** Report the number of individuals excluded based on missing, incomplete or implausible dietary/nutritional data.	12
Descriptive data	14	(a) Give characteristics of study participants (e.g., demographic, clinical, social) and	**nut-14** Give the distribution of participant characteristics across the exposure variables if applicable. Specify if food consumption of	12-13, Table 1

For peer review only

Item	Item nr	STROBE recommendations	Extension for Nutritional Epidemiology studies (STROBE-nut)	Reported on page #
		information on exposures and potential confounders<br><br>(b) Indicate the number of participants with missing data for each variable of interest<br><br>(c) Cohort study—Summarize follow-up time (e.g., average and total amount)	total population or consumers only were used to obtain results.	
Outcome data	15	Cohort study—Report numbers of outcome events or summary measures over time.<br><br>Case-control study—Report numbers in each exposure category, or summary measures of exposure.<br><br>Cross-sectional study—Report numbers of outcome events or summary measures.		14-16, Table 1
Main results	16	(a) Give unadjusted estimates and, if applicable, confounder-adjusted estimates and their precision (e.g., 95% confidence interval).<br><br>Make clear which confounders were adjusted for and why they were included.<br><br>(b) Report category boundaries when continuous variables were categorized.	**nut-16** Specify if nutrient intakes are reported with or without inclusion of dietary supplement intake, if applicable.	14-16, Tables 2-4

For peer review only - http://bmjopen.bmj.com/site/about/guidelines.xhtml

Item	Item nr	STROBE recommendations	Extension for Nutritional Epidemiology studies (STROBE-nut)	Reported on page #
		(c) If relevant, consider translating estimates of relative risk into absolute risk for a meaningful time period.		
Other analyses	17	Report other analyses done—e.g., analyses of subgroups and interactions and sensitivity analyses.	**nut-17** Report any sensitivity analysis (e.g., exclusion of misreporters or outliers) and data imputation, if applicable.	N/A
**Discussion**				
Key results	18	Summarize key results with reference to study objectives.		20
Limitation	19	Discuss limitations of the study, taking into account sources of potential bias or imprecision. Discuss both direction and magnitude of any potential bias.	**nut-19** Describe the main limitations of the data sources and assessment methods used and implications for the interpretation of the findings.	22-23
Interpretation	20	Give a cautious overall interpretation of results considering objectives, limitations, multiplicity of analyses, results from similar studies, and other relevant evidence.	**nut-20** Report the nutritional relevance of the findings, given the complexity of diet or nutrition as an exposure.	20-22
Generalizability	21	Discuss the generalizability (external validity) of the study results.		20-21
**Other information**				
Funding	22	Give the source of funding and the role of the funders for the present study and, if		24

For peer review only

Item	Item nr	STROBE recommendations	Extension for Nutritional Epidemiology studies (STROBE-nut)	Reported on page #
		applicable, for the original study on which the present article is based.		
Ethics			**nut-22.1** Describe the procedure for consent and study approval from ethics committee(s).	24
Supplementary material			**nut-22.2** Provide data collection tools and data as online material or explain how they can be accessed.	Yes

For peer review only

BMJ Open

Drivers of malnutrition among late adolescent and young women in rural Pakistan: A cross-sectional assessment of the MaPPS Trial

Journal:	*BMJ Open*
Manuscript ID	bmjopen-2022-063734.R2
Article Type:	Original research
Date Submitted by the Author:	13-Mar-2023
Complete List of Authors:	Baxter, Jo-Anna; The Hospital for Sick Children,<br>Wasan, Yaqub; Aga Khan University, Centre of Excellence in Women and Child Health<br>Hussain, Amjad; Aga Khan University, Pediatrics and Child Health<br>Soofi, Sajid; The Aga Khan University, Pediatrics & Child Health<br>Ahmed, Imran; Aga Khan University<br>Bhutta, Zulfiqar; The Aga Khan University, Division of Women and Child Health
**Primary Subject Heading**:	Global health
Secondary Subject Heading:	Nutrition and metabolism, Public health, Epidemiology
Keywords:	NUTRITION & DIETETICS, PUBLIC HEALTH, Community child health < PAEDIATRICS

SCHOLARONE™ Manuscripts

I, the Submitting Author has the right to grant and does grant on behalf of all authors of the Work (as defined in the below author licence), an exclusive licence and/or a non-exclusive licence for contributions from authors who are: i) UK Crown employees; ii) where BMJ has agreed a CC-BY licence shall apply, and/or iii) in accordance with the terms applicable for US Federal Government officers or employees acting as part of their official duties; on a worldwide, perpetual, irrevocable, royalty-free basis to BMJ Publishing Group Ltd ("BMJ") its licensees and where the relevant Journal is co-owned by BMJ to the co-owners of the Journal, to publish the Work in this journal and any other BMJ products and to exploit all rights, as set out in our licence.

The Submitting Author accepts and understands that any supply made under these terms is made by BMJ to the Submitting Author unless you are acting as an employee on behalf of your employer or a postgraduate student of an affiliated institution which is paying any applicable article publishing charge ("APC") for Open Access articles. Where the Submitting Author wishes to make the Work available on an Open Access basis (and intends to pay the relevant APC), the terms of reuse of such Open Access shall be governed by a Creative Commons licence – details of these licences and which Creative Commons licence will apply to this Work are set out in our licence referred to above.

Other than as permitted in any relevant BMJ Author's Self Archiving Policies, I confirm this Work has not been accepted for publication elsewhere, is not being considered for publication elsewhere and does not duplicate material already published. I confirm all authors consent to publication of this Work and authorise the granting of this licence.

For peer review only

TITLE PAGE

Title:

Drivers of malnutrition among late adolescent and young women in rural Pakistan: A cross-sectional assessment of the MaPPS Trial

Authors:

Jo-Anna B. Baxter[1,2], Yaqub Wasan[3], Amjad Hussain[3], Sajid B. Soofi[3], Imran Ahmed[3], Zulfiqar A. Bhutta[1-4]

Affiliations:

[1]Centre for Global Child Health, Hospital for Sick Children, Toronto, Canada

[2]Department of Nutritional Sciences, University of Toronto, Toronto, Canada

[3]Centre of Excellence in Women and Child Health, Aga Khan University, Karachi, Pakistan

[4] Dalla Lana School of Public Health, University of Toronto, Toronto, Canada

Corresponding author:

Dr. Zulfiqar A. Bhutta

Telephone number: +92 21 34930051

Email address: zulfiqar.bhutta@aku.edu

Postal address: Center of Excellence in Women and Child Health, Aga Khan University, Karachi 74800, Pakistan

Word count:

For peer review only

4041

Tables:

4

Figures:

0

Online supplemental material submitted:

Yes

ARTICLE SUMMARY

Abstract:

Objective: This study aimed to characterize the burden of malnutrition and assess how underlying determinants at the structural and intermediary levels contributed to malnutrition among late adolescent and young women in rural Pakistan.

Design: Cross-sectional enrolment data assessment.

Setting and participants: This study was conducted using data from adolescent and young women (n=25,447) enrolled in the Matiari emPowerment and Preconception Supplementation (MaPPS) Trial, collected from June 2017 to July 2018 in Matiari District, Pakistan. The WHO-based cut-offs were applied to anthropometric measures to estimate BMI categories

For peer review only

(underweight, overweight, obese) and stunting. Hierarchical models were generated to evaluate the association between the determinants with BMI categories and stunting among late adolescent girls and young women, respectively.

Primary and secondary outcome measures: The main outcomes of interest were BMI categories and stunting. Explanatory variables included measures of socioeconomic status, education, occupation, health, well-being, food security, empowerment, and food practices.

Results: Regardless of age group, the prevalence of underweight was high (36.9%; 95% CI: 36.3 to 37.5%). More late adolescent girls were underweight, while more young women were overweight/obese (P<0.001). Stunting affected 9.2% (95% CI: 8.9 to 9.6%) of participants, of which 35.7% were additionally underweight and 7.3% overweight/obese. Compared to those in the normal weight category, those underweight were more likely to be impoverished and less empowered. Those overweight/obese were more likely to be from a higher wealth quintile and food secure. Increased education level and food security were associated with reductions stunting risk.

Conclusions: This study informs the data gap and need for comprehensive research on adolescent nutritional status. Findings suggest factors related to poverty played an important, underlying role in undernutrition among participants. Commitment to improving the nutritional status of all adolescent and young women in Pakistan will be critical given the observed burden of malnutrition.

Trial registration: Clinicaltrials.gov NCT03287882.

Strengths and limitations of this study:

- Although the catchment area of this study was restricted to the areas covered by the LHW Programme, the study is very large and representative of rural setting in Pakistan.

For peer review only

- Given the cross-sectional design of the study, we cannot suggest causation.
- All anthropometric measurements (weight, height, middle upper arm circumference) were collected in duplicate following standardized procedures by trained study personnel, although we did not collect additional measures of body composition (e.g., skin-fold thickness, abdominal circumference).
- We used validated questionnaires to assess social determinants of health at both the structural and intermediary levels.

KEYWORDS

body mass index, stunting, adolescent girl, young woman, undernutrition, underweight, overweight, obesity

For peer review only

INTRODUCTION

Adolescence is a life stage characterized by substantial physical, cognitive, social, and emotional development, and nutritional status importantly underlies the transition from childhood to adulthood.[1] Defined by the World Health Organization (WHO) as the period from 10–19 y, some suggest adolescence extends to 24 y given continued cortical development.[1 2] Being malnourished during the adolescent period is associated with poor health outcomes throughout the life-course and has potential intergenerational effects.[3 4] In South Asia, undernutrition affects >38% of adolescent girls; there has been limited decline in the past decade and the increasing occurrence of overweight/obesity is of additional concern.[5]

Anthropometric measures can serve as a useful indicator for assessing malnutrition. Measures of undernutrition, including underweight (thinness) and stunting (short stature), are determined from body mass index (BMI) and attained height, respectively. While underweight reflects current nutritional status caused by insufficient or imbalanced intake and/or underlying disease, stunting reflects chronic or recurrent undernourishment.[6] Being underweight and/or stunted has been found to be associated with adverse health consequences to the individual, and, in the event of a pregnancy, increased risk for adverse pregnancy outcomes.[7] Conversely, overweight and obesity have been linked to the early onset of non-communicable diseases and premature mortality.[8 9]

Social determinants of health (SDoH) are described to influence nutritional inequalities, making understanding their role in malnutrition of importance.[10] SDoH are typically divided into two classes, structural and intermediary.[11] Structural SDoH underlie food availability and access, including stratifiers such as ethnicity, education, occupation, and income. Intermediary SDoH are

For peer review only

more proximal to nutrition-related outcomes, affecting material circumstances and biological, psychological, and behavioural factors.[12]

With the focus of the Sustainable Development Goals on equity, and the explicit aim in Goal 5 to empower all women and girls,[13] measuring, monitoring, and researching women's empowerment is of importance. Adolescent girls are an understudied population with respect to empowerment, and understanding the impact of interventions that aim to improve empowerment is a noted research gap.[1] Proxies for empowerment span SDoH at both the structural (e.g., education) and intermediary levels (e.g., self-efficacy and contribution to decision-making). They are suggested to influence nutritional status, although this association has predominantly been made between maternal empowerment and child nutritional status.[14-16] The WHO acknowledges that empowering adolescent girls, families, and communities is an important component to improving overall health and nutritional status.[1]

At this time, there are more young people than ever before in Pakistan, with around 30% of the population between 15-24 y.[17] Nationally representative surveys have traditionally focused on married women 15–49 y given the importance of reproductive outcomes, and estimate that the prevalence of underweight and stunting are high at 8.5 and 5.0%, respectively, and that there is an emerging burden of overweight and obesity.[17] However, there has been limited assessment among late adolescent girls and unmarried young women, thus there is a gap in information within this segment of the population. We aimed to assess the prevalence of malnutrition using anthropometric measures from all adolescent and young women (15–23 y) living in rural Pakistan, and determine whether structural and intermediary SDoH were associated with underweight, overweight/obese, and stunting. Understanding the diverse SDoH that predict

For peer review only

malnutrition will be informative to policy generation and the prioritization of programming to address current nutritional challenges.

METHODS

This study used cross-sectional data from late adolescent girls and young women (15−23 y) collected at enrolment within the Matiari emPowerment and Preconception Supplementation (MaPPS) Trial.

Setting and study design

The MaPPS Trial is a two-arm, cluster-randomized, controlled trial that aims to assess the impact of life skills building education and multiple micronutrient supplementation on several nutrition, health, and empowerment-related outcomes in rural Pakistan. The study methodology and data collection tools employed within this population-based effectiveness trial have been previously described.[18] The trial was registered at Clinicaltrials.gov as NCT03287882. The MaPPS Trial aimed to recruit approximately 25,400 non-pregnant adolescent and young women to observe enough pregnancies to detect a 25% relative reduction in low-birth-weight births (primary trial outcome), assuming prevalence of low birth weight of 30%, icc=0.011, k=0.16, accounting for 10% attrition, and probability of type I and type II errors of 0.05 and 0.20, respectively.

Participant enrolment

Participants were recruited to the MaPPS Trial from the end of June 2017 to July 2018, using a cluster-wise roll-out. The cluster unit was the catchment population of pre-identified health

For peer review only

facilities and their respective outreach community health workers (Lady Health Workers; LHWs) (n=26). Late adolescent girls and young women 15–23 y were identified from a household listing exercise conducted from December 2016 to May 2017, wherein all households covered by LHWs in a cluster unit were surveyed by study personnel. To screen for participation in the MaPPS Trial, late adolescent girls and young women 15–23 y were approached at their homes by study personnel to further assess their eligibility and ≥1 participant could be enrolled per household. Eligibility criteria included reporting being non-pregnant; physically able to comply with supplementation; not participating in any other nutrition studies; and intending to remain in the study area for the duration of the trial. The purpose and voluntary nature of the trial was verbally explained following confirmation of eligibility by study personnel and a written consent form was provided. Written documentation of informed consent was obtained as either a signature or a thumb impression, in the case that a participant was illiterate.

Patient and public involvement

Prior to the start of the household listing exercise, a wide range of activities were undertaken to obtain local approval of the MaPPS Trial. This included sharing information about what was being done within the MaPPS Trial and why with the district education department and health leaders (including the LHW Programme), and leaders from participating villages. The broader community and prospective participants were not invited to comment on the study design, and they were not consulted on the study outcomes or result interpretation for this research.

Data collection

Upon enrolment, a questionnaire was administered to collect information on participant demographics; socioeconomic status; reproductive health; general health, well-being, and

For peer review only

nutrition; and empowerment-related variables. Anthropometric (height, weight, middle-upper arm circumference) measurements were collected in duplicate by two trained data collectors using standardized methods.[19] Pre-set allowable differences were employed (height: <1.0 cm; weight: <0.5 kg), and a third measurement was collected if two measurements exceed this. In all analyses, the average of two acceptable measures was used. The technical error of measurement (TEM), relative TEM (%TEM), and coefficient of reliability (R) were calculated for height and weight.[20] Field monitoring teams additionally conducted regular data quality spot checks.[18] Data collectors were blinded to study arm allocation.

Outcomes of interest

The outcomes of interest in the present study were cut-offs for BMI (underweight: <18.5 kg/m^2, normal: 18.5–24.9 kg/m^2, overweight: 25–29.9 kg/m^2, obese: ≥30 kg/m^2) and stunting (height <145 cm).[21] BMI was considered categorically because of the increased health risks when it is too low or high. There are known issues with the adolescent-specific cut-offs for BMI and stunting (10–19 y),[22 23] thus we applied the WHO cut-offs for women of reproductive age (WRA; 15–49 y). This is common in international reporting given the age range overlap. Furthermore, the adult cut-offs are tied to reproductive outcomes and adolescent pregnancies were common in the study population.[17] For completeness, since the WHO Growth Reference is suggested to classify the growth of adolescents up to 19 y (228 completed months), we also applied the WHO igrowup package for Stata (StataCorp, College Station, TX) to adolescents' anthropometric data to determine height-for-age and BMI-for-age z-scores (HAZ and BAZ, respectively).[24]

Explanatory variables

For peer review only

Multiple explanatory variables were hypothesized to underlie the nutritional status outcomes of interest. The selection of underlying SDoH risk factors followed our previously published conceptual framework,[25] which was guided by the UNICEF framework on the causes of malnutrition[26] and the WHO framework on SDoH related to health and well-being.[27] These were split into two categories: structural (education, occupation, socioeconomic status, and religion) and intermediary (health, well-being, food insecurity, empowerment, and food practices).

Indicators for demographic information, socioeconomic status, and reproductive health were adapted from the Pakistan Demographic Health Survey (PDHS).[17] A variable for wealth quintile was derived using a principal component analysis from factors related to home characteristics and household asset ownership.[17] Other health-related proxies included self-reported health status and mental health (experience of depression, anxiety, and stress-related emotions over the preceding week; Depression, Anxiety, and Stress Score – 21 Items).[28] Other nutrition-related proxies included household food insecurity (Household Food Insecurity Access Scale)[29] and skipping breakfast and sharing dinner with family (Health Behaviour in School-aged Children Survey).[30] Empowerment proxies included generalized self-efficacy (Generalized Self-Efficacy Scale)[31] and decision-making autonomy (PDHS).[17] Self-efficacy scores were categorized into tertiles, corresponding to low, moderate, and high. A variable for decision-making autonomy was derived from reported participation in 5 decisions, adapted from the PDHS (decisions: own food consumption, own health care, household food purchases, household food distribution, household purchases). The scoring of decision-making participation was consistent with the SWPER index for WRA[32] and participants were categorized based on their involvement (all decisions made by family, most decisions made by family, decisions made jointly with family or autonomously).

For peer review only

Statistical analyses

Analyses were performed using Stata, version 15.0 (Stata Corporation). Since the enrolment data were collected prior to the study intervention, data were analyzed cross-sectionally, independent of study arm assignment. To describe the study population, we used descriptive statistics, including means and standard deviations (SD) and proportions for continuous and categorical variables, respectively. To be consistent with the standard age-based reporting of adolescent anthropometric measures, demographic and anthropometric outcomes were further disaggregated by age group (late adolescent girls: 15–18.9 y; young women: 19–23 y), and a chi-squared test or t-test was used to determine whether there was a difference between proportions and continuous measures, respectively.

We aimed to generate a hierarchical, multivariable model to examine the associations between the SDoH explanatory variables and BMI categories (base: normal BMI [18.5–24.9 kg/m^2]) and stunting (base: height >145 cm), respectively, using our conceptual framework as a guide. The categories for overweight and obese were combined for the BMI variable, given the low prevalence of obesity observed. Because we found that there was a difference in the prevalence of both BMI categories and stunting between late adolescent girls and young women, and to be consistent with standard age-based reporting of anthropometric measures, a separate model was generated for each age group.

The multi-level model building approach described by Victora et al[33] was followed to generate an adapted hierarchical model with 4-levels. Guided by our conceptual model,[25] we hypothesized that there were four levels of SDoH explanatory variables within the hierarchy and mapped corresponding variables: (1) socioeconomic status (distal-most level): education, occupation,

For peer review only

religion, and wealth quintile; (2) household and personal factors: marital status, parity, and household food security; (3) health and well-being: perception of own health, experience of depression-, anxiety-, and stress-like feelings; and (4) actions and practices (proximal-most level): self-efficacy, participation in decision making, skipping breakfast, and eating dinner with family. For all analyses, the reference for each SDoH explanatory variable was set as the category hypothesized to present the greatest nutritional vulnerability.[25] To investigate the association between the SDoH explanatory variables and each primary outcome measure, crude analyses were first conducted using logistic regression. Variables were considered potentially relevant for inclusion in the multivariable hierarchical model if the unadjusted P was <0.20.[34] Starting with the distal-most level, relevant variables were entered into the model. At each level, a backward-stepwise model building approach was used. Variables were serially removed to observe any change in the β-coefficients of those variables maintained in the model and find a model that best explained the variability in the data. When the P of an explanatory variable was <0.05, it was retained in final model. If the P was ≥0.05 and/or the standard error was high, exclusion of the variable was explored. If the β-coefficients of other variables in the model changed by >10%, the excluded variable was returned, otherwise the variable was dropped. Multicollinearity between variables was assessed using the variance inflation factor (VIF), although we did not find that multicollinearity affected the selected variables using the criteria VIF≥5. The effect estimates in the final model for retained variables correspond to the point of addition to the model. Effect estimates are presented as relative risk ratios (RR) for the variable for BMI categories (three possible outcomes), and odds ratios (OR) for stunting (two possible outcomes). Given the cluster-randomized design of the MaPPS Trial, all models employed cluster robust-standard errors to account for possible correlation between clusters.

For peer review only

RESULTS

Enrolment and exclusion

Of the 32,141 adolescent and young women 15–23 y identified from the household listing exercise, 28,195 (87.7 %) were located, and 25,447 (79.2%) consented to participate in the trial and completed the enrolment questionnaire. Among those enrolled, 14,771 (58.0%) were 15–18.9 y and 10,676 (42.0 %) were 19–23 y, representing 17,044 households.

Participant demographic and reproductive characteristics

At the time of enrolment, participants' mean age (± SD) was 18.8 ± 2.3 y (**Table 1**). No formal education was reported by nearly half of participants, and those with schooling had completed 7.7 ± 3.3 y on average. Currently being a student was reported by 13.8% of participants, of which only 61.1% reported regular attendance. Employment outside the home was reported by 38.5% of participants. Common occupations were unskilled and skilled manual labour (17.5% and 20.2% of participants, respectively), although the majority worked within the home (47.5%). Nearly all participants had experienced menarche (25,305 [99.4%]), with the mean age of onset at 13.0 ± 0.9 y (range: 10–21 y). 22.3% of participants were married, with the mean age at marriage of 16.9 ± 1.8 y (range: 13–23 y), and 13.7% had been pregnant. On average, those married were 4.8 ± 4.2 y younger than their husbands.

For peer review only

Table 1. Demographic and anthropometric characteristics among MaPPS Trial participants at enrolment, disaggregated by age group[1]

**Characteristic**	**All participants** (n=25,447)	**15–18.9 y** (n=14,771)	**19–23 y** (n=10,676)	***P***
Age (y)	18.8 ± 2.3	17.1 ± 1.2	21.1 ± 1.2	<0.001
Highest level of education completed				
None	11,384 (44.7)	4,572 (42.0)	2,758 (44.5)	<0.001
Primary	6,063 (23.8)	2,721 (25.0)	1,412 (22.8)	
Secondary or higher	8,000 (31.5)	4,822 (32.6)	3,178 (29.8)	
Employed outside the home	9,620 (38.1)	5,395 (36.6)	4,225 (40.1)	<0.001
Muslim	23,045 (90.6)	13,288 (90.0)	9,757 (91.4)	<0.001
Wealth quintile				
Poorest	4,549 (17.9)	2,666 (18.0)	1,883 (17.6)	<0.001
Poor	4,853 (19.1)	2,881 (19.5)	1,972 (18.5)	
Middle	5,139 (20.2)	3,025 (20.5)	2,114 (19.8)	
Rich	5,360 (21.1)	3,145 (21.3)	2,215 (20.7)	
Richest	5,546 (21.8)	3,054 (20.7)	2,492 (23.3)	
Currently married[2]	5,685 (22.3)	1,418 (9.6)	4,267 (40.0)	<0.001
Age when married husband (y)[3]	16.9 ± 1.8	15.9 ± 1.1	17.2 ± 1.8	<0.001
Has been pregnant[4]	3,478 (13.7)	572 (3.9)	2,906 (27.2)	<0.001
Age at first pregnancy[5]	17.5 ± 1.6	16.4 ± 1.1	17.7 ± 1.6	<0.001
Weight (kg)	47.0 ± 9.4	45.6 ± 8.5	48.9 ± 10.2	<0.001
Height (cm)	152.5 ± 5.7	152.0 ± 5.6	152.7 ± 5.8	<0.001
BMI (kg/m^2)	20.2 ± 3.7	19.7 ± 3.3	20.9 ± 4.0	<0.001
BMI categorization				
Underweight (<18.5 kg/m^2)	9,396 (36.9)	6,105 (41.3)	3,291 (30.8)	<0.001
Normal (18.5-24.9 kg/m^2)	13,463 (52.9)	7,597 (51.4)	5,866 (54.9)	
Overweight (25-29.9 kg/m^2)	2,011 (7.9)	873 (5.9)	1,138 (10.7)	
Obese (≥30 kg/m^2)	577 (2.3)	196 (1.3)	381 (3.6)	
Middle upper arm circumference (cm)	23.7 ± 3.2	23.3 ± 2.9	24.4 ± 3.4	<0.001

[1]Values are n (%) or mean ± SD
[2]158 participants reported no longer being married
[3]Asked of ever-married women (n_{all} = 5834 [3 women did not know]; $n_{15\text{-}18.9y}$=1,457; $n_{19\text{-}23y}$=4,377)
[4]Due to cultural sensitivities, question only asked of ever-married women (n_{all}=3,451 [28 women did not know]; $n_{15\text{-}18.9y}$=1,457; $n_{19\text{-}23y}$=4,377)
[5]n_{all}=3,451 (28 women did not know); $n_{15\text{-}18.9y}$=567; $n_{19\text{-}23y}$=2,884

For peer review only

Anthropometric characteristics

Regardless of age group, the majority of participants were of 'normal' body weight (52.9%; **Table 1**), although prevalence of underweight was high (36.9%). More late adolescent girls appeared to be underweight, while more young women were overweight/obese (*P*<0.001). Height was <145 cm among 9.2% of participants (late adolescent girls: 10.2%, young women: 7.9%; *P* <0.001), and 35.7% of those stunted were additionally underweight (late adolescent girls: 39.6%, young women: 28.7%; *P* <0.001) and 7.3% overweight/obese (late adolescent girls: 5.4%, young

women: 10.7%; P=0.006). The TEM for weight and height were 0.22 and 0.18, respectively; %TEM were 0.47% and 0.12%; and the R for each measure was >99%, representing high reliability between the repeat measurements. Among adolescent participants <19 y, HAZ and BAZ were found to be -1.58 ± 0.83 and -0.67 ± 1.13, respectively.

Hierarchical model generation

BMI categories and SDoH risk factors among late adolescent girls

For the hierarchical model looking at BMI categories among late adolescent girls, of the SDoH risk factors identified from the crude analyses, those maintained in the final model included occupation, wealth quintile, marital status, food insecurity, reported perception of health, and self-efficacy (P for model: <0.0001; **Table 2**). While a crude association was observed between BMI categories with education level, religion, decision-making, and skipping breakfast, respectively, this was not maintained in the multivariable model (see online supplemental material for all associations and distribution of responses for possible determinants [Table S1]). Compared to those with a normal BMI, those underweight were more likely to be from a poorer wealth quintile, unmarried, food insecure, report poorer overall health, and have lower self-efficacy. Among those overweight/obese, compared to those with a normal BMI, someone was less likely to be employed as an unskilled manual labourer and unmarried, and more likely to be from a higher wealth quintile, food secure, and report good health.

BMI categories and SDoH risk factors among young women

Among young women, SDoH risk factors maintained in the final hierarchical model for BMI included occupation, religion, wealth quintile, marital status, reported perception of health, and decision-making autonomy (P for model: <0.0001; **Table 3**). While a crude association was

observed between BMI categories with food security and self-efficacy, respectively, this was not maintained in the multivariable model (see online supplemental material for all associations and distribution of responses for possible determinants [Table S2]). Compared to those with a normal BMI, those who were underweight were more likely to be from a poorer wealth quintile, non-Muslim, unmarried, and report poorer overall health and participate in less decision-making. Among those overweight/obese, compared to those with a normal BMI, someone was less likely to be employed as an unskilled manual labourer, but more likely to be from a higher wealth quintile and married.

Stunting among late adolescent girls and young women

The SDoH risk factors maintained in hierarchical models for stunting differed between late adolescent girls and young women (**Table 4**). Among late adolescent girls, the final model included variables from three levels of the hierarchy (level 1: education, wealth quintile; level 2: food security; level 4: self-efficacy, decision-making; P for model: <0.0001), while for young women, variables from only the two distal-most levels were maintained (level 1: education, wealth quintile; level 2: parity, food security; P for model: <0.0001). The crude association observed between stunting and occupation, religion, and skipping breakfast, respectively, was not maintained in either multivariable model (see online supplemental material [Table S3]). Compared to those non-stunted, the odds were lower that stunted adolescents had any education or came from a wealthier or food secure household; they experienced lower self-efficacy and made fewer autonomous decisions. Similarly, stunted young women were found to have a lower odd of education and being from a food secure household, as well as a higher odd of participating in unskilled manual labour; once the other variables were considered in the model, they were more likely not to have been pregnant compared to those non-stunted.

For peer review only - http://bmjopen.bmj.com/site/about/guidelines.xhtml

Table 2. Social determinants of health maintained in the hierarchical model of BMI categories (underweight [<18.5 kg/m^2], normal weight [18.5–24.9 kg/m^2; base], and overweight and obese [≥25 kg/m^2]) among late adolescent girls (15–18.9 y) enrolled in the MaPPS Trial at enrolment (n=14,771)[1]

**Possible determinants**	**BMI categories**							
	**Crude**				**Multivariable adjusted**			
	Underweight	*P*	Overweight/ obese	*P*	Underweight	*P*	Overweight/ obese	*P*
Level 1: Structural factors – socioeconomic status								
Occupation								
Unskilled manual labour	(reference)	-	(reference)	-	(reference)	-	(reference)	-
Skilled manual labour	0.85 (0.76 to 0.95)	0.004	2.44 (1.83 to 3.25)	<0.001	0.92 (0.79 to 1.07)	0.26	1.83 (1.42 to 2.36)	<0.001
Within the home	0.83 (0.75 to 0.91)	<0.001	3.28 (2.54 to 4.25)	<0.001	0.94 (0.83 to 1.07)	0.34	2.03 (1.61 to 2.58)	<0.001
Other[2]	0.90 (0.81 to 1.00)	0.06	3.26 (2.48 to 4.30)	<0.001	1.14 (0.94 to 1.37)	0.18	1.44 (1.09 to 1.92)	0.01
Wealth quintile								
Poorest	(reference)	-	(reference)	-	(reference)	-	(reference)	-
Poor	0.84 (0.76 to 0.94)	0.002	1.47 (1.07 to 2.01)	0.001	0.84 (0.74 to 0.95)	0.01	1.34 (1.07 to 1.67)	0.01
Middle	0.79 (0.71 to 0.88)	<0.001	2.36 (1.77 to 3.15)	<0.001	0.78 (0.68 to 0.89)	<0.001	2.07 (1.63 to 2.63)	<0.001
Rich	0.71 (0.64 to 0.79)	<0.001	3.42 (2.59 to 4.51)	<0.001	0.69 (0.58 to 0.82)	<0.001	2.94 (2.32 to 3.74)	<0.001
Richest	0.69 (0.62 to 0.77)	<0.001	5.18 (3.96 to 6.79)	<0.001	0.65 (0.56 to 0.74)	<0.001	4.65 (3.49 to 6.20)	<0.001
Level 2: Intermediary factors - household and personal characteristics								
Marital status								
Married	(reference)		(reference)		(reference)	-	(reference)	-
Unmarried	1.38 (1.23 to 1.56)	<0.001	0.80 (0.66 to 0.97)	<0.03	1.42 (1.28 to 1.59)	<0.001	0.71 (0.59 to 0.85)	<0.001
Household food security status								
Food insecure	(reference)		(reference)		(reference)	-	(reference)	-
Food secure	0.83 (0.77 to 0.90)	<0.001	1.92 (1.61 to 2.28)	<0.001	0.91 (0.83 to 0.99)	0.04	1.24 (1.03 to 1.51)	0.03
Level 3: Intermediary factors - health and well-being characteristics								
Perception of own health								
Poor or fair	(reference)		(reference)		(reference)	-	(reference)	-
Good	0.82 (0.74 to 0.92)	0.001	1.23 (0.97 to 1.57)	0.09	0.83 (0.70 to 0.97)	0.03	1.23 (0.89 to 1.68)	0.21
Excellent	0.71 (0.63 to 0.80)	<0.001	1.40 (1.08 to 1.81)	0.01	0.74 (0.62 to 0.88)	0.001	1.22 (0.93 to 1.60)	0.16
Level 4: Intermediary factors - actions and practices-related characteristics								
Self-efficacy								
Low	(reference)	-	(reference)	-	(reference)	-	(reference)	-
Moderate	0.85 (0.79 to 0.92)	<0.001	1.27 (1.10 to 1.46)	0.001	0.91 (0.85 to 0.97)	<0.001	1.09 (0.93 to 1.27)	0.29
High	0.75 (0.67 to 0.83)	<0.001	1.12 (0.91 to 1.37)	0.29	0.82 (0.72 to 0.92)	<0.001	0.91 (0.73 to 1.14)	0.41

[1]Values are n (%) or RR (95% CI)
[2]Other category included those who identified themselves as students or professionals

Table 3. Social determinants of health maintained in the hierarchical model of BMI categories (underweight [<18.5 kg/m^2], normal weight [18.5–24.9 kg/m^2; base], and overweight and obese [≥25 kg/m^2]) among young women (19–23 y) enrolled in the MaPPS Trial at enrolment (n=10,676)

**Possible determinants**	**BMI categories**							
	**Crude**				**Multivariable adjusted**			
	Underweight	*P*	Overweight/ obese	*P*	Underweight	*P*	Overweight/ obese	*P*
Level 1: Structural factors – socioeconomic status								
Occupation								
Unskilled manual labour	(reference)	-	(reference)	-	(reference)	-	(reference)	-
Skilled manual labour	0.81 (0.71 to 0.93)	0.002	2.77 (2.17 to 3.55)	<0.001	0.95 (0.83 to 1.09)	0.48	1.91 (1.48 to 2.48)	<0.001
Within the home	0.75 (0.67 to 0.84)	<0.001	3.52 (2.81 to 4.42)	<0.001	0.93 (0.83 to 1.05)	0.25	2.07 (1.61 to 2.67)	<0.001
Other[2]	0.76 (0.62 to 0.93)	0.007	3.80 (2.83 to 5.11)	<0.001	1.11 (0.83 to 1.49)	0.47	1.64 (1.19 to 2.26)	0.002
Religion								
Non-Muslim	(reference)	-	(reference)	-	(reference)	-	(reference)	-
Muslim	0.64 (0.56 to 0.74)	<0.001	1.74 (1.35 to 2.25)	<0.001	0.76 (0.61 to 0.93)	0.01	0.98 (0.71 to 1.36)	0.92
Wealth quintile								
Poorest	(reference)	-	(reference)	-	(reference)	-	(reference)	-
Poor	0.81 (0.71 to 0.92)	0.002	1.98 (1.48 to 2.64)	<0.001	0.87 (0.74 to 1.02)	0.09	1.81 (1.36 to 2.43)	<0.001
Middle	0.73 (0.64 to 0.83)	<0.001	3.31 (2.52 to 4.34)	<0.001	0.79 (0.70 to 0.89)	<0.001	2.84 (2.02 to 4.00)	<0.001
Rich	0.62 (0.54 to 0.71)	<0.001	4.68 (3.60 to 6.09)	<0.001	0.68 (0.59 to 0.78)	<0.001	3.88 (2.85 to 5.28)	<0.001
Richest	0.55 (0.48 to 0.63)	<0.001	6.36 (4.92 to 8.22)	<0.001	0.59 (0.48 to 0.72)	<0.001	5.24 (3.93 to 6.98)	<0.001
Level 2: Intermediary factors - household and personal characteristics								
Marital status								
Married	(reference)	-	(reference)	-	(reference)	-	(reference)	-
Unmarried	1.14 (1.04 to 1.24)	0.004	0.90 (0.81 to 1.01)	0.08	1.25 (1.11 to 1.42)	<0.001	0.77 (0.69 to 0.85)	<0.001
Perception of own health								
Poor or fair	(reference)	-	(reference)	-	(reference)	-	(reference)	-
Good	0.78 (0.68 to 0.89)	<0.001	1.18 (0.97 to 1.44)	0.09	0.78 (0.69 to 0.88)	<0.001	1.16 (0.94 to 1.44)	0.18
Excellent	0.75 (0.64 to 0.87)	<0.001	1.35 (1.09 to 1.67)	0.006	0.78 (0.69 to 0.89)	<0.001	1.16 (0.89 to 1.54)	0.27
Decision-making autonomy								
All decisions made by family	(reference)	-	(reference)	-	(reference)	-	(reference)	-
Most decisions made by family	0.91 (0.83 to 1.01)	0.08	1.08 (0.95 to 1.24)	0.23	0.92 (0.85 to 0.99)	0.03	1.08 (0.93 to 1.27)	0.31
Decisions made jointly with family or autonomously	0.85 (0.76 to 0.94)	0.003	1.19 (1.04 to 1.37)	0.01	0.85 (0.77 to 0.95)	0.003	1.24 (1.06 to 1.45)	0.01

[1]Values are n (%) or RR (95% CI)
[2]Other category included those who identified themselves as students or professionals

For peer review only

Table 4. Comparison of social determinants of health in hierarchical model of stunting (height <145 cm) among late adolescent girls (n=14,771) and adult women (n=10,676) enrolled in the MaPPS Trial at enrolment

**Possible determinants**	**Stunting among late adolescent girls**				**Stunting among young women**			
	Crude		Multivariable adjusted		Crude		Multivariable adjusted	
	OR (95% CI)	*P*	OR (95% CI)	*P*	OR (95% CI)	*P*	OR (95% CI)	*P*
Level 1: Structural factors – socioeconomic status								
Highest level of education								
None	(reference)	-	(reference)	-	(reference)	-	(reference)	-
Primary	0.65 (0.57 to 0.74)	<0.001	0.76 (0.62 to 0.94)	0.01	0.62 (0.51 to 0.74)	<0.001	0.76 (0.63 to 0.93)	0.006
Secondary or higher	0.33 (0.29 to 0.38)	<0.001	0.48 (0.39 to 0.59)	<0.001	0.51 (0.43 to 0.61)	<0.001	0.79 (0.66 to 0.95)	0.01
Wealth quintile								
Poorest	(reference)	-	(reference)	-	(reference)	-	(reference)	-
Poor	0.83 (0.72 to 0.97)	0.02	0.91 (0.77 to 1.08)	0.29	0.88 (0.72 to 1.08)	0.22	0.92 (0.73 to 1.17)	0.52
Middle	0.67 (0.57 to 0.78)	<0.001	0.79 (0.65 to 0.96)	0.02	0.65 (0.52 to 0.80)	<0.001	0.70 (0.59 to 0.84)	<0.001
Rich	0.46 (0.39 to 0.55)	<0.001	0.63 (0.49 to 0.81)	<0.001	0.48 (0.38 to 0.60)	<0.001	0.54 (0.40 to 0.74)	<0.001
Richest	0.26 (0.21 to 0.32)	<0.001	0.42 (0.35 to 0.51)	<0.001	0.34 (0.27 to 0.43)	<0.001	0.41 (0.29 to 0.57)	<0.001
Level 2: Intermediary factors - household and personal characteristics								
Ever been pregnant								
Yes	(reference)	-	-	-	(reference)	-	(reference)	-
No	0.97 (0.74 to 1.27)	0.81	-	-	1.13 (0.96 to 1.32)	0.15	1.27 (1.10 to 1.47)	0.001
Household food security status								
Food insecure	(reference)	-	(reference)	-	(reference)	-	(reference)	-
Food secure	0.58 (0.51 to 0.64)	<0.001	0.77 (0.66 to 0.90)	0.001	0.57 (0.49 to 0.66)	<0.001	0.73 (0.59 to 0.90)	0.003
Level 4: Intermediary factors - actions and practices-related characteristics								
Self-efficacy								
Low	(reference)	-	(reference)		(reference)	-	-	-
Moderate	0.62 (0.56 to 0.70)	<0.001	0.74 (0.64 to 0.84)	<0.001	0.83 (0.71 to 0.98)	0.03	-	-
High	0.59 (0.50 to 0.71)	<0.001	0.74 (0.62 to 0.87)	<0.001	0.78 (0.63 to 0.97)	0.03	-	-
Decision-making autonomy								
All decisions made by family	(reference)	-	(reference)	-	(reference)	-	-	-
Most decisions made by family	0.83 (0.74 to 0.94)	0.003	0.88 (0.76 to 1.01)	0.08	0.99 (0.84 to 1.16)	0.90	-	-
Decisions made jointly with family or autonomously	0.74 (0.62 to 0.89)	0.001	0.76 (0.62 to 0.92)	0.01	0.85 (0.71 to 1.02)	0.09	-	-

For peer review only - http://bmjopen.bmj.com/site/about/guidelines.xhtml

DISCUSSION

Using enrolment data from a large trial conducted in rural Pakistan, we found high levels of undernutrition among late adolescent and young women 15–23 y using anthropometric indicators. Our study offers the most comprehensive analysis of the determinants of malnutrition among late adolescent girls and young women in rural Pakistan. Across the multivariable models for acute and chronic measures of undernutrition, those who were impoverished were at increased risk regardless of age group. This suggests that structural factors play an important, underlying role in female nutrition in this setting. That an emerging burden of overweight/obesity was also observed in this rural population indicates that interventions are needed to address both ends of the malnutrition spectrum. To the best of our knowledge, this is the largest survey of a cohort that includes both unmarried and married Pakistani late adolescent girls and young women.

Pakistan is known to have a high burden of malnutrition, with a long term impact on human capital and costing an estimated 7.6 billion USD/year.[35] We found the mean BMI among study participants was lower than the PDHS national average (20.2 kg/m^2 versus 25.7 kg/m^2).[17] This likely reflects that the PDHS only surveyed ever-married WRA (15–49 y). In the recently completed 2018 Pakistan National Nutrition Survey, 22.6%, 19.8%, and 10.2% of WRA in Sindh province were found to be underweight, overweight, and obese, respectively.[36] Notably, both the PDHS and National Nutrition Survey include urban settings, where the risk of overweight and obesity is higher.[37]

For peer review only

Of the limited adolescent nutrition studies available globally, wealth has consistently been found to be a determinant of undernutrition in multiple settings.[38-42] Regardless of whether someone was an adolescent or young women, we found that at least one structural SDoH variable tied to poverty was associated with an increased risk of undernutrition outcomes. Notably, food insecurity was maintained in several models. At least one variable considered a proxy for empowerment was maintained in each adolescent model, with greater empowerment being associated with an improvement in nutritional status. Considered a blend of resources (materials that allow someone to make her goals a reality), agency (the ability to define and act upon someone's goals), and achievement (obtaining a desired goal), empowerment enables someone to make decisions to take charge of her own life and affect her personal circumstances.[43] Empowered women are widely recognized to be better able to promote their own health and well-being; whereas, lacking empowerment is suggested to present a further barriers to achieving optimal nutritional status.[3]

We had hypothesized that married late adolescent and young women might be at greater risk for undernutrition because early marriage has been suggested to increase nutritional vulnerability;[44] however, in the hierarchical models for both adolescent and young women, being married was associated with increased likelihood of being in a higher BMI category compared to those unmarried. This it is likely attributable to an increase in status within the household, as married participants contributed more to food-related decision-making (data not shown). An association between marital status and stunting, a chronic measure of poor nutrition across the lifespan, was not observed.

For peer review only - http://bmjopen.bmj.com/site/about/guidelines.xhtml

There is a recognized need in Pakistan to focus on the health and nutrition of adolescent girls,[45 46] and it is well documented that preconception health and well-being are important to the individual, as well as to her future offspring.[47] To address inequalities due to poverty, the use of social safety nets could function as an important resource. As food insecurity prevalence was notable, provision of cash transfers, for example, could lend to the purchase of more nutritious food, although we are not certain at this time of the exact effect that this might have on an adolescent or young women's actual intake. However, economic status alone does not confer a healthy BMI.[42] We would note that there will need to be a concurrent emphasis on maintaining a healthy BMI, given that the prevalence of overweight/obesity in Pakistan has increased over time in both urban and rural settings, the existing double burden of malnutrition, and ongoing nutrition transition. To address structural and empowerment-related barriers, increased and coordinated efforts between the public health system and the education sector could have potential. The Government of Pakistan has recently released its nutritional strategy and operational plan for targeting adolescent malnutrition, which will be important to implementing legislation on adolescent nutrition.[48]

Limitations of this study include that our catchment area was restricted to the areas of Matiari District covered by the LHW Programme. Furthermore, our findings are specific to the context in rural Pakistan, and findings may not reflect settings with different cultural norms or burdens of undernutrition, as seen in Africa and South America.[49] While BMI is a globally accepted index of nutritional status, the relationship between BMI and body composition can be controversial, as BMI does not account for excess weight due to adiposity versus muscularity, edema, or bone mass.[6] It would have been informative to have had additional measures of body composition

For peer review only

given its influence on health. The SDoH considered within this study were proxy variables and required self-reporting, thus there could be measurement error (e.g. social desirability bias). It is also possible that all SDoH relevant to context were not considered, although the assessed explanatory variables were informed by the literature. We additionally acknowledge that the adolescent period does commence at 10 y, although given the aims of the MaPPS Trial we did not enrol adolescent girls 10–14 y. The larger study focused on adolescent and young women, not adolescent boys or young men; yet, males are also important given their earning potential and contribution to the economy.[46] A further similar evaluation of this group would be informative, and aid understanding gendered cultural norms. Although the assessment of macro-level SDoH (e.g., governance, policies) was beyond the scope of this assessment, the importance of structural SDoH to all models suggests that underlying factors that create divisions within society are precarious. In their cross-sectional assessment of the factors affecting thinness among adolescent girls 10–19 y in low and middle income countries, Candler et al[38] found that macroeconomic factors contribute to the genesis of adolescent thinness, and that reducing adolescent thinness should include addressing national food security and economic development.

For peer review only

CONCLUSIONS

Given the findings of this study, and the concerning status of malnutrition in Pakistan, commitment to improving the nutrition of adolescent and young women in Pakistan is critical and should include those unmarried. Reaching these individuals is important not only because of their reproductive potential, but also given their right to optimal nutrition and well-being.[50] To

target undernutrition, particularly, there is a need for both direct and indirect nutrition and empowerment interventions that are integrated and multi-sectoral,[51] as highlighted in the recent national adolescent nutrition policy recommendations.[48] An ideal program for adolescent and young women should be inclusive and accommodating, reach those who are most vulnerable, and account for cultural- and gender-relevant factors.[52]

For peer review only

Acknowledgements: The authors wish to acknowledge the participants in the MaPPS Trial and the field research team. We are grateful for the ongoing data-related assistance and support provided by Rasool Bux, Arjumand Rizvi, and the members of the Data Management Unit at Aga Khan University.

Contributions: JBB and ZAB designed research; YW and SBS conducted research; IA and AH performed data management; JBB analyzed data; JBB wrote first manuscript draft; ZAB, YW, SBS, IA, and AH provided manuscript feedback; ZAB has primary responsibility for final content. All authors reviewed this manuscript and approved the final version.

Funding: The MaPPS Trial was supported by the Bill and Melinda Gates Foundation (grant number: OPP1148892) and the World Food Programme (grant number: HQ15NF493 - CTR). JBB received research scholarships from the Vanier Canada Graduate Scholarship; Hospital for Sick Children Foundation Student Scholarship Program; and the Province of Ontario.

Competing interests: None declared.

Ethical approval: Ethical review for this study was obtained from the Aga Khan University Ethics Review Committee (Protocol #4324-Ped-ERC-16) and the Hospital for Sick Children Research Ethics Board (Protocol #1000054682).

Data availability statement: The anonymized, individual-level data from this cross-sectional assessment, situated within a clinical trial, will be made available upon reasonable request from the corresponding author.

REFERENCES

1. World Health Organization. Guideline: implementing effective actions for improving adolescent nutrition. Geneva: World Health Organization, 2018.

2. Sawyer SM, Afifi RA, Bearinger LH, et al. Adolescence: a foundation for future health. *Lancet* 2012;379(9826):1630-40.

3. Christian P, Smith ER. Adolescent Undernutrition: Global Burden, Physiology, and Nutritional Risks. *Ann Nutr Metab* 2018;72(4):316-28.

4. Norris SA, Frongillo EA, Black MM, et al. Nutrition in adolescent growth and development. *Lancet* 2022;399(10320):172-84. doi: 10.1016/s0140-6736(21)01590-7 [published Online First: 2021/12/03]

5. Aguayo VM, Paintal K. Nutrition in adolescent girls in South Asia. *BMJ* 2017;357:j1309. doi: 10.1136/bmj.j1309 [published Online First: 2017/04/13]

6. Gibson R. Principles of Nutritional Assessment. New York: Oxford University Press 2005.

For peer review only

7. Black RE, Victora CG, Walker SP, et al. Maternal and child undernutrition and overweight in low-income and middle-income countries. *Lancet* 2013;382(9890):427-51.

8. Twig G, Yaniv G, Levine H, et al. Body mass index in 2.3 million adolescents and cardiovascular death in adulthood. *N Engl J Med* 2016;374:2430–40.

9. Saydah S, Bullard KM, Imperatore G, et al. Cardiometabolic risk factors among US adolescents and young adults and risk of early mortality. *Pediatrics* 2013;131:e679-86. .

10. Viner RM, Ozer EM, Denny S, et al. Adolescence and the social determinants of health. *Lancet* 2012;379(9826):1641-52.

11. Commission on Social Determinants of Health. Closing the gap in a generation: health equity through action on the social determinants of health. Geneva: World Health Organization, 2008.

12. Fatusi AO, Bello B. Social Determinants of Health in Adolescent and Young Women's Health and Nutrition: Current Evidence. In: Bhutta ZA, Makarides M, Prentice AM, eds. Health and Nutrition in Adolescents and Young Women: Preparing for the Next Generation. Basel: Nestle Nutr Inst Workshop Ser 2015:61-69.

13. United Nations. Transforming Our World: The 2030 Agenda for Sustainable Development. New York: United Nations 2015.

14. Christian P, Smith E. Adolescent Undernutrition: Global Burden, Physiology, and Nutritional Risks. *Ann Nutr Metab* 2018;72(4):316-28.

15. Bhutta Z, Lassi Z, Bergeron G, et al. Delivering an action agenda for nutrition interventions addressing adolescent girls and young women: priorities for implementation and research. *Ann N Y Acad Sci* 2017;1393(1):61-71.

16. Ruel MT, Alderman H. Nutrition-sensitive interventions and programmes: how can they help to accelerate progress in improving maternal and child nutrition? *Lancet* 2013;382(9891):536-51. doi: 10.1016/s0140-6736(13)60843-0 [published Online First: 2013/06/12]

17. National Institute of Population Studies (NIPS) [Pakistan] and ICF. Pakistan Demographic and Health Survey 2017-18. Islamabad, Pakistan, and Rockville, Maryland, USA: NIPS and ICF, 2019.

For peer review only

18. Baxter JB, Wasan Y, Soofi SB, et al. Effect of life skills building education and micronutrient supplements provided from preconception versus the standard of care on low birth weight births among adolescent and young Pakistani women (15-24 years): a prospective, population-based cluster-randomized trial. *Reprod Health* 2018;15(1):104.

19. Centers for Disease Control and Prevention. National Health and Nutrition Examination Survey: anthropometry procedures manual. Hyattsville, MD: National Center for Health Statistics, Centers for Disease Control and Prevention, 2009.

20. Ulijaszek SJ, Kerr, D.A.,. Anthropometric measurement error and the assessment of nutritional status. *Br J Nutr* 1999;82:165–77.

21. World Health Organization. 2018 Global Reference List of 100 Core Health Indicators (plus health-related SDGs). Geneva: World Health Organization, 2018.

22. Baxter JB, Kortenaar JL, Wasan Y, et al. Age-Based Anthropometric Cutoffs Provide Inconsistent Estimates of Undernutrition: Findings from a Cross-Sectional Assessment of Late-Adolescent and Young Women in Rural Pakistan. *Curr Dev Nutr* 2021;5(11):nzab130. doi: 10.1093/cdn/nzab130 [published Online First: 2021/12/14]

23. Tumilowicz A, Beal T, Neufeld LM, et al. Perspective: Challenges in Use of Adolescent Anthropometry for Understanding the Burden of Malnutrition. *Adv Nutr* 2019;10(4):563-75.

24. de Onis M, Onyango AW, Borghi E, et al. Development of a WHO growth reference for school-aged children and adolescents. *Bull World Health Organ* 2007;85:660–67.

25. Baxter JB, Wasan Y, Hussain A, et al. Characterizing Micronutrient Status and Risk Factors among Late Adolescent and Young Women in Rural Pakistan: A Cross-Sectional Assessment of the MaPPS Trial. *Nutrients* 2021;13:1237.

26. UNICEF. The State of the World's Children [Internet]. New York: Oxford University Press 1998.

27. Solar O, Irwin A. A conceptual framework for action on the social determinants of health. Social Determinants of Health Discussion Paper 2 (Policy and Practice). Geneva: World Health Organization, 2010.

For peer review only

28. Lovibond SH, Lovibond PF. Manual for the Depression Anxiety Stress Scales. Sydney: Psychology Foundation, 1995.

29. Coates J, Swindale A, Bilinsky P. Household food insecurity access scale (HFIAS) for measurement of household food access: Indicator guide (v. 3). Washington: Food and Nutrition Technical Assistance Project, Academy for Educational Development, 2007.

30. Currie C, Inchley J, Molcho M, et al. Health Behaviour in School-aged Children (HBSC) Study Protocol: Background, Methodology, and Mandatory items for the 2013/14 Survey. St. Andrews: CAHRU, 2014.

31. Schwarzer R, Jerusalem M. Generalized self-efficacy scale. In: Weinman J WS, Johnston M, ed. Measures in health psychology: a user's portfolio Causal and control beliefs. Windsor: NFER-NELSON 1995:35-37.

32. Ewerling F, Lynch JW, Victora CG, et al. The SWPER index for women's empowerment in Africa: development and validation of an index based on survey data. *Lancet Glob Health* 2017;5(9):e916-e23.

33. Victora CG, Huttly SR, Fuchs SC, et al. The role of conceptual frameworks in epidemiological analysis: a hierarchical approach. *Int J Epidemiol* 1997;26:224-27.

34. Hosmer DW, Lemeshow S. Applied Logistic Regression. 2nd ed. New York: John Wiley 2000.

35. World Food Programme. The Economic Consequences of Undernutrition in Pakistan: An Assessment of Losses. Islamabad: World Food Programme Pakistan, 2017.

36. UNICEF Pakistan. National Nutrition Survey 2018: Key Findings Report. Islamabad: UNICEF Pakistan 2018.

37. Janjua NZ, Mahmood B, Bhatti JA, et al. Association of household and community socioeconomic position and urbanicity with underweight and overweight among women in Pakistan. *PLoS One* 2015;10(4):e0122314.

For peer review only

38. Candler T, Costa S, Heys M, et al. Prevalence of Thinness in Adolescent Girls in Low- and Middle-Income Countries and Associations With Wealth, Food Security, and Inequality. . *J Adolesc Health* 2017;60(4):447-54.

39. Harding KL, Aguayo VM, Webb P. Trends and Correlates of Overweight among Pre-School Age Children, Adolescent Girls, and Adult Women in South Asia: An Analysis of Data from Twelve National Surveys in Six Countries over Twenty Years. *Nutrients* 2019;11(8):1899.

40. Leroy JL, Ruel M, Sununtnasuk C, et al. Understanding the determinants of adolescent nutrition in Bangladesh. *Ann N Y Acad Sci* 2018

41. Radhika MS, Swetha B, Kumar NB, et al. Dietary and nondietary determinants of nutritional status among adolescent girls and adult women in India. *Ann N Y Acad Sci* 2018;1416(1):5-17.

42. Young MF, Nguyen P, Tran LM, et al. A Double Edged Sword? Improvements in Economic Conditions over a Decade in India Led to Declines in Undernutrition as Well as Increases in Overweight among Adolescents and Women. *J Nutr* 2020;150(2):364-72. doi: 10.1093/jn/nxz251 [published Online First: 2019/10/17]

43. Kabeer N. Resources, agency, achievements: reflections on the measurement of women's empowerment. *Dev Change* 1999;30:435-64.

44. Marphatia AA, Ambale GS, Reid AM. Women's Marriage Age Matters for Public Health: A Review of the Broader Health and Social Implications in South Asia. *Front Public Health* 2017;5:269. doi: 10.3389/fpubh.2017.00269 [published Online First: 2017/11/03]

45. Government of Pakistan. National Vision 2016-2025 for Coordinated Priority Actions to Address Challenges of Reproductive, Maternal, Newborn, Child, Adolescent Health and Nutrition. Islamabad: Government of Pakistan, 2015.

46. Government of Pakistan, World Health Organization. Adolescent Nutrition and Supplementation Guidelines for Pakistan. Islamabad: Government of Pakistan, 2019.

47. Dean SV, Lassi ZS, Imam AM, et al. Preconception care: nutritional risks and interventions. *Reprod Health* 2014;11(Suppl 3):S3.

For peer review only

48. Government of Pakistan. Pakistan Adolescent Nutrition Strategy and Operations Plan. Karachi: Government of Pakistan 2020.

49. Akseer N, Al-Gashm S, Mehta S, et al. Global and regional trends in the nutritional status of young people: a critical and neglected age group. *Ann N Y Acad Sci* 2017;1393(1):3-20.

50. Fox EL, Davis C, Downs SM, et al. Who is the Woman in Women's Nutrition? A Narrative Review of Evidence and Actions to Support Women's Nutrition throughout Life. *Current Developments in Nutrition* 2018;3(1) doi: 10.1093/cdn/nzy076

51. Keats E, Das JK, Salam RA, et al. Effective interventions to address maternal and child malnutrition: an update of the evidence. *Lancet Child Adolesc Health* 2021 doi: 10.1016/s2352-4642(20)30274-1 [published Online First: 2021/03/11]

52. Hargreaves D, Mates E, Menon P, et al. Strategies and interventions for healthy adolescent growth, nutrition, and development. *Lancet* 2022;399(10320):198-210. doi: 10.1016/s0140-6736(21)01593-2 [published Online First: 2021/12/03]

For peer review only

Supplementary Material

Table S1. Comparison of social determinants of health with BMI categories (underweight [<18.5 kg/m^2], normal weight [18.5–24.9 kg/m^2; base], and overweight and obese [≥25 kg/m^2]) among late adolescent girls (15–18.9 y) enrolled in the MaPPS Trial at enrolment (n=14,771)[1]

**Possible determinants**	**n (%)**	**BMI categories**							
		**Crude**				**Multivariable adjusted**			
		Underweight	*P*	Overweight/ obese	*P*	Underweight	*P*	Overweight/ obese	*P*
Level 1: Structural factors – socioeconomic status									
Highest level of education									
None	6,297 (42.6)	(reference)	-	(reference)	-	-	-	-	-
Primary	3,652 (24.7)	0.96 (0.89 to 1.05)	0.39	1.46 (1.23 to 1.74)	<0.001	-	-	-	-
Secondary or higher	4,822 (32.6)	0.86 (0.80 to 0.93)	<0.001	2.05 (1.76 to 2.38)	<0.001	-	-	-	-
Occupation									
Unskilled manual labour	2,622 (17.8)	(reference)	-	(reference)	-	(reference)	-	(reference)	-
Skilled manual labour	2,762 (18.7)	0.85 (0.76 to 0.95)	0.004	2.44 (1.83 to 3.25)	<0.001	0.92 (0.79 to 1.07)	0.26	1.83 (1.42 to 2.36)	<0.001
Within the home	6,351 (43.0)	0.83 (0.75 to 0.91)	<0.001	3.28 (2.54 to 4.25)	<0.001	0.94 (0.83 to 1.07)	0.34	2.03 (1.61 to 2.58)	<0.001
Other[2]	3,036 (20.6)	0.90 (0.81 to 1.00)	0.06	3.26 (2.48 to 4.30)	<0.001	1.14 (0.94 to 1.37)	0.18	1.44 (1.09 to 1.92)	0.01
Religion									
Non-Muslim	1,483 (10.0)	(reference)	-	(reference)	-	-	-	-	-
Muslim	13,288 (90.0)	0.79 (0.71 to 0.88)	<0.001	1.96 (1.48 to 2.61)	<0.001	-	-	-	-
Wealth quintile									
Poorest	2,666 (18.0)	(reference)	-	(reference)	-	(reference)	-	(reference)	-
Poor	2,881 (19.5)	0.84 (0.76 to 0.94)	0.002	1.47 (1.07 to 2.01)	0.001	0.84 (0.74 to 0.95)	0.01	1.34 (1.07 to 1.67)	0.01
Middle	3,025 (20.5)	0.79 (0.71 to 0.88)	<0.001	2.36 (1.77 to 3.15)	<0.001	0.78 (0.68 to 0.89)	<0.001	2.07 (1.63 to 2.63)	<0.001
Rich	3,145 (21.3)	0.71 (0.64 to 0.79)	<0.001	3.42 (2.59 to 4.51)	<0.001	0.69 (0.58 to 0.82)	<0.001	2.94 (2.32 to 3.74)	<0.001
Richest	3,054 (20.7)	0.69 (0.62 to 0.77)	<0.001	5.18 (3.96 to 6.79)	<0.001	0.65 (0.56 to 0.74)	<0.001	4.65 (3.49 to 6.20)	<0.001
Level 2: Intermediary factors - household and personal characteristics									
Marital status									
Married	1,418 (9.6)	(reference)		(reference)		(reference)	-	(reference)	-
Unmarried	13,353 (90.4)	1.38 (1.23 to 1.56)	<0.001	0.80 (0.66 to 0.97)	<0.03	1.42 (1.28 to 1.59)	<0.001	0.71 (0.59 to 0.85)	<0.001
Ever been pregnant									
Yes	572 (3.9)	(reference)	-	(reference)	-	-	-	-	-
No	14,199 (96.1)	1.14 (0.96 to 1.36)	0.14	0.80 (0.59 to 1.08)	0.14	-	-	-	-
Household food security status									
Food insecure	3,861 (26.1)	(reference)		(reference)		(reference)	-	(reference)	-
Food secure	10,910 (73.9)	0.83 (0.77 to 0.90)	<0.001	1.92 (1.61 to 2.28)	<0.001	0.91 (0.83 to 0.99)	0.04	1.24 (1.03 to 1.51)	0.03
Level 3: Intermediary factors - health and well-being characteristics									
Perception of own health									
Poor or fair	1,533 (10.4)	(reference)		(reference)		(reference)	-	(reference)	-

For peer review only

Possible determinants	n (%)	BMI categories							
		Crude				Multivariable adjusted			
		Underweight	*P*	Overweight/ obese	*P*	Underweight	*P*	Overweight/ obese	*P*
Good	9,731 (65.9)	0.82 (0.74 to 0.92)	0.001	1.23 (0.97 to 1.57)	0.09	0.83 (0.70 to 0.97)	0.03	1.23 (0.89 to 1.68)	0.21
Excellent	3,507 (23.7)	0.71 (0.63 to 0.80)	<0.001	1.40 (1.08 to 1.81)	0.01	0.74 (0.62 to 0.88)	0.001	1.22 (0.93 to 1.60)	0.16
Experience of depression-like feelings									
Severe or extremely severe	687 (4.7)	(reference)	-	(reference)	-	-	-	-	-
Moderate	1,545 (10.5)	0.93 (0.78 to 1.12)	0.47	1.16 (0.78 to 1.71)	0.46	-	-	-	-
Mild	1,236 (8.4)	0.94 (0.78 to 1.14)	0.53	1.15 (0.76 to 1.72)	0.51	-	-	-	-
None	11,303 (76.5)	0.78 (0.67 to 0.92)	0.002	1.20 (0.85 to 1.68)	0.30	-	-	-	-
Experience of anxiety-like feelings									
Severe or extremely severe	1,893 (12.8)	(reference)	-	(reference)	-	-	-	-	-
Moderate	2,124 (14.4)	1.02 (0.90 to 1.16)	0.78	0.96 (0.74 to 1.25)	0.77	-	-	-	-
Mild	952 (6.4)	0.85 (0.72 to 1.00)	0.05	1.06 (0.77 to 1.44)	0.73	-	-	-	-
None	9,802 (66.4)	0.79 (0.72 to 0.88)	<0.001	1.04 (0.85 to 1.27)	0.72	-	-	-	-
Experience of stress-like feelings									
Severe or extremely severe	491 (3.3)	(reference)	-	(reference)	-	-	-	-	-
Moderate	778 (5.3)	0.94 (0.75 to 1.19)	0.61	0.88 (0.54 to 1.41)	0.59	-	-	-	-
Mild	885 (6.0)	0.91 (0.72 to 1.14)	0.40	1.07 (0.68 to 1.68)	0.78	-	-	-	-
None	12,617 (85.4)	0.80 (0.67 to 0.97)	0.02	1.03 (0.70 to 1.49)	0.89	-	-	-	-
Level 4: Intermediary factors - actions and practices-related characteristics									
Self-efficacy									
Low	5,695 (38.6)	(reference)	-	(reference)	-	(reference)	-	(reference)	-
Moderate	7,161 (48.5)	0.85 (0.79 to 0.92)	<0.001	1.27 (1.10 to 1.46)	0.001	0.91 (0.85 to 0.97)	<0.001	1.09 (0.93 to 1.27)	0.29
High	1,915 (13.0)	0.75 (0.67 to 0.83)	<0.001	1.12 (0.91 to 1.37)	0.29	0.82 (0.72 to 0.92)	<0.001	0.91 (0.73 to 1.14)	0.41
Decision-making autonomy									
All decisions made by family	8,244 (55.8)	(reference)	-	(reference)	-	-	-	-	-
Most decisions made by family	4,702 (31.8)	0.9 (0.84 to 0.97)	0.008	1.18 (1.03 to 1.36)	0.02	-	-	-	-
Decisions made jointly with family or autonomously	1,825 (12.4)	0.84 (0.76 to 0.94)	0.002	1.32 (1.10 to 1.60)	0.004	-	-	-	-
Skipping breakfast									
Skips breakfast	4,479 (30.3)	(reference)	-	(reference)	-	-	-	-	-
Eats breakfast	10,292 (69.7)	0.99 (0.92 to 1.07)	0.81	1.18 (1.02 to 1.36)	0.03	-	-	-	-
Eating dinner with family									
Never	4,021 (27.2)	(reference)	-	(reference)	-	-	-	-	-
Sometimes	1,869 (12.7)	0.99 (0.89 to 1.12)	0.93	1.12 (0.90 to 1.40)	0.30	-	-	-	-
Everyday	8,881 (60.1)	0.95 (0.88 to 1.03)	0.23	1.13 (0.97 to 1.32)	0.11	-	-	-	-

[1]Values are n (%) or RR (95% CI)
[2]Other category included those who identified themselves as students (2,988[20.2%]) or professionals (48[0.3%])

For peer review only

Table S2. Comparison of social determinants of health with BMI categories (underweight [<18.5 kg/m^2], normal weight [18.5–24.9 kg/m^2; base], and overweight and obese [≥25 kg/m^2]) among young women (19–23 y) enrolled in the MaPPS Trial at enrolment (n=10,676)

**Possible determinants**	**n (%)**	**BMI categories**							
		**Crude**				**Multivariable adjusted**			
		Underweight	*P*	Overweight/ obese	*P*	Underweight	*P*	Overweight/ obese	*P*
Level 1: Structural factors – socioeconomic status									
Highest level of education									
None	5,087 (47.6)	(reference)	-	(reference)	-	-	-	-	-
Primary	2,411 (22.6)	0.80 (0.71 to 0.89)	<0.001	1.71 (1.47 to 1.98)	<0.001	-	-	-	-
Secondary or higher	3,178 (29.8)	0.74 (0.66 to 0.82)	<0.001	2.21 (1.94 to 2.52)	<0.001	-	-	-	-
Occupation									
Unskilled manual labour	1,837 (17.2)	(reference)	-	(reference)	-	(reference)	-	(reference)	-
Skilled manual labour	2,372 (22.2)	0.81 (0.71 to 0.93)	0.002	2.77 (2.17 to 3.55)	<0.001	0.95 (0.83 to 1.09)	0.48	1.91 (1.48 to 2.48)	<0.001
Within the home	5,779 (54.1)	0.75 (0.67 to 0.84)	<0.001	3.52 (2.81 to 4.42)	<0.001	0.93 (0.83 to 1.05)	0.25	2.07 (1.61 to 2.67)	<0.001
Other[2]	688 (6.4)	0.76 (0.62 to 0.93)	0.007	3.80 (2.83 to 5.11)	<0.001	1.11 (0.83 to 1.49)	0.47	1.64 (1.19 to 2.26)	0.002
Religion									
Non-Muslim	919 (8.6)	(reference)	-	(reference)	-	(reference)	-	(reference)	-
Muslim	9,757 (91.4)	0.64 (0.56 to 0.74)	<0.001	1.74 (1.35 to 2.25)	<0.001	0.76 (0.61 to 0.93)	0.01	0.98 (0.71 to 1.36)	0.92
Wealth quintile									
Poorest	1,883 (17.6)	(reference)	-	(reference)	-	(reference)	-	(reference)	-
Poor	1,972 (18.5)	0.81 (0.71 to 0.92)	0.002	1.98 (1.48 to 2.64)	<0.001	0.87 (0.74 to 1.02)	0.09	1.81 (1.36 to 2.43)	<0.001
Middle	2,114 (19.8)	0.73 (0.64 to 0.83)	<0.001	3.31 (2.52 to 4.34)	<0.001	0.79 (0.70 to 0.89)	<0.001	2.84 (2.02 to 4.00)	<0.001
Rich	2,215 (20.7)	0.62 (0.54 to 0.71)	<0.001	4.68 (3.60 to 6.09)	<0.001	0.68 (0.59 to 0.78)	<0.001	3.88 (2.85 to 5.28)	<0.001
Richest	2,492 (23.3)	0.55 (0.48 to 0.63)	<0.001	6.36 (4.92 to 8.22)	<0.001	0.59 (0.48 to 0.72)	<0.001	5.24 (3.93 to 6.98)	<0.001
Level 2: Intermediary factors - household and personal characteristics									
Marital status									
Married	4,267 (40.0)	(reference)	-	(reference)	-	(reference)	-	(reference)	-
Unmarried	6,409 (60.0)	1.14 (1.04 to 1.24)	0.004	0.90 (0.81 to 1.01)	0.08	1.25 (1.11 to 1.42)	<0.001	0.77 (0.69 to 0.85)	<0.001
Ever been pregnant									
Yes	2,906 (27.2)	(reference)	-	(reference)	-	-	-	-	-
No	7,770 (72.8)	1.03 (0.93 to 1.13)	0.58	0.93 (0.82 to 1.06)	0.28	-	-	-	-
Household food security status									
Food insecure	2,494 (23.4)	(reference)	-	(reference)	-	-	-	-	-
Food secure	8,182 (76.6)	0.79 (0.71 to 0.87)	<0.001	1.77 (1.52 to 2.07)	<0.001	-	-	-	-
Level 3: Intermediary factors - health and well-being characteristics									
Perception of own health									
Poor or fair	1,275 (11.9)	(reference)	-	(reference)	-	(reference)	-	(reference)	-
Good	6,828 (64.0)	0.78 (0.68 to 0.89)	<0.001	1.18 (0.97 to 1.44)	0.09	0.78 (0.69 to 0.88)	<0.001	1.16 (0.94 to 1.44)	0.18
Excellent	2,573 (24.1)	0.75 (0.64 to 0.87)	<0.001	1.35 (1.09 to 1.67)	0.006	0.78 (0.69 to 0.89)	<0.001	1.16 (0.89 to 1.54)	0.27
Experience of depression-like feelings									
Severe or extremely severe	537 (5.0)	(reference)	-	(reference)	-	-	-	-	-

For peer review only

Possible determinants	n (%)	BMI categories							
		Crude				Multivariable adjusted			
		Underweight	*P*	Overweight/ obese	*P*	Underweight	*P*	Overweight/ obese	*P*
Moderate	1,108 (10.4)	1.02 (0.82 to 1.28)	0.84	1.37 (0.97 to 1.93)	0.08	-	-	-	-
Mild	922 (8.6)	1.01 (0.8 to 1.28)	0.92	1.57 (1.11 to 2.23)	0.01	-	-	-	-
None	8,109 (76.0)	0.87 (0.72 to 1.06)	0.16	1.49 (1.11 to 2.01)	0.009	-	-	-	-
Experience of anxiety-like feelings									
Severe or extremely severe	1,421 (13.3)	(reference)	-	(reference)	-	-	-	-	-
Moderate	1,514 (14.2)	0.94 (0.80 to 1.10)	0.44	0.91 (0.73 to 1.15)	0.44	-	-	-	-
Mild	732 (6.9)	0.71 (0.58 to 0.87)	0.001	0.72 (0.54 to 0.97)	0.03	-	-	-	-
None	7,009 (65.7)	0.81 (0.72 to 0.92)	0.001	1.17 (0.98 to 1.40)	0.08	-	-	-	-
Experience of stress-like feelings									
Severe or extremely severe	405 (3.8)	(reference)	-	(reference)	-	-	-	-	-
Moderate	578 (5.4)	1.15 (0.87 to 1.52)	0.33	1.07 (0.72 to 1.59)	0.74	-	-	-	-
Mild	621 (5.8)	0.96 (0.72 to 1.26)	0.76	1.24 (0.85 to 1.80)	0.27	-	-	-	-
None	9,072 (85.0)	0.88 (0.71 to 1.10)	0.27	1.08 (0.79 to 1.47)	0.62	-	-	-	-
Level 4: Intermediary factors - actions and practices-related characteristics									
Self-efficacy									
Low	2,937 (27.5)	(reference)	-	(reference)	-	-	-	-	-
Moderate	5,709 (53.5)	0.92 (0.83 to 1.02)	0.10	1.34 (1.16 to 1.54)	<0.001	-	-	-	-
High	2,030 (19.0)	0.83 (0.73 to 0.95)	0.005	1.40 (1.18 to 1.66)	<0.001	-	-	-	-
Decision-making autonomy									
All decisions made by family	4,365 (40.9)	(reference)	-	(reference)	-	(reference)	-	(reference)	-
Most decisions made by family	3,630 (34.0)	0.91 (0.83 to 1.01)	0.08	1.08 (0.95 to 1.24)	0.23	0.92 (0.85 to 0.99)	0.03	1.08 (0.93 to 1.27)	0.31
Decisions made jointly with family or autonomously	2,681 (25.1)	0.85 (0.76 to 0.94)	0.003	1.19 (1.04 to 1.37)	0.01	0.85 (0.77 to 0.95)	0.003	1.24 (1.06 to 1.45)	0.01
Skipping breakfast									
Skips breakfast	3,130 (29.3)	(reference)	-	(reference)	-	-	-	-	-
Eats breakfast	7,546 (70.7)	0.93 (0.85 to 1.02)	0.11	1.15 (1.01 to 1.31)	0.03	-	-	-	-
Eating dinner with family									
Never	2,800 (26.2)	(reference)	-	(reference)	-	-	-	-	-
Sometimes	1,620 (15.2)	1.15 (1 to 1.32)	0.05	0.97 (0.81 to 1.17)	0.75	-	-	-	-
Everyday	6,256 (58.6)	1.13 (1.02 to 1.25)	0.02	1.08 (0.94 to 1.23)	0.27	-	-	-	-

[1]Values are n (%) or RR (95% CI)
[2]Other category included those who identified themselves as a student (526[4.9%]) or professional (688[6.4%])

For peer review only

Table S3. Comparison of social determinants of health with stunting (height <145 cm) among late adolescent girls (n=14,771) and adult women (n=10,676) enrolled in the MaPPS Trial at enrolment

**Possible determinants**	**Stunting among late adolescent girls**				**Stunting among young women**			
	Crude		Multivariable adjusted		Crude		Multivariable adjusted	
	OR (95% CI)	*P*	OR (95% CI)	*P*	OR (95% CI)	*P*	OR (95% CI)	*P*
Level 1: Structural factors – socioeconomic status								
Highest level of education								
None	(reference)	-	(reference)	-	(reference)	-	(reference)	-
Primary	0.65 (0.57 to 0.74)	<0.001	0.76 (0.62 to 0.94)	0.01	0.62 (0.51 to 0.74)	<0.001	0.76 (0.63 to 0.93)	0.006
Secondary or higher	0.33 (0.29 to 0.38)	<0.001	0.48 (0.39 to 0.59)	<0.001	0.51 (0.43 to 0.61)	<0.001	0.79 (0.66 to 0.95)	0.01
Occupation								
Unskilled manual labour	(reference)	-	-	-	(reference)	-	-	-
Skilled manual labour	0.56 (0.47 to 0.66)	<0.001	-	-	0.75 (0.61 to 0.91)	0.005	-	-
Within the home	0.69 (0.60 to 0.79)	<0.001	-	-	0.59 (0.49 to 0.71)	<0.001	-	-
Other[1]	0.32 (0.27 to 0.39)	<0.001	-	-	0.45 (0.32 to 0.65)	<0.001	-	-
Religion								
Non-Muslim	(reference)	-	-	-	(reference)	-	-	-
Muslim	0.57 (0.49 to 0.67)	<0.001	-	-	0.74 (0.59 to 0.92)	0.008	-	-
Wealth quintile								
Poorest	(reference)	-	(reference)	-	(reference)	-	(reference)	-
Poor	0.83 (0.72 to 0.97)	0.02	0.91 (0.77 to 1.08)	0.29	0.88 (0.72 to 1.08)	0.22	0.92 (0.73 to 1.17)	0.52
Middle	0.67 (0.57 to 0.78)	<0.001	0.79 (0.65 to 0.96)	0.02	0.65 (0.52 to 0.80)	<0.001	0.70 (0.59 to 0.84)	<0.001
Rich	0.46 (0.39 to 0.55)	<0.001	0.63 (0.49 to 0.81)	<0.001	0.48 (0.38 to 0.60)	<0.001	0.54 (0.40 to 0.74)	<0.001
Richest	0.26 (0.21 to 0.32)	<0.001	0.42 (0.35 to 0.51)	<0.001	0.34 (0.27 to 0.43)	<0.001	0.41 (0.29 to 0.57)	<0.001
Level 2: Intermediary factors - household and personal characteristics								
Marital status								
Married	(reference)	-	-	-	(reference)	-	-	-
Unmarried	0.89 (0.75 to 1.07)	0.21	-	-	0.99 (0.86 to 1.15)	0.93	-	-
Ever been pregnant								
Yes	(reference)	-	-	-	(reference)	-	(reference)	-
No	0.97 (0.74 to 1.27)	0.81	-	-	1.13 (0.96 to 1.32)	0.15	1.27 (1.10 to 1.47)	0.001
Household food security status								
Food insecure	(reference)	-	(reference)	-	(reference)	-	(reference)	-
Food secure	0.58 (0.51 to 0.64)	<0.001	0.77 (0.66 to 0.90)	0.001	0.57 (0.49 to 0.66)	<0.001	0.73 (0.59 to 0.90)	0.003
Level 3: Intermediary factors - health and well-being characteristics								
Perception of own health								
Poor or fair	(reference)	-	-	-	(reference)	-	-	-
Good	0.93 (0.78 to 1.10)	0.38	-	-	0.83 (0.67 to 1.02)	0.07	-	-
Excellent	0.85 (0.70 to 1.04)	0.11	-	-	0.79 (0.62 to 1.00)	0.06	-	-
Experience of depression-like feelings								
Severe or extremely severe	(reference)	-	-	-	(reference)	-	-	-
Moderate	0.93 (0.70 to 1.23)	0.61	-	-	0.98 (0.68 to 1.41)	0.90	-	-
Mild	0.94 (0.71 to 1.26)	0.70	-	-	0.86 (0.58 to 1.26)	0.43	-	-

For peer review only

Possible determinants	Stunting among late adolescent girls				Stunting among young women			
	Crude		Multivariable adjusted		Crude		Multivariable adjusted	
	OR (95% CI)	*P*	OR (95% CI)	*P*	OR (95% CI)	*P*	OR (95% CI)	*P*
None	0.79 (0.62 to 1.00)	0.05	-	-	0.88 (0.64 to 1.19)	0.40	-	-
Experience of anxiety-like feelings								
Severe or extremely severe	(reference)	-	-	-	(reference)	-	-	-
Moderate	0.84 (0.69 to 1.03)	0.09	-	-	0.82 (0.62 to 1.07)	0.14	-	-
Mild	0.96 (0.75 to 1.23)	0.76	-	-	0.99 (0.72 to 1.37)	0.97	-	-
None	0.83 (0.71 to 0.97)	0.02	-	-	0.94 (0.77 to 1.16)	0.56	-	-
Experience of stress-like feelings								
Severe or extremely severe	(reference)	-	-	-	(reference)	-	-	-
Moderate	0.93 (0.64 to 1.34)	0.69	-	-	0.93 (0.58 to 1.49)	0.76	-	-
Mild	1.09 (0.77 to 1.54)	0.63	-	-	0.78 (0.48 to 1.25)	0.30	-	-
None	0.90 (0.68 to 1.20)	0.49	-	-	0.98 (0.68 to 1.40)	0.90	-	-
Level 4: Intermediary factors - actions and practices-related characteristics								
Self-efficacy								
Low	(reference)	-	(reference)		(reference)	-	-	-
Moderate	0.62 (0.56 to 0.70)	<0.001	0.74 (0.64 to 0.84)	<0.001	0.83 (0.71 to 0.98)	0.03	-	-
High	0.59 (0.50 to 0.71)	<0.001	0.74 (0.62 to 0.87)	<0.001	0.78 (0.63 to 0.97)	0.03	-	-
Decision-making autonomy								
All decisions made by family	(reference)	-	(reference)	-	(reference)	-	-	-
Most decisions made by family	0.83 (0.74 to 0.94)	0.003	0.88 (0.76 to 1.01)	0.08	0.99 (0.84 to 1.16)	0.90	-	-
Decisions made jointly with family or autonomously	0.74 (0.62 to 0.89)	0.001	0.76 (0.62 to 0.92)	0.01	0.85 (0.71 to 1.02)	0.09	-	-
Skipping breakfast								
Skips breakfast	(reference)	-	-	-	(reference)	-	-	-
Eats breakfast	0.82 (0.73 to 0.91)	<0.001	-	-	0.79 (0.68 to 0.91)	0.002	-	-
Eating dinner with family								
Never	(reference)		-	-	(reference)	-	-	-
Sometimes	1.09 (0.92 to 1.30)	0.33	-	-	1.19 (0.95 to 1.49)	0.12	-	-
Everyday	0.93 (0.82 to 1.05)	0.23	-	-	1.02 (0.87 to 1.21)	0.79	-	-

[1]Other category included those who identified themselves as a student or profession

For peer review only

Table 1. STROBE-nut: An extension of the STROBE statement for nutritional epidemiology

Lachat C et al. (2016) STrengthening the Reporting of OBservational studies in Epidemiology – Nutritional Epidemiology (STROBE-nut): an extension of the STROBE statement. Plos Medicine 13(6) http://dx.doi.org/10.1371/journal.pmed.1002036 pdf or online version.

Item	Item nr	STROBE recommendations	Extension for Nutritional Epidemiology studies (STROBE-nut)	Reported on page #
**Title and abstract**	1	(a) Indicate the study's design with a commonly used term in the title or the abstract.<br>(b) Provide in the abstract an informative and balanced summary of what was done and what was found.	**nut-1** State the dietary/nutritional assessment method(s) used in the title, abstract, or keywords.	1-3
**Introduction**				
Background rationale	2	Explain the scientific background and rationale for the investigation being reported.		5-7
Objectives	3	State specific objectives, including any pre-specified hypotheses.		6-7
**Methods**				
Study design	4	Present key elements of study design early in the paper.		7
Settings	5	Describe the setting, locations, and relevant dates, including periods of recruitment, exposure, follow-up, and data collection.	**nut-5** Describe any characteristics of the study settings that might affect the dietary intake or	7

For peer review only

Item	Item nr	STROBE recommendations	Extension for Nutritional Epidemiology studies (STROBE-nut)	Reported on page #
			nutritional status of the participants, if applicable.	
Participants	6	a) Cohort study—Give the eligibility criteria, and the sources and methods of selection of participants. Describe methods of follow-up.<br><br>Case-control study—Give the eligibility criteria, and the sources and methods of case ascertainment and control selection. Give the rationale for the choice of cases and controls.<br><br>Cross-sectional study—Give the eligibility criteria, and the sources and methods of selection of participants.<br><br>(b) Cohort study—For matched studies, give matching criteria and number of exposed and unexposed.<br><br>Case-control study—For matched studies, give matching criteria and the number of controls per case.	**nut-6** Report particular dietary, physiological or nutritional characteristics that were considered when selecting the target population.	7-8
Variables	7	Clearly define all outcomes, exposures, predictors, potential confounders, and effect modifiers. Give diagnostic criteria, if applicable.	**nut-7.1** Clearly define foods, food groups, nutrients, or other food components.	8-10

For peer review only

Item	Item nr	STROBE recommendations	Extension for Nutritional Epidemiology studies (STROBE-nut)	Reported on page #
			**nut-7.2** When using dietary patterns or indices, describe the methods to obtain them and their nutritional properties.	
Data sources - measurements	8	For each variable of interest, give sources of data and details of methods of assessment (measurement).Describe comparability of assessment methods if there is more than one group.	**nut-8.1** Describe the dietary assessment method(s), e.g., portion size estimation, number of days and items recorded, how it was developed and administered, and how quality was assured. Report if and how supplement intake was assessed.<br><br>**nut-8.2** Describe and justify food composition data used. Explain the procedure to match food composition with consumption data. Describe the use of conversion factors, if applicable.<br><br>**nut-8.3** Describe the nutrient requirements, recommendations, or dietary guidelines and the evaluation approach used to compare intake with the dietary reference values, if applicable.<br><br>**nut-8.4** When using nutritional biomarkers, additionally use the STROBE Extension for Molecular Epidemiology (STROBE-ME). Report the type of biomarkers used and their usefulness as dietary exposure markers.	8-10

For peer review only

Item	Item nr	STROBE recommendations	Extension for Nutritional Epidemiology studies (STROBE-nut)	Reported on page #
			**nut-8.5** Describe the assessment of nondietary data (e.g., nutritional status and influencing factors) and timing of the assessment of these variables in relation to dietary assessment.<br><br>**nut-8.6** Report on the validity of the dietary or nutritional assessment methods and any internal or external validation used in the study, if applicable.	
Bias	9	Describe any efforts to address potential sources of bias.	**nut-9** Report how bias in dietary or nutritional assessment was addressed, e.g., misreporting, changes in habits as a result of being measured, or data imputation from other sources	8-10
Study Size	10	Explain how the study size was arrived at.		7
Quantitative variables	11	Explain how quantitative variables were handled in the analyses. If applicable, describe which groupings were chosen and why.	**nut-11** Explain categorization of dietary/nutritional data (e.g., use of N-tiles and handling of nonconsumers) and the choice of reference category, if applicable.	8-10
Statistical Methods	12	(a) Describe all statistical methods, including those used to control for confounding<br><br>(b) Describe any methods used to examine subgroups and interactions.	**nut-12.1** Describe any statistical method used to combine dietary or nutritional data, if applicable.	10-12

For peer review only

Item	Item nr	STROBE recommendations	Extension for Nutritional Epidemiology studies (STROBE-nut)	Reported on page #
		(c) Explain how missing data were addressed.<br><br>(d) Cohort study—If applicable, explain how loss to follow-up was addressed.<br><br>Case-control study—If applicable, explain how matching of cases and controls was addressed.<br><br>Cross-sectional study—If applicable, describe analytical methods taking account of sampling strategy.<br><br>(e) Describe any sensitivity analyses.	**nut-12.2** Describe and justify the method for energy adjustments, intake modeling, and use of weighting factors, if applicable.<br><br>**nut-12.3** Report any adjustments for measurement error, i.e,. from a validity or calibration study.	
**Results**				
Participants	13	(a) Report the numbers of individuals at each stage of the study—e.g., numbers potentially eligible, examined for eligibility, confirmed eligible, included in the study, completing follow-up, and analyzed.<br><br>(b) Give reasons for non-participation at each stage.<br><br>(c) Consider use of a flow diagram.	**nut-13** Report the number of individuals excluded based on missing, incomplete or implausible dietary/nutritional data.	12
Descriptive data	14	(a) Give characteristics of study participants (e.g., demographic, clinical, social) and	**nut-14** Give the distribution of participant characteristics across the exposure variables if applicable. Specify if food consumption of	12-13, Table 1

For peer review only

Item	Item nr	STROBE recommendations	Extension for Nutritional Epidemiology studies (STROBE-nut)	Reported on page #
		information on exposures and potential confounders<br>(b) Indicate the number of participants with missing data for each variable of interest<br>(c) Cohort study—Summarize follow-up time (e.g., average and total amount)	total population or consumers only were used to obtain results.	
Outcome data	15	Cohort study—Report numbers of outcome events or summary measures over time.<br>Case-control study—Report numbers in each exposure category, or summary measures of exposure.<br>Cross-sectional study—Report numbers of outcome events or summary measures.		14-16, Table 1
Main results	16	(a) Give unadjusted estimates and, if applicable, confounder-adjusted estimates and their precision (e.g., 95% confidence interval).<br>Make clear which confounders were adjusted for and why they were included.<br>(b) Report category boundaries when continuous variables were categorized.	**nut-16** Specify if nutrient intakes are reported with or without inclusion of dietary supplement intake, if applicable.	14-16, Tables 2-4

For peer review only - http://bmjopen.bmj.com/site/about/guidelines.xhtml

Item	Item nr	STROBE recommendations	Extension for Nutritional Epidemiology studies (STROBE-nut)	Reported on page #
		(c) If relevant, consider translating estimates of relative risk into absolute risk for a meaningful time period.		
Other analyses	17	Report other analyses done—e.g., analyses of subgroups and interactions and sensitivity analyses.	**nut-17** Report any sensitivity analysis (e.g., exclusion of misreporters or outliers) and data imputation, if applicable.	N/A
**Discussion**				
Key results	18	Summarize key results with reference to study objectives.		20
Limitation	19	Discuss limitations of the study, taking into account sources of potential bias or imprecision. Discuss both direction and magnitude of any potential bias.	**nut-19** Describe the main limitations of the data sources and assessment methods used and implications for the interpretation of the findings.	22-23
Interpretation	20	Give a cautious overall interpretation of results considering objectives, limitations, multiplicity of analyses, results from similar studies, and other relevant evidence.	**nut-20** Report the nutritional relevance of the findings, given the complexity of diet or nutrition as an exposure.	20-22
Generalizability	21	Discuss the generalizability (external validity) of the study results.		20-21
**Other information**				
Funding	22	Give the source of funding and the role of the funders for the present study and, if		24

For peer review only

Item	Item nr	STROBE recommendations	Extension for Nutritional Epidemiology studies (STROBE-nut)	Reported on page #
		applicable, for the original study on which the present article is based.		
Ethics			**nut-22.1** Describe the procedure for consent and study approval from ethics committee(s).	24
Supplementary material			**nut-22.2** Provide data collection tools and data as online material or explain how they can be accessed.	Yes

For peer review only
